# VASCilia (Vision Analysis StereoCilia): A Napari Plugin for Deep Learning-Based 3D Analysis of Cochlear Hair Cell Stereocilia Bundles

**DOI:** 10.1101/2024.06.17.599381

**Published:** 2024-06-17

**Authors:** Yasmin M. Kassim, David B. Rosenberg, Alma Renero, Samprita Das, Samia Rahman, Ibraheem Al Shammaa, Samer Salim, Zhuoling Huang, Kevin Huang, Yuzuru Ninoyu, Rick A. Friedman, Artur Indzhykulian, Uri Manor

**Affiliations:** 1 Dept. of Cell & Developmental Biology, University of California San Diego, La Jolla, CA, 92093; 2 Dept. of Cellular and Molecular Biology, University of California, Berkeley, CA, 94720; 3 Dept. of Otolaryngology, University of California, San Diego, La Jolla, CA, 92093; 4 Dept. of Otolaryngology, Kyoto Prefectural University of Medicine, Kyoto, Japan; 5 Dept. of Otolaryngology, Harvard Medical School and Massachusetts Eye and Ear, Boston, MA, 02115; 6 Halıcıoğlu Data Science Institute, University of California, San Diego, La Jolla, CA, 92093

## Abstract

Cochlear hair cell stereocilia bundles are key organelles required for normal hearing. Often, deafness mutations cause aberrant stereocilia heights or morphology that are visually apparent but challenging to quantify. Actin-based structures, stereocilia are easily and most often labeled with phalloidin then imaged with 3D confocal microscopy. Unfortunately, phalloidin non-specifically labels all the actin in the tissue and cells and therefore results in a challenging segmentation task wherein the stereocilia phalloidin signal must be separated from the rest of the tissue. This can require many hours of manual human effort for each 3D confocal image stack. Currently, there are no existing software pipelines that provide an end-to-end automated solution for 3D stereocilia bundle instance segmentation. Here we introduce VASCilia, a Napari plugin designed to automatically generate 3D instance segmentation and analysis of 3D confocal images of cochlear hair cell stereocilia bundles stained with phalloidin. This plugin combines user-friendly manual controls with advanced deep learning-based features to streamline analyses. With VASCilia, users can begin their analysis by loading image stacks. The software automatically preprocesses these samples and displays them in Napari. At this stage, users can select their desired range of z-slices, adjust their orientation, and initiate 3D instance segmentation. After segmentation, users can remove any undesired regions and obtain measurements including volume, centroids, and surface area. VASCilia introduces unique features that measures bundle heights, determines their orientation with respect to planar polarity axis, and quantifies the fluorescence intensity within each bundle. The plugin is also equipped with trained deep learning models that differentiate between inner hair cells and outer hair cells and predicts their tonotopic position within the cochlea spiral. Additionally, the plugin includes a training section that allows other laboratories to fine-tune our model with their own data, provides responsive mechanisms for manual corrections through event-handlers that check user actions, and allows users to share their analyses by uploading a pickle file containing all intermediate results. We believe this software will become a valuable resource for the cochlea research community, which has traditionally lacked specialized deep learning-based tools for obtaining high-throughput image quantitation. Furthermore, we plan to release our code along with a manually annotated dataset that includes approximately 55 3D stacks featuring instance segmentation. This dataset comprises a total of 1,870 instances of hair cells, distributed between 410 inner hair cells and 1,460 outer hair cells, all annotated in 3D. As the first open-source dataset of its kind, we aim to establish a foundational resource for constructing a comprehensive atlas of cochlea hair cell images. Together, this open-source tool will greatly accelerate the analysis of stereocilia bundles and demonstrates the power of deep learning-based algorithms for challenging segmentation tasks in biological imaging research. Ultimately, this initiative will support the development of foundational models adaptable to various species, markers, and imaging scales to advance and accelerate research within the cochlea research community.

## 1 Introduction

**Figure 1.**
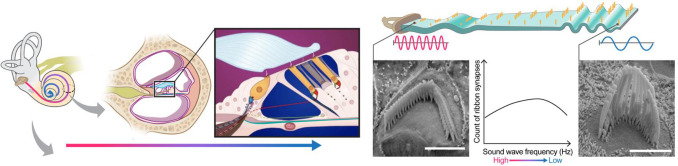
The anatomy of the cochlea^33^. Sensory hair cell stereocilia sit along a tonotopic axis that follows the length of the spiral-shaped cochlea with a striking pattern of increasing stereocilia lengths from the base to the apex of the cochlea. The morphological and spatial features of cochlear hair cell stereocilia follow extremely predictable tonotopic patterns between individuals and species: Stereocilia lengths increase as a function of position along the tonotopic axis of the cochlea, which in turn is reflective of the frequency of sound they are tuned to detect. Thus, the relationship between the morphological and spatial features of these cells and their function are relatively well-defined compared to many other biological systems. Due to their highly patterned organization, cochlear tissues thus present a particularly striking opportunity for automated computer vision tasks.

The last decade has seen a phenomenal advancement in Artificial Intelligence (AI), giving birth to countless architectures, backbones, and optimization techniques that are being continually refined^1^,^2^. Despite the potential to revolutionize computer vision tasks, many researchers in biological sciences still use computationally primitive and labor-intensive approaches to analyze their imaging and microscopy datasets. One major area where AI-based computer vision tasks could have maximal impact is in pre-clinical auditory research. In our lab, cutting-edge imaging tools and approaches are developed and utilized to gain insights into the structure-function relationship of subcellular structures in sensory hair cells with molecular resolution. These rich imaging datasets present an opportunity to understand how sensory hair cells function in health and disease, including cases of congenital and age-related hearing loss^3–9^. The structural attributes of the cochlea are quite uniform across species. For example, the famously snail-shaped cochlea is governed by a so-called “tonotopic” organization with a gradient of morphological features that change from one end of the cochlea to the other. The result of this organization is that each position along the length of the cochlea dictates the characteristic frequency of sound to which the tissue responds, therefore directly correlating position with function. In other words, the sensory hair cell organelles called “stereocilia” or “bundles” follow a pattern of increasing lengths as a function of position along the length of the cochlea^10^,^11^. The cartoon in Fig 1 illustrates how the length of hair cells corresponds to specific frequencies, when the cochlea is unrolled for illustrative purposes. Hair cells are organized tonotopically. Hair cells at the base of the cochlea respond best to high-frequency sounds, while those at the apex respond best to low-frequency sounds. The physical and functional properties of these structures vary systematically along the tonotopic axis of the auditory system. For example, hair bundles might be longer or shorter, or synapses might be more or fewer or have different properties, depending on whether they are associated with high-frequency or low-frequency regions. The association between these attributes and their purpose makes the cochlea an ideal system for investigating the potential of AI in biological image analysis.

In this study, we leverage Napari, an open-source Python tool^12^, as the foundation for developing our plugin, chosen for its robust viewer capabilities. The Napari platform has seen a growing number of plugins^13–26^ designed to address various biomedical challenges. However, our plugin represents the first such tool tailored specifically for the ear research community, enabling high-precision AI-based 3D instance segmentation and measurement quantification of stereocilia cells.

Previous studies have explored manual segmentation of stereocilia images to quantify measurements for specific research objectives^9,27^. Others have attempted to automate this process using traditional intensity-based methods^28^. However, these traditional approaches typically lack the capabilities provided by AI technologies, which can autonomously extract features and identify regions without relying exclusively on intensity cues. This gap underscores the critical need for advanced AI-driven segmentation techniques that utilize a broader array of features, thereby enhancing both the accuracy and the detail of the segmentation. We identified a few machine learning and deep learning publications. Urata et. al.^29^ relies on template matching and Machine learning-based pattern recognition to achieve detection and analysis of hair cell positions across the entire longitudinal axis of the organ of Corti. Buswinka et al.^30^ develops a software tool that utilizes AI capabilities; however, this tool is limited to providing bounding box detection and does not offer 3D instance segmentation for the bundles. This significantly hampers the tool’s ability to provide shape-level descriptors and accurate measurements. Cortada et al.^31^ use stardist deep learning architecture^32^ to segment the hair cells, however, they don’t provide 3D instance segmentation, they only provide 2D detection based on max projection.

VASCilia, see Fig 14, equips users with a comprehensive suite of tools for advanced image analysis: 1. Operations such as reading, preprocessing, trimming unwanted stack regions, and rotating images. 2. Accurate 3D segmentation masks with associated IDs for detailed analysis. 3. Calculations of various measurements including volume, centroid, and surface areas. 4. Automatic determination of lengths from the tip of the bundle to the base. 5. Clustering of bundles into four rows: IHC, OHC1, OHC2, and OHC3 by utilizing K-means, Gaussian Mixture Models, and deep learning techniques. 6. Computation of protein intensities to assess expression levels. 7. Determination of bundle orientation. 8. An AI model predicts the cochlear region (BASE, MIDDLE, APEX) from which the stack originates. 9. A training section allows other laboratory users to fine-tune our AI models with their data according to different staining protocols and experimental conditions. 10. A mechanism to upload and re-store all the analytical data and intermediate steps from a single pickle file, ensuring comprehensive data management and reproducibility. We anticipate that our AI-enhanced plugin will streamline the laborious process of manual quantification, offering high-throughput with solid interpretation.

## Results

### 1.1 A 3D instance segmentation pipeline for stereocilia bundles

**Figure 2.**
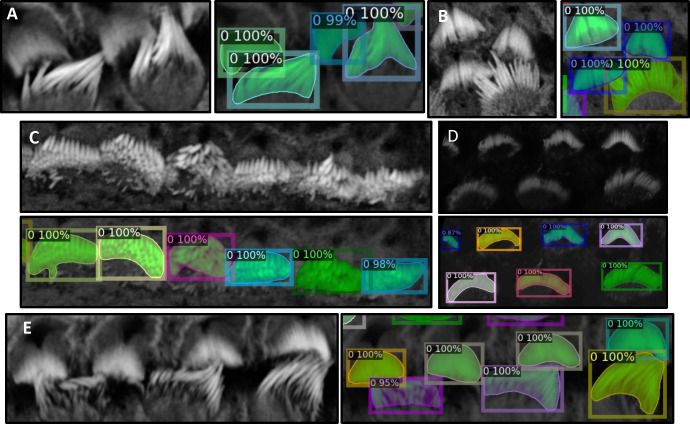
Figures A, B, and E illustrate instances where inter-row stereocilia bundles are in close proximity, yet the algorithm successfully separates them, demonstrating robust performance. In Figure D, despite the raw image being notably dark, the algorithm remains effective in detecting separate bundles. Figure C highlights a scenario where intra-row bundles are tightly clustered. Nonetheless, the algorithm efficiently distinguishes each bundle, underscoring its capability to resolve complex spatial relationships within the samples.

3D confocal image stacks of phalloidin-stained cochlea tissues presents multiple challenges that render the 3D segmentation of stereocilia bundles a demanding and difficult task. These challenges include non-specific staining, inter-row confluence, intra-row density, noise, intra-cell signal drop-out, inconsistent signal intensity, and tonotopic shape heterogeneity, see Fig 2. Each of these factors significantly complicates the segmentation process and highlights the complexity of accurately identifying and delineating stereocilia bundles, particularly as a 3D instance segmentation task.

Our method utilizes 2D detection on individual frames, which is then followed by reconstructing the 3D object through a multi-object assignment algorithm. For detailed methodology, refer to Sections 3.2.

**Figure 3.**
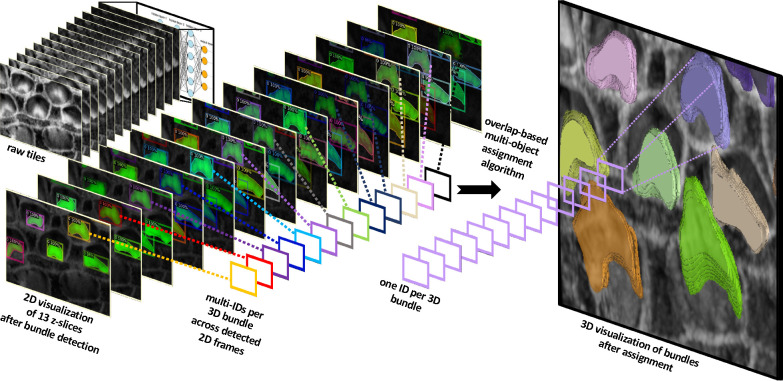
This figure demonstrates the algorithm’s capability to detect stereocilia bundles in 2D across successive frames, assigning unique IDs to each detected object within the 2D plane. The variation in IDs across frames reflects the independent detection process in each 2D frame. Subsequently, a multi-object assignment algorithm intervenes to reconcile these IDs, effectively re-assigning them to maintain consistency across frames. This step is crucial for reconstructing accurate 3D objects, ensuring that each bundle retains a consistent ID throughout all frames. This approach not only enhances the continuity and integrity of the 3D model but also facilitates a robust analysis of stereocilia bundle morphology.

We partitioned the dataset into training, testing, and validation groups at the stack level to avoid data leakage and mixing frames from different stacks. The training set includes 30 stacks, while the validation contains 5 stacks. Ten stacks are withheld for testing and evaluation. These stacks are categorized into two types: typical and complex cases. Six stacks fall into the typical category, featuring images that are easier to segment due to less overlap among the bundles and well-separated bundle rows. The remaining four stacks are classified as complex cases. These contain structures that pose significant challenges for segmentation due to various factors. The objective of this partitioning is to evaluate the decrease in performance when processing challenging cases and check the robustness of the detection algorithm. Figures 2[A–E] illustrate the model’s proficiency in accurately detecting the cells and demonstrate the model’s effectiveness in overcoming the inherent challenges associated with this task. Fig 3 displays 13 frames from a single dataset or stack that has been segmented in 2D, followed by the application of a multi-object assignment algorithm to reconstruct each 3D object. Details about the algorithm can be found in Section 3.2.

After completing the training process, we employed the trained model to segment each bundle in the images from the testing set. To evaluate performance, we calculate the Intersection over Union (IoU) for 3D volumes by comparing predicted and ground truth segmentation masks. Additionally, we compute the F1 measure, accuracy, precision, and recall for the predictions against the ground truth. The evaluation process iterates through unique labels found in both ground truth and predicted volumes, excluding the background label, and computes the IoU for corresponding labels. This method tracks true positives (TP), false positives (FP), and false negatives (FN) across varying IoU thresholds. For instance, if one 3D bundle in the ground truth overlaps with two bundles in the predictions, the algorithm considers the one with the largest overlap as a TP and the unmatched one as a false positive. We conduct evaluations across a range of IoU thresholds, from 0.1 to 1 in steps of 0.05.

$$IoU=\frac{\left|A\;\cap \;B\right|}{\left|A\;\cup \;B\right|},F1-Score=2\times \frac{\;\text{Precision}\;\times \;\text{Recall}\;}{\;\text{Precision}\;+\;\text{Recall}\;},Accuracy=\frac{TP\;+\;TN}{TP\;+\;TN\;+\;FP},Precision=\frac{TP}{TP\;+\;FP},Recall=\frac{TP}{TP\;+\;FN}$$

TP,FP, and FN are the number of true positives, false positives, and false negatives, respectively. In object detection, we do not consider the True Negative (TN) because it refers to correctly labeling background pixels or non-object pixels as such. Since the background or non-object areas can be vast, focusing on TNs would skew the performance metric towards the most common class (background), which does not provide useful information about the model’s ability to detect objects of interest.

**Figure 4.**
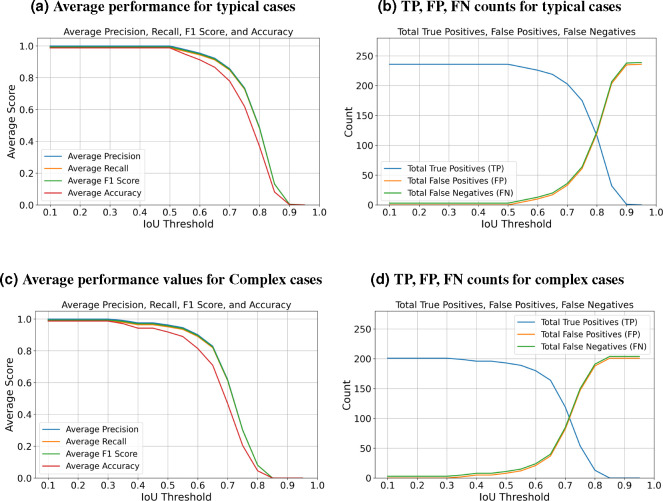
This table presents detailed performance indicators: Precision, Recall, F1 Score, and Accuracy, alongside True Positives (TP), False Positives (FP), and False Negatives (FN). The metrics are evaluated across two categories: typical cases that do not present extreme challenges, and complex cases affected by variable conditions

**Figure 5.**
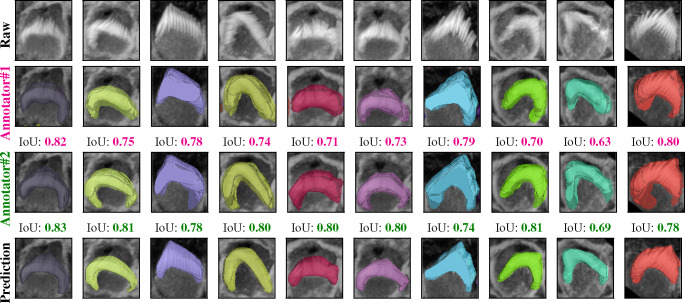
Comparison of raw crops (first row), GT masks for the first annotator (second row), GT masks for the second annotator (third row), and the Predicted masks (forth row) for 10 different crops from the same stack, the IoU score underneath the crops indicate the Intersection Over Union score between the 3D GT masks and the 3D predicted masks. We examined the margin of error between two annotators for a single 3D stack. The average overlap was 0.70 between two annotators, 0.74 between one annotator and the prediction, and 0.76 between the prediction and the second annotator over 47 instances across the 3D stack.

Figure 4(a) indicates that the average F1-measure and accuracy are 99*.*4% and 98*.*8%, respectively, at an IoU of 0*.*5 for typical test set. In contrast, Figure 4(c) reveals that at the same IoU level, the average F1-measure and Accuracy decrease to 95*.*7% and 91*.*9% for complex test set. Maintaining the F1 measure and accuracy above 90% demonstrates that the algorithm effectively handles the significant challenges present in the images. We can observe from Figures 4(b) and (d) that the total number of True Positives (TPs) remains high until the IoU reaches 0.8 and 0.72, respectively. Beyond these thresholds, there is a noticeable increase in both False Positives (FPs) and False Negatives (FNs). When you observe that TPs remain high until IoU thresholds of 0.8 and 0.72, this indicates that the algorithm is quite effective at correctly identifying and matching relevant objects or features in the dataset up to these points. The increasing errors (FPs and FNs) at higher IoU thresholds indicate that the algorithm struggles with precision and recall balance as criteria become more strict. Figure 5 showcases ten crops of stereocilia bundles: the first row displays the raw crops, the second and third rows features ground truth manual annotations made by two annotators in CVAT^34^, and the forth row presents our predicted 3D volumes for each bundle. It is evident that most bundles achieve Intersection over Union (IoU) scores between 70 and 80, which visually suggest near-perfect alignment. The dataset has been annotated by five different annotators. To put the accuracy of our predictions in a more meaningful context, we investigated the margin of error between annotators on the same stack. We found that the average intersection over union (IoU) for 47 instances from a single stack between two annotators is 0.70. Strikingly, the model achieved a higher average IoU of 0.76 with the first annotator and 0.74 with the second annotator. This suggests that the model is performing with similar or higher reliability as human proofread annotations. We speculate this is due to the model’s ability to effectively average the experience of multiple annotators (five annotators in our case). These results also highlight the difficulty of achieving accuracies beyond a certain amount, as even two human expert annotators only agreed 70% on pixel-based 3D annotations.

### 1.2 VASCilia deep learning-based prediction tools and measurements

The Napari plugin equips the users with essential measurements and deep learning-based tools tailored for analyzing stereocilia bundles. Users can accurately determine the volume, surface area, and centroids of segmented regions. Beyond these fundamental measurements, the plugin includes specialized metrics critical to hair cell research, such as calculating stereocilia bundle heights, predicting the region of the cochlea from which the stack is taken (Base, Middle, Apex), clustering hair cells into four distinct rows, assessing fluorescence intensity within the bundle, and determining bundle orientation with respect to the planar polarity axis of the cochlea. These features are designed to support the advanced analytical needs of the hair cell research community.

#### 1.2.1 Stereocilia bundle height measurement

Hair cell stereocilia bundles are essential for hearing, as they convert mechanical sound vibrations into electrical signals that the brain interprets as sound. Many deafness mutations cause bundle defects, include a failure to elongate. Accurately and consistently measuring stereocilia bundle heights in 3D fluorescence images is therefore critical, but unfortunately laborious and costly. The steps for accurate and automated bundle height measurements involves calculating the distance from the tip to the base of stereocilia bundles, which can be summarized with these steps:

1. Iterate through each connected component in the labeled volume. Skip the background and any filtered components.
2. For each relevant component, identify all voxel coordinates that belong to the bundle.
3. Create a binary sub-volume for the current component where the component’s voxels are marked as one, and all othersare zeros.
4. Project the binary sub-volume along the z-axis to reduce it to a 2D projection by summing along the z-dimension andthen applying a threshold to ensure the projection remains binary (values greater than 1 are set to 1).
5. Locate the highest (tip) and lowest (base) y-coordinates in the 2D projection that have nonzero values:
  - Find the highest point by identifying the minimum y-coordinate value that corresponds to 1 in the projection.
  - Find the bottom-most point by tracing downward from the centroid of the projection until reaching a y-coordinate with a value of 0, then stepping back to the last non-zero coordinate.
6. Determine the z-coordinates of these points in the original 3D volume:
  - For the highest point, find the z-coordinates where the voxel at the identified (x, y) location is 1.
  - For the lowest point, determine the z-coordinate by identifying the first z-slice that contains any part of the component.
7. Store the coordinates of the highest and lowest points. These are used for calculating the 3D Euclidean distance between the tip and the base of the bundle.
8. Calculate the distance using the Euclidean distance formula between the stored highest and lowest points.
$$\text{Distance}\;=\sqrt{{\left(\left(x_{2}-x_{1}\right)\right)}^{2}+{\left(\left(y_{2}-y_{1}\right)\right)}^{2}+{\left(\left(z_{2}-z_{1}\right)\right)}^{2}}$$
  where (x1,y1,z1) and (x2,y2,z2) are the coordinates of the first and second points in these dimensions. Note that a scaling factor should be used for each dimension to ensure the calculated distance accurately reflects the true spatial separation between the two points in real physical units.

**Figure 6.**
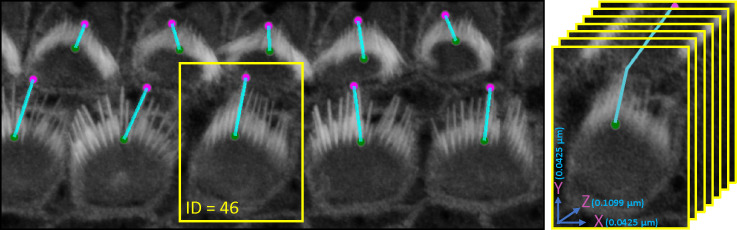
An illustration of the plugin’s automated method for measuring the distance from the tip to the bottom of stereocilia bundles. Users can adjust the positions of the upper and lower points. Upon making these adjustments, the plugin’s listeners automatically detect the changes, redraw the connecting line, and recalculate the distance accordingly. Left: shows the 3D visualization using Napari. Right: demonstrates how the distance is computed from x, y, and z, considering the physical resolution in microns.

One limitation of this algorithm is that it may not align the distance measurements along the tallest stereocilium within the bundle, which may be desirable. To address this, the plugin includes event-handlers that facilitate user corrections of the highest and lowest points in each bundle. This adjustment can be easily made through the intuitive 3D viewer within the Napari interface, enabling researchers to refine their measurements as needed. The plugin provides users with a CSV file that details the height measurements for each bundle, each tagged with a corresponding ID. Fig 6 illustrates the computation and Tables 1 and 2 present bundle height validation as measured between VASCilia and two human expert annotators. We found it takes an average of 5.5 minutes to manually annotate each bundle height using Fiji. In contrast, VASCilia significantly reduces this time to just one second to press a button to calculate the height of all the bundles in a single 3D stack (as many as 55), with at most five minutes needed for refining the base and top points of <10 bundles per stack if necessary. Thus, measuring the length for 10 stacks, each containing 50 cells, would take 2,750 minutes (approximately 46 hours) with Fiji, compared to only 50 minutes with VASCilia. We conducted both paired t-tests and Pearson correlation analyses to evaluate the agreement between VASCilia and human expert annotators. The Pearson correlation analysis showed very strong positive relationships: 0.939 (p < 0.001) between VASCilia and annotator 1, 0.721 (p < 0.001) between VASCilia and annotator 2, and 0.830 (p < 0.001) between annotators 1 and 2. The paired t-test results indicate no statistically significant differences between the measurements obtained by VASCilia and annotator 1 (t = 1.613, p = 0.125) or annotator 2 (t = 0.904, p = 0.379). No significant difference (t = 0.180, p = 0.860) was observed between the measurements from observers 1 and 2. Together, our findings suggest VASCilia performs comparably to human expert annotators, and that the annotators themselves are consistent in their measurements, see Table 3.

#### 1.2.2 Tonotopic Position Prediction (BASE, MIDDLE, APEX)

**Figure 7.**
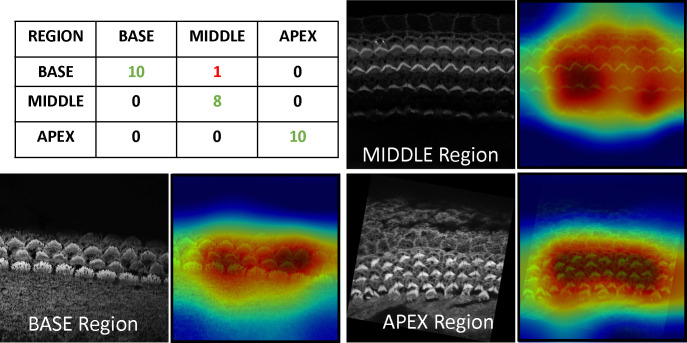
Top left: co-varience matrix for model prediction, others: Grad-CAM Visualization for Base, Middle, and Apex; Resized and Overlaid Response from the Last Convolutional Layer Highlighting Focus on Bundles During Decision Making

One of the fascinating features of cochlear hair cell bundles is the so-called “tonotopic” organization, wherein the bundle heights follow a pattern of increasing lengths as a function of position along the length of the cochlea, see Fig 1. Since the frequency of sound detected follows a similar pattern along the length of the cochlea, we were motivated to generate a model that can accurately assess the tonotopic position of the imaged region of the cochlea. To conduct this task, We trained a classification CNN. For more details about the architecture, see Section 3.3. For training and validation, our dataset comprises 36 3D stacks from the BASE region containing 535 images, 35 3D stacks from the MIDDLE with 710 images, and 38 3D stacks from the APEX with 651 images. For testing the model, we used ten stacks from BASE, nine from MIDDLE, and ten from APEX that were withheld from the training data. The classification of each stack as BASE, MIDDLE, or APEX is determined through a majority voting mechanism applied across all frames of the associated stack, starting from the median and extending over a length of 13 frames. This method has demonstrated robust performance, achieving a subject-based accuracy of 97%; 28 out of 29 stacks were correctly identified. The single observed misclassification in our testing data involved a stack from the MIDDLE being incorrectly predicted as BASE, which we speculate is due to the close proximity and overlapping characteristics of these two cochlear regions. The covariance matrix for the regions BASE, MIDDLE, and APEX is in Fig 7.

To gain insights into the focus areas of our model during prediction, we employed Gradient-weighted Class Activation Mapping (Grad-CAM), a powerful visualization tool used to explain deep learning-based predictions. Grad-CAM helps visually identify which parts of an image are pivotal for a model’s decision-making process. Grad-CAM works by capturing the gradients of the target label, which is the correct class in this scenario, as they propagate back to the final convolutional layer just before the softmax. It then uses these gradients to weigh the convolutional feature maps from this layer, generating a heatmap that visually emphasizes the image regions most critical for predicting the class label. This serves as a sanity check to confirm the model’s reliance on accurate features for its predictions. Interestingly, Grad-CAM revealed that the model predominantly focuses on the hair cell bundles to distinguish between the BASE, MIDDLE, or APEX regions. Grad-CAM visualization is shown in Fig 7.

Future directions include exploring the possibility of developing a foundation model capable of accurately predicting cochlear regions across various datasets with different staining and imaging protocols. This effort would undoubtedly require extensive data collection. We believe this work demonstrates that this task is feasible. The Napari-based graphical user interface and interactive visualization tools presented here should help make these methodologies and trained models accessible to the average researcher. We hope these results help motivate collaborative efforts to gather and share more comprehensive imaging data within the inner ear hair cell research community.

#### 1.2.3 Fluorescence Intensity Measurements

Understanding signal levels whether they are one or multiple proteins or a particular stain is crucial in various research fields, including cochlear function. The plugin enables the user to get a precise quantification for the signal to allows researchers to gain deeper insights into its function and potential involvement in hearing loss.

The plugin has a mechanism to calculate both the mean and total fluorescence intensity, subtracting the background intensity to correct for ambient noise. This is achieved by superimposing the 3D segmented masks onto the corresponding fluorescent image slices and aggregating the fluorescent intensities across the z-stack.

**Figure 8.**
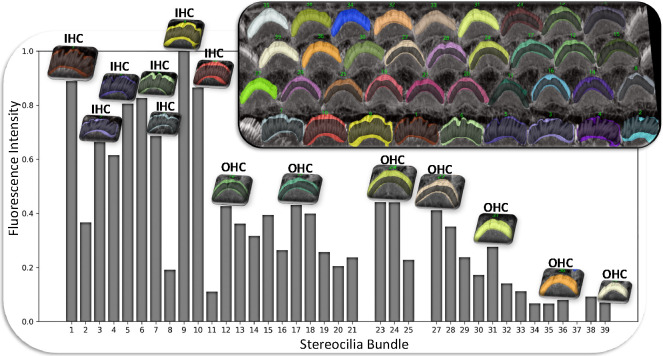
The provided plots demonstrate the utility and ease with which users can compare data between bar plots and actual cellular images within the plugin. This visual comparison simplifies the interpretation of differences, enhancing the user’s ability to discern and analyze variations in signal intensity across different cell types directly within the interface. In this example, we explored phalloidin staining and observed that IHCs exhibit higher fluorescence intensity levels compared to OHCs. This likely reflects differences in the amount of filamentous actin in IHC vs. OHC bundles, and may serve as a robust indicator for machine learning models to differentiate between IHCs and OHCs.

The resulting intensities are normalized and plotted, allowing for comparative analysis across different cell types such as inner hair cells (IHCs) and outer hair cells (OHCs) in various categories (OHC1, OHC2, and OHC3). Data for each cell type is stored and visualized through histograms that display the mean and total intensity distributions to provide insights and observation about the protein or signal expressions across the cochlear architecture. For more clarification, users will obtain plots for each cell type, resulting in a total of 20 outputs per stack (10 plots and 10 CSV files). See Fig 8 for the total intensity histogram bar for all cells from the Napari plugin, which accumulates the Phalloidin signal in the green channels of the stack. The plugin can also be used to analyze and generate plots for other proteins in different channels as needed by the study. Here, we are only considering the stain itself.

#### 1.2.4 High-Throughput Computation of Bundle Orientation

Understanding the precise orientation of stereocilia bundles in the cochlea is critical for deciphering the intricate mechanics of hearing. These hair-like structures are arranged in a “V” shape due to a phenomenon called planar cell polarity (PCP). PCP ensures that neighboring hair cells and their stereocilia bundles are aligned in a specific direction.

and it plays an essential role in orchestrating the precise orientation of stereocilia bundles, which is fundamental for both the directional sensitivity and efficient mechanotransduction necessary for our sense of hearing. Studying and potentially manipulating PCP holds immense potential for developing better hearing aids, diagnosing hearing loss with greater accuracy, and understanding the mechanisms behind various auditory disorders. In addition, it provides information on the effects of genetic mutations and environmental factors on hearing and facilitates comparative studies that explore evolutionary adaptations in hearing.

**Figure 9.**
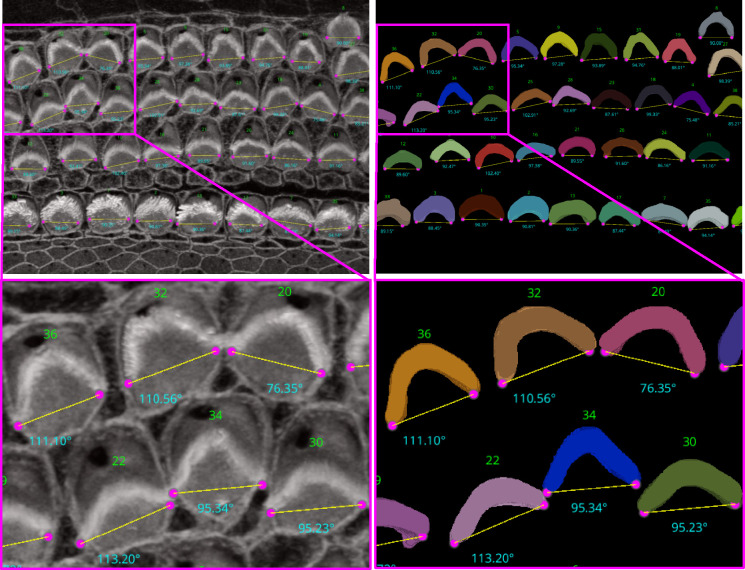
Automated Computation of Stereocilia Bundle Orientation Using a Height-Only Method. Top Left: illustrates the bundle orientations superimposed on the raw data, Top right: displays the 3D segmentation masks with bundle orientation highlighted. Bottom Right and Left are cropped regions for a closer look.

**Figure 10.**
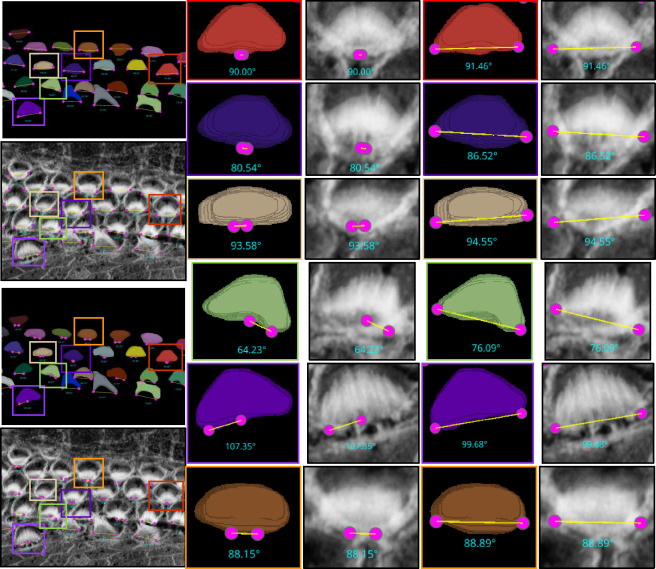
The limitations of the Height-Only orientation computation. While the Height-Only method excels with cells exhibiting a clear V-shape Fig 9, it struggles with some apical region hair cells that lack this distinct structure. To address these challenges, we developed the Height and Distance approach, which effectively handles a wider variety of cell shapes. The visual comparison includes the frames from which the crops are taken (first column), Height-Only results superimposed on the 3D segmented labels (second column) and the original images (third column), alongside the height and distance results superimposed on the 3D segmented labels (fourth column) and the original images (fifth column). We observe that the Height and Distance method overcomes the limitations of the Height-Only computation.

The time-consuming nature of the manual PCP measurement methods highlights the need for more automated and efficient techniques to further advance our understanding of this critical process. We identified an automated Fiji plugin, PCP Auto Count, for quantifying planar cell polarity and cell counting^35^. This plugin relies on identifying “chunks” (the hair cell apical surface) and “caves” (the fonticulus) to measure the orientation between their centers. However, this approach was not effective with our images due to the absence of clear caves, especially in low-contrast images. The reliance on distinct caves for orientation measurement poses a limitation for datasets where such features are not consistently visible or distinguishable. For this reason, we developed two automated mechanisms, “Height only” and “Height and Distance”, in our plugin to automatically obtain the orientation of the stereocilia bundles based on our 3D segmentation masks:

For the ‘Height only’ approach, our method pinpoints and records the lowest points on both the left and right sides of the centroid within the 2D projection. Conversely, the ‘Height and Distance’ method not only locates these points but also measures distances from a specific peak point to identify the most remote and lowest points on each side. While the ‘Height only’ approach functions perfectly with cells that exhibit a V-shape structure as clear in Fig 9, it is less effective for cells with atypical shapes, commonly encountered in the apex region. This limitation led to the development of the ‘Height and Distance’ method, which reliably accommodates cells with irregular shapes, see Fig 10.

Following the orientation calculations, the script proceeds to generate lines and angles for each region. It connects the corresponding left and right points and calculates angles using the arctan2 function for deltaY and deltaX, providing a precise angular measurement. These orientation points, lines, and computed angles are then visualized in the Napari viewer through distinct layers, specifically designated for points, lines, and text annotations. This visualization is enhanced with carefully chosen colors and properties to ensure clarity and optimal visibility.

For comprehensive data analysis and record-keeping, a DataFrame containing the IDs and corresponding angles is compiled and exported to a CSV file. To ensure dynamic interactivity within our visualization tool, event listeners are embedded to reflect any adjustments in orientation points directly on the orientation lines and measurements.

#### 1.2.5 Bundle Clustering (IHC, OHC1, OHC2, OHC3)

The categorization of hair cells into IHC, OHC1, OHC2, and OHC3 in ear biology is not merely a morphological distinction but is deeply tied to their physiological roles, susceptibility to damage, and their integral role in the auditory system. This detailed classification enables more nuanced research exploration, diagnostics, and treatments in audiology and related biomedical fields. Identifying these rows manually is a time-consuming and laborious process. For this reason, we have enhanced our plugin with mechanisms to identify the four rows using three strategies: KMeans, Gaussian Mixture Model (GMM), and deep learning. As a result, this can significantly automate the process, providing high-throughput results for all cells in the current stack in just one second. 1. KMeans: We have implemented KMeans clustering due to its simplicity and efficiency in partitioning data into distinct clusters based on their features. Specifically, we use KMeans to categorize hair cells into four clusters, leveraging attributes such as proximity and density for grouping. This method calculates the centroids of the data points and iteratively minimizes the distance between these centroids and their assigned points, making it highly suitable for fast, general clustering tasks. KMeans proves particularly effective when IHCs and OHCs are distinctly separated and organized in linear rows, especially as it performs optimally without the presence of outliers or significant overlap between rows. 2. Gaussian Mixture Model: The Gaussian Mixture Model is a probabilistic model that assumes all the data points are generated from a mixture of several Gaussian distributions with unknown parameters. Applied to the clustering of hair cell rows. By fitting the GMM to the ‘y’ coordinates of hair cell endpoints, we can predict the cluster for each cell, which aids in categorizing them into distinct groups based on their vertical spatial alignment. This approach is particularly useful in scenarios where the clusters might have different variances, which is often the case in biological tissues. The ability of GMM to accommodate mixed distribution models helps in accurately classifying cells into four predetermined clusters. The Gaussian Mixture Model (GMM) offers flexibility over KMeans by accommodating clusters of varying shapes, sizes, and densities, which is crucial in biological data where such variations are prevalent. Unlike KMeans, which assumes spherical clusters and hard partitions, GMM models the probability of each point’s membership in potential clusters, allowing for soft clustering. 3. Deep Learning: While KMeans and GMM provide robust and instant results for many samples, see first row of Fig 12, however, these statistics-based methods can struggle with complex configurations, particularly in samples with outliers, overlapping rows, or non-linear, curvy arrangements. For these challenging scenarios shown in second and third row of Fig 12, we have developed a deep learning approach that utilizes multi-class classification to accurately identify and categorize all hair cell bundles, effectively handling the spatial and structural complexities inherent in such data. Check Section 3.4 about the architecture and model training details.

For inference, we focused exclusively on stacks from the Apex region, where the cells frequently display non-linear growth, are closely packed, and often overlap, leading to higher error rates with Kmeans and GMM methods. Conversely, cells in the Base and Middle regions are typically well-aligned. We conducted two experiments: In the first, we tested all Apex data, including 41 datasets from both training and testing phases. In the second experiment, we evaluated 25 stack datasets that were not included in the training set to verify the model’s performance on both seen and unseen data.

Figure 13(a) and (b) illustrate the error rates by method and dataset to assess performance per sample. Figures 13(c) and (d) present heatmaps of the errors by method and sample, offering a more visual representation of performance with a color map transitioning from black at the bottom to yellow at the top. These figures highlight the pronounced error frequency in the Kmeans and GMM methods compared to our deep learning approach. Figures 13(e) and (f) display the cumulative errors, providing insight into how errors accumulate linearly per dataset and method. It is notable that the errors with Kmeans and GMM, represented in blue and brown, show a steep increase, whereas the deep learning method demonstrates a more consistent error rate. In Experiment 1, across a total of 1824 cells, the error counts were 202 for Kmeans, 257 for GMM, and 12 for Deep Learning. In contrast, Experiment 2 involved 1102 cells, with error counts of 136 for Kmeans, 169 for GMM, and 11 for Deep Learning. We also conducted a one-way ANOVA (Analysis of Variance) test. This statistical test is used to determine whether there are statistically significant differences between the means of three or more independent (unrelated) groups. We found that there is significant differences between groups with F-value equal to 10.45, A higher F-value indicates a greater degree of variation among the group and P-value equal to 0.0001 which reject the null hypothesis of the ANOVA test. The null hypothesis for an ANOVA test states that all group means are equal.

## 2 Discussion

VASCilia, displayed in Fig 14, stands out in several unique ways: 1) It is the inaugural Napari plugin and software analysis tool designed specifically for 3D segmentation of stereocilia bundles, equipped with comprehensive analysis capabilities and machine learning models tailored for the inner ear research community. 2) It has a user-friendly interface that is easy to install and navigate. 3) The flexibility of VASCilia allows other labs to use it directly for similar image datasets including species ranging from mice to humans, or fine-tune the existing model with their data using the training section available in the tool 4) Most notably, VASCilia offers a distinctive feature whereby analysis results can be uploaded and reviewed later by the analyst, collaborators, or principal investigators, facilitating the collaboration and the review processes. For the next two sections, we will share more details about the VASCilia Workflow and about the training section.

### 2.1 VASCilia Workflow and Features

VASCilia begins by initializing all necessary properties for comprehensive analysis, invoking a function called ‘initialize_ui’ to set up all plugin buttons and prepare the user interface for operation.

Users can either open and preprocess a new dataset using the ‘Open Cochlea Datasets and Preprocess’ button or upload an analyzed dataset with the ‘Upload Processed Stack’ button. The plugin supports both Zeiss (.czi) and Leica (.lif) file formats, with flexibility to incorporate additional formats. It starts by reading metadata and extracting physical resolution variables for later use in length computations, applies Contrast Limited Adaptive Histogram Equalization for pre-processing, and displays the channels as layers in Napari.

Users initiate their analysis by manually trimming the stack to isolate the Z region of interest, rotating the stack to align it with the tissue’s planar polarity axis, and then proceeding with segmentation, reconstruction, and visualization. Subsequently, users can remove unwanted regions, perform measurements, calculate lengths from top to bottom of stereocilia bundles, compute protein intensity, predict the origin of the stack, determine orientation, and cluster rows into four categories (IHC, OHC1, OHC2, OHC3). All these functionalities depends upon the invoke a function called ‘save_attributes,’ which efficiently saves all variables used in the analysis in a pickle file. This storage action is implicit, requiring no manual save operations from the user, thus enabling the upload function to retrieve and apply these variables for visualization in the viewer. This setup enables analysts to resume or revisit their analysis at a later time.

After analyzing several datasets, users can compile all generated CSV files related to length computations, orientation, and protein responses to analyze and plot observations necessary for their studies. Furthermore, users can download segmented regions to gain insights into how bundles correlate with different regions, enhancing the research’s depth and applicability.

The user can reset Napari for analyzing a new dataset using the ‘Reset’ button. This feature saves all current variables and then clears them along with all existing layers. This process ensures that the plugin is thoroughly prepared and optimized to handle a new dataset to allow smooth transition between tasks.

### 2.2 User-Enhanced Accuracy in Automated Measurements

In VASCilia, aside from the segmentation tasks, all automated measurements can be fine-tuned interactively by the user. The plugin is equipped with listeners that actively monitor user interactions related to the adjustment of points, that affect length and orientation computation. This feature ensures that any automated measurements can be further modified to reflect the precise requirements of the user.

Furthermore, for clustering tasks, VASCilia empowers users to intervene when automated clustering may not align perfectly with the expected outcomes. Users have the flexibility to reassign elements between clusters, correcting any discrepancies. This capability allows for significant refinement of the clustering results to ensure that the automated processe is complemented by user expertise and judgment.

### 2.3 Training section

VASCilia is fundamentally designed to obtain 3D segmentation of stereocilia bundles, an essential step for all subsequent measurements within the plugin. To ensure adaptability and utility across various labs, VASCilia includes a feature allowing users to fine-tune the existing segmentation model with additional images from their specific datasets. This adaptability is crucial for handling variations in staining techniques, settings, and image dimensions such as height, width, and resolution. This feature is both user-friendly and vital for broadening the plugin’s applicability. The training module within VASCilia features seven buttons for ease of use:

#### Create/Save Ground Truth:

allows users to generate a new layer, named ‘Ground Truth’, within the plugin. This button also enables saving of manually annotated data directly within this layer.

#### Copy Segmentation Masks to Ground Truth:

simplifies the annotation process by transferring existing segmented 3D masks to the Ground Truth layer. This functionality allows users to make precise adjustments to the model’s initial predictions rather than starting from scratch to simplify and save the time for the refinement process.

#### Generate Ground Truth Masks:

initiates by identifying and correcting boundary-touching errors, zeroing out pixels where segmented labels overlap. This step is critical to ensure that each pixel retains a unique identifier. Furthermore, the function includes a filtering mechanism to manage manually segmented masks sharing identical IDs, maintaining only the largest connected components to ensure each bundle has a distinct ID. This methodical approach is vital for preparing precise ground truth data necessary for effective training processes. Finally, all the masks are saved in a folder pre-defined in the configuration.

#### Display Stored Ground Masks:

allows users to review the stored masks following the automated refinement. This step ensures that all bundles are correctly identified with IDs

#### Move Ground Truth to Training Folder:

automates the transfer of all samples into a pre-configured folder designated for storing training data. This feature is designed to eliminate the need for manual copy-paste operations.

#### Check Training Data:

performs a comprehensive verification of the training data. It ensures that each raw image is paired with a corresponding ground truth and confirms the existence of ‘Train’ and ‘Val’ folders within the configured directories, each containing distinct files. Additionally, this function uploads all masks to verify their uniqueness by checking for unique IDs. Should any issues be detected, the plugin will alert the user with a notification of the problem. Conversely, if all checks are passed successfully, the function will display a congratulatory prompt, asking the user to proceed with the training process.

#### Train New Model for 3DCilia Seg:

initiates the training process for the segmentation algorithm. This button activates an executable via the Windows Subsystem for Linux (WSL) to start training since our segmentation algorithm is designed to operate on a Linux-based system. After the training is complete, the resultant model is stored in a pre-configured location and is ready for use through the plugin. Users have the flexibility to train various models and select the most effective one by simply modifying a path in the configuration file. This plugin is open-source, enabling any user with Python expertise to extend its functionality to suit more specific research needs. Currently, certain tasks such as selecting the region of interest in the Z-dimension and adjusting the rotation are performed manually. However, future versions will include automation of these tasks, with updates made available through our GitHub repository to continuously enhancing the plugin’s capabilities.

## 3 Methods

### Description of microscopy datasets

C57BL/6J mouse P5 and P21 cochleae were harvested and post-fixed overnight at 4°C in 4% PFA. After fixation, cochleae were dissected and tissues were permeabilized in PBS containing 0.3% Triton-X (PBST) for 30 minutes at room temperature. Alexa Fluor 568-conjugated phalloidin was applied in 0.03% PBST containing 3% NGS and incubated for 30 minutes at 23°C. Samples were washed three times with 0.03% PBST for 10 minutes each, mounted in ProLong Glass Antifade (ThermoFisher Scientific, Cat#P36980, Carlsbad, CA, USA) with a #1.5 coverslip, and imaged with a 2.5 *μ*W 561nm laser and a 63× 1.4NA DIC M27 objective on an 880 Airyscan confocal microscope with a 43 × 43nm xy pixel size, 0.110nm z-step size, and a pixel dwell time of 2.3 *μ*s per pixel then processed with default Airyscan processing settings.

Human cochlear tissues were prepared and imaged as described in the original manuscript^37^ underwent a freeze/thaw step in 30% sucrose to permeabilize them, followed by 1 hour at room temperature in a blocking buffer (PBS with 5% normal horse serum and 0.3–1% Triton X-100). The tissue was then incubated overnight at 37°C with some combination of the following primary antibodies in PBS with 1% normal horse serum and 0.3–1% Triton X: (1) rabbit anti-Myosin VI and/or VIIa (Proteus Biosciences #25–6791 and 25–6790, respectively), at 1:100 to count hair cells, and (2) Espin (Sigma #HPA028674) at 1:100 to identify stereocilia. Primary incubations were followed by two sequential 60-minute incubations at 37°C in species-appropriate secondary antibodies (coupled to AlexaFluor dyes) in PBS with 1% normal horse serum and 0.3–1% Triton X. After immunostaining, all pieces from each cochlea were slide-mounted in Vectashield, coverslipped, and the coverslip was sealed with nail polish. For stereocilia analysis, confocal z-stacks were acquired with 0.27 to 0.32 *μ*m z-spacing on a Leica SP8 using a 63x glycerol objective (1.3 NA) with a 120 *μ*m field of view (roughly 13 IHCs and 39 OHCs) and comprising 2664 × 2664 pixels in x and y (16-bit depth per channel). Oversaturation of the image was avoided during acquisition: the maximum saturation across all the images in this study is 60%. At each location, the hair bundles were imaged from the cuticular plate to the stereocilia tips. The image was then deconvolved using the “Lightning” package on the Leica SP8.

### 3.1 3D manual annotation and dataset partitioning

Training a 3D supervised model that efficiently segments each stereocilia bundle requires manual 3D annotation for many bundles, a process that is both cumbersome and slow. We utilized the Computer Vision Annotation Tool (CVAT)^34^ to annotate our 3D samples. CVAT facilitates the drawing of manual polygons with an effective tracking feature, which annotators can use to accelerate the annotation process. We manually annotated 45 stacks using the CVAT cloud application^34^, assigning each 3D bundle a unique ID for precise identification. The annotated data were thoroughly inspected and refined by both the author and the biologists responsible for imaging the data.

To maintain the integrity of the data split, we divided the dataset into training, testing, and validation sets at the stack level, thus preventing the mingling of frames from different stacks during partitioning. The training set comprises 30 stacks, the validation set includes 5 stacks, and the testing set consists of 10 stacks, which are further classified into 6 typical complexity cases and 4 complex cases. The details on how many training, validation, and testing 3D instances exist in this dataset can be found in Table 4.

### 3.2 3D segmentation of stereocilia bundles using 2D detection and multi-object assignment algorithm

Our approach to the 3D segmentation task involves applying 2D detection to each frame, followed by the reconstruction of the 3D object using a multi-object assignment algorithm. We employ the Detectron2 library from Facebook Research^38^, using the Mask R-CNN architecture^39^ combined with a ResNet50 backbone^40^ and a Feature Pyramid Network^41^. This setup leverages transfer learning from a model trained on the COCO dataset^42^. The algorithm is executed over 50,000 iterations with a learning rate of 0.00025. We focus on a single class, specifically the stereocilia bundle, with a head threshold score set at 0.5.

After getting all the 2D frame segmentation masks across all stacks, the multi-object assignment algorithm involve these steps, see Fig 3:

1. Initialization:
  - Set <monosapc>frame_count</monosapc> to zero, marking the start of the frame sequence.
  - Create an empty list <monosapc>tracks</monosapc> to maintain records of active object tracks.
  - Load the initial frame of the stack to start the tracking process.
2. Processing the First Frame:
  - Detect all objects within the first frame and assign each a unique track ID.
  - Store the position and ID of each detected object in <monosapc>tracks</monosapc>.
  - Increment <monosapc>frame_count</monosapc>.
3. Tracking in Subsequent Frames:
  - Load the next frame to continue tracking.
  - Detect all visible objects in the current frame.
  - For each detected object:
    - Calculate the overlap area with each object in <monosapc>tracks</monosapc>.
    - Determine the appropriate track based on overlap:
      - If no significant overlap is found, initialize a new track.
      - If an overlap exists, assign the object to the track with the largest overlap and update the track’s position.
    - Handle ambiguous cases:
      - If multiple objects overlap significantly with a single track, choose the object with the largest overlap for the track.
      - Consider initiating new tracks for other overlapping objects.
  - Increment <monosapc>frame_count</monosapc>.
4. Loop Through Remaining Frames:
  - Repeat the process in Step 3 for each new frame until the end of the stack sequence.
5. Finalization:
  - Assemble and output all completed tracks for further analysis.
  - Conclude the tracking algorithm. At this stage, each cell or bundle is assigned a unique ID based on the tracks to enable the user to visualize them in Napari.

### 3.3 Utilizing pre-trained ResNet50 for targeted classification of cochlear tonotopic regions

In this study, we employed a modified ResNet50 model^40^ using the PyTorch framework^43^ to classify images of cochlear regions into three categories: BASE, MIDDLE, and APEX, which correspond to the high, middle, and low frequency response tonotopic positions of the cochlear spiral. The model, initialized with weights from pre-trained networks, was adapted to our specific task by altering the final fully connected layer to output three classes. When using a pre-trained ResNet50 model, the weights of the model have been adjusted based on its training on ImageNet^44^, contains over 14 million images categorized into over 20,000 classes, where it has likely learned rich feature representations for a wide variety of images. This pre-training makes the model a strong starting point for most visual recognition tasks. To enhance model robustness and adaptability, training involved dynamic augmentation techniques including random resizing, cropping, flipping, color adjustments, and rotations, followed by normalization tailored to the ResNet50 architecture. This approach utilized both frozen and trainable layers, allowing for effective feature extraction adapted from pre-trained domain knowledge while refining the model to the specific needs of our dataset. Training was conducted over 100 epochs with real-time monitoring via TensorBoard, optimizing for accuracy through stochastic gradient descent with momentum. The best performing model was systematically saved to achieve marked improvements in classification accuracy.

### 3.4 IHC and OHC row classification

We implemented a deep learning strategy employing multi-class classification to precisely identify and categorize each hair cell bundle as either IHCs (first row from the buttom), OHC1 (second row from the buttom), OHC2 (third row from the buttom), and OHC3 (forth row from the buttom).

For the training and validation process, we utilized the same dataset used for the 3D segmentation. Each dataset was manually annotated into four rows: IHC, OHC1, OHC2, and OHC3. We employed a multi-class segmentation approach using a U-Net architecture with a ResNet-50 backbone and a feature pyramid network. Transfer learning was utilized, leveraging pre-trained ImageNet weights to enhance model robustness. Given the existing 3D segmentation, our objective was simply to identify which cell corresponds to which row. To this end, we applied maximum projection to all frames in the stack to simplify and standardize the results.

**Figure 11.**
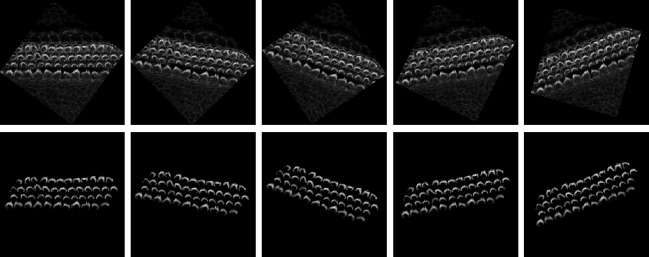
Augmentation of maximum projection images from 3D confocal stacks for training a classification model that discriminates between IHC, OHC1, OHC2, and OHC3.

**Figure 12.**
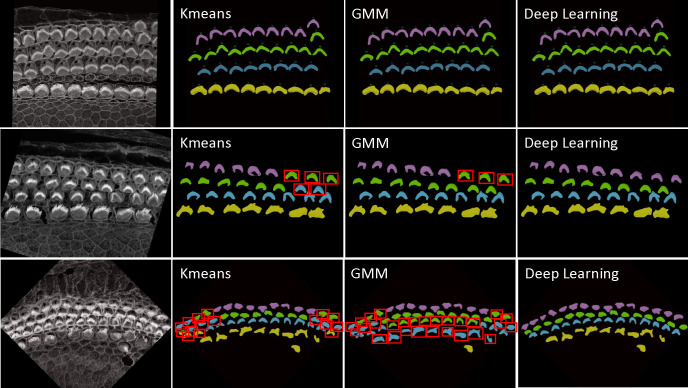
First row: Successful cases for all methods—KMeans, GMM, and Deep Learning—in accurately clustering the four rows into their respective categories are clearly demonstrated. This scenario represents an ideal case where each row is well-separated, linearly aligned, and free from outliers that simplifying the task of accurate clustering. Second and third rows: Failure cases for KMeans and GMM in accurately clustering the four rows into their respective categories are evident: IHC1 in yellow, OHC1 in cyan, OHC2 in green, and OHC3 in magenta. These traditional methods often struggle to precisely segregate the rows due to their inherent limitations in handling complex data distributions, outliers, and overlapping clusters. In contrast, Deep Learning significantly outperforms both KMeans and GMM, providing accurate and reliable clustering for all cell types. Errors are represented by red bounding boxes. For the sample in the second row, there are Five errors in Kmeans, three errors in GMM, and no errors with deep learning. For the sample in the third row, there are Sixteen errors in Kmeans, twenty eight errors in GMM, and no errors with deep learning

**Figure 13.**
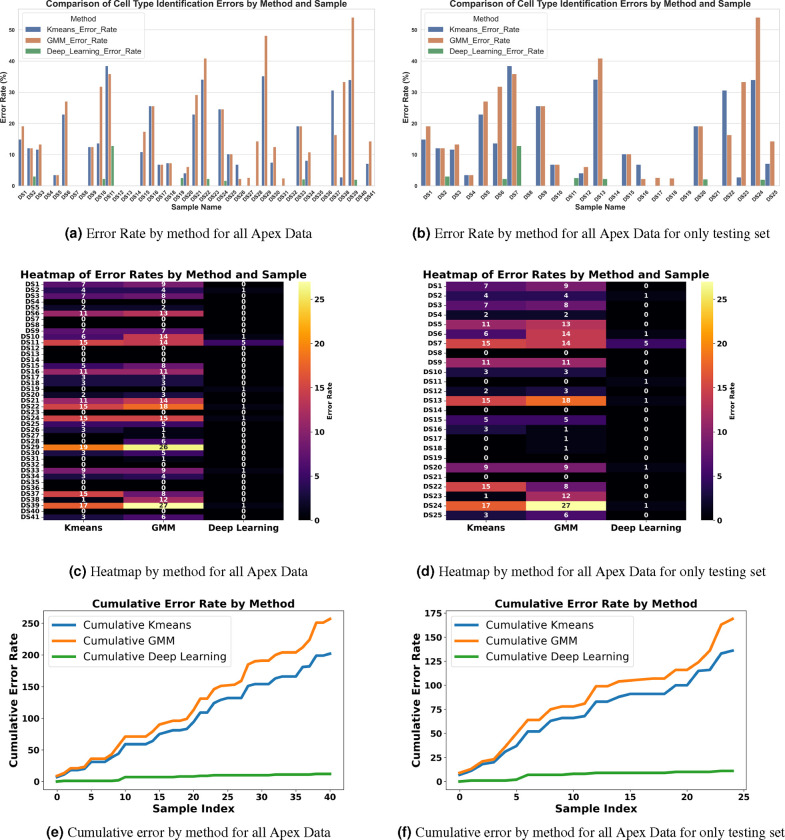
Comprehensive Examination of Error Rates, Heatmaps, and Cumulative Errors for Cell Type Identification in the Apex Region. Subplots (A, B, E, and F) illustrate error rate and cumulative errors, with blue representing KMeans, brown for GMM, and green denoting Deep Learning. Subplots (C and D) utilize the ‘inferno’ colormap to depict error rates, transitioning from black (low errors) to yellow (high errors), providing a visual gradient of error severity. This color-coded representation aids in distinguishing the methodologies applied across different datasets and highlights the specific error dynamics associated with each method.

**Figure 14.**
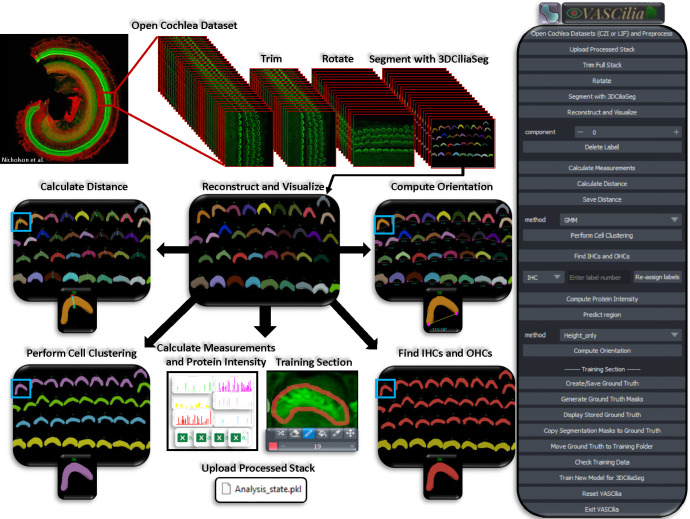
VASCilia enables the ear research community to process their cochlea samples through an end-to-end workflow, all within a user-friendly interface. All images in the workflow are our generated images, except for the top left cochlea image^36^.

Data augmentation has played a crucial role in enhancing the model’s ability to generalize across different laboratory datasets, see Fig 11. Specifically, we have rotated the images by 10 and 20 degrees to both the right and left. Additionally, we have employed segmentation masks to mask out the raw images, effectively eliminating the background. This step creates additional training samples that focus on the foreground, helping the model to generalize better to other datasets where stains may not highlight the background, ensuring more accurate segmentation in diverse experimental conditions. Additionally, a CSV file that records distances is updated to include the new classifications, ensuring that each cell’s identity (whether IHC, OHC1, OHC2, or OHC3) is documented.

### 3.5 Toward developing a foundational model for cochlea research: Integrating diverse data sources

For the final model available to the ear research community, we trained the architecture using a comprehensive dataset. This dataset included all the data from P5 (young mice), which consists of 45 stacks, as well as 10 stacks from P21 (adult mice), and 22 stacks provided by the Liberman lab. This amounted to a total of 901 2D images and 29,963 instances.

Given that this model is trained on young and adult mice of the same strain, as well as data from a different lab with varying settings, stains, and microscopy techniques (our dataset is in .czi format, while theirs is in .lif format), we consider this model to be the first trial in developing a foundational model. It is hoped that this model will work with data from other labs without requiring fine-tuning.

We are committed to maintaining our GitHub repository and actively encourage collaborators from other labs to share their data. By doing so, we aim to broaden the model’s applicability and enhance its robustness, ultimately benefiting the entire ear research community.

### 3.6 Computational resources for all the experiments

All experiments were conducted on a local desktop computer equipped with a 13th Gen Intel(R) Core(TM) i9–13900K CPU, 128GB of RAM, and an NVIDIA GeForce RTX 4080 GPU with 16.0 GB of dedicated memory, running Microsoft Windows 11 Pro. We utilized the PyTorch framework^43^ to implement all machine learning models to develop the Napari plugin. For training using the Detectron2 library from Facebook Research^38^, we utilized the Windows Subsystem for Linux (WSL), as the library is not supported natively on Windows. To integrate the algorithm into the Napari plugin^12^, we built an executable that runs through one of the plugin’s buttons via WSL. Consequently, users will need to set up WSL to utilize this plugin, details of which are thoroughly described in our GitHub documentation.

### 3.7 Data and Code Availability

To support open science and enhance reproducibility, we are pleased to release the annotated dataset comprising 45 stacks used for training in our experiments. This dataset is available for research ear community public use to facilitate further research, result comparison and development in the field.

Additionally, the complete source code for Napari plugin, has been made publicly accessible. The code can be accessed through our dedicated GitHub repository at the following URL: https://github.com/ucsdmanorlab/Napari-VASCilia

We encourage the community to utilize these resources in their research and to contribute improvements or variations to the methodologies presented.

## Figures and Tables

**Table 1.** Measurements from VASCilia and Observers with Descriptive Statistics

Sample Name	ID	VASCilia	Observer1	Observer2

DataSet1	2	3.394	3.513	3.431
	19	2.697	2.675	2.883
	25	3.897	3.590	3.947
	32	3.580	3.367	3.613
DataSet2	10	3.466	3.474	3.466
	32	3.353	2.633	2.454
	24	3.385	3.534	3.575
	31	3.698	3.389	3.159
DataSet3	3	1.884	2.189	2.646
	39	2.078	1.897	2.426
	34	2.223	2.202	2.006
	46	4.440	4.543	4.313
	33	3.774	3.624	2.944
	48	3.450	3.519	3.194
DataSet4	3	2.483	2.446	2.916
	47	2.673	2.706	3.206
	17	2.680	2.628	2.044
	54	2.983	2.559	1.965

**Statistics**		**Mean**	**Median**	**Std. Dev.**
Automated		3.119	3.369	0.673
Observer 1		3.027	3.037	0.663
Observer 2		3.010	3.052	0.649

**Table 2.** Dataset crops that are associated with the computations used in Table 1

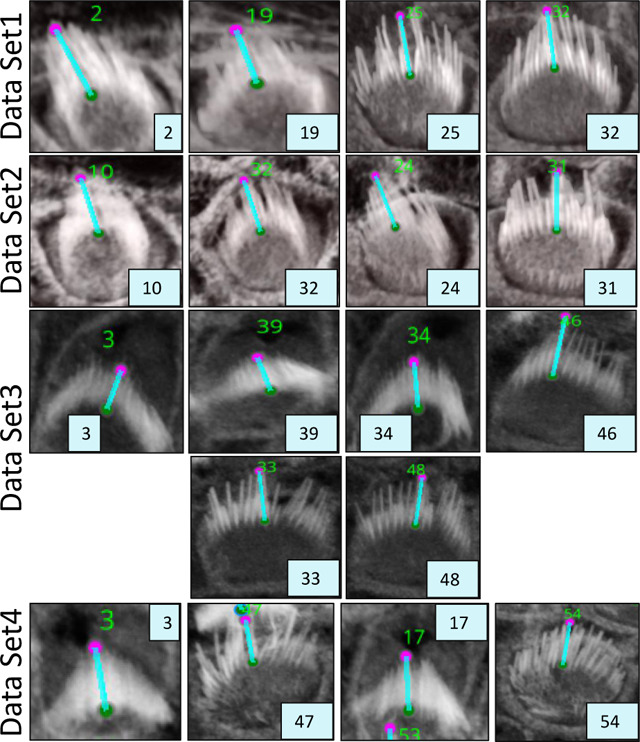

**Table 3.** Pearson Correlation Coefficients, p-values, and Paired t-test Results

Comparison	Correlation Coefficient	Correlation p-value	t-test Statistic	t-test p-value

Automated vs Observer 1	0.939	< 0.001	1.613	0.125
Automated vs Observer 2	0.721	< 0.001	0.904	0.379
Observer 1 vs Observer 2	0.830	< 0.001	0.180	0.860

**Table 4.** Summary of 3D stereocilia bundle instances across different data sets.

Data Set	IHC	OHC	Total
Training (30 stacks)	270	940	1210
Validation (5 stacks)	47	170	217
Inference (10 stacks)	93	350	443
**Total (45 stacks)**	410	1460	1870
