# Supplementary material for: VASCilia (Vision Analysis StereoCilia): A Napari Plugin for Deep Learning-Based 3D Analysis of Cochlear Hair Cell Stereocilia Bundles
Source: bioRxiv. 2025 Sep 23:2024.06.17.599381. Originally published 2024 Jun 17. Preprint. [Version 3] doi: 10.1101/2024.06.17.599381 (PMC11212889; doi:10.1101/2024.06.17.599381)
Supplement: Supplement 1 [file media-1.pdf]

Supplementary Materials

3.13 VASCilia Workflow and Features

VASCilia begins by initializing all necessary properties for comprehensive analysis, invoking a function called 'initialize_ui' to set up all plugin buttons and prepare the user interface for operation.

Users can either open and preprocess a new dataset using the 'Open Cochlea Datasets and Preprocess' button or upload an analyzed dataset with the 'Upload Processed Stack' button. The plugin supports Zeiss (.czi), Leica (.lif), and Tagged Image (.tif) file formats, with the flexibility to incorporate additional formats. It starts by reading metadata and extracting physical resolution variables for later use in length computations, applies Contrast Limited Adaptive Histogram Equalization for pre-processing, and displays the channels as layers in Napari.

Users initiate their analysis by automatically trimming the stack to isolate the CCZ region of interest, aligning the stack with the tissue's planar polarity axis, and then proceeding with segmentation, reconstruction, and visualization. Subsequently, users can remove unwanted regions, perform measurements, calculate lengths from top to bottom of stereocilia bundles, compute fluorescence Intensity, predict the origin of the stack, determine orientation, and cluster rows into four categories (IHC, OHC1, OHC2, OHC3). All these functionalities depends upon the invoke a function called 'save_attributes,' which efficiently saves all variables used in the analysis in a pickle file. This storage action is implicit, requiring no manual save operations from the user, thus enabling the upload function to retrieve and apply these variables for visualization in the viewer. This setup enables analysts to resume or revisit their analysis at a later time.

After analyzing several datasets, users can compile all generated CSV files related to length computations, orientation, and fluorescence signal responses to analyze and plot observations necessary for their studies.

The user can reset Napari for analyzing a new dataset using the 'Reset' button. This feature saves all current variables and then clears them along with all existing layers. This process ensures that the plugin is thoroughly prepared and optimized to handle a new dataset to allow smooth transition between tasks.

3.14 User-Enhanced Accuracy in Automated Measurements

In VASCilia, aside from the segmentation tasks, all automated measurements can be fine-tuned interactively by the user. The plugin is equipped with listeners that actively monitor user interactions related to the adjustment of points, that affect length and orientation computation. This feature ensures that any automated measurements can be further modified to reflect the precise requirements of the user.

Furthermore, for clustering task (cell type identification), VASCilia empowers users to intervene when automated clustering may not align perfectly with the expected outcomes. Users have the flexibility to reassign elements between clusters, correcting any discrepancies. This capability allows for significant refinement of the clustering results to ensure that the automated process is complemented by user expertise and judgment.

3.15 Training section

VASCilia is fundamentally designed to obtain 3D segmentation of stereocilia bundles, an essential step for all subsequent measurements within the plugin. To ensure adaptability and utility across various labs, VASCilia includes a feature allowing users to fine-tune the existing segmentation model with additional images from their specific datasets. This adaptability is crucial for handling variations in staining techniques, settings, and image dimensions such as height, width, and resolution. This feature is both user-friendly and vital for broadening the plugin's applicability. The training module within VASCilia features seven buttons for ease of use:

Create/Save Ground Truth: allows users to generate a new layer, named 'Ground Truth', within the plugin. This button also enables saving of manually annotated data directly within this layer.

Copy Segmentation Masks to Ground Truth: simplifies the annotation process by transferring existing segmented 3D masks to the Ground Truth layer. This functionality allows users to make precise adjustments to the model's initial predictions rather than starting from scratch to simplify and save the time for the refinement process.

Generate Ground Truth Masks: initiates by identifying and correcting boundary-touching errors, zeroing out pixels where segmented labels overlap. This step is critical to ensure that each pixel retains a unique ID. Furthermore, the function includes a filtering mechanism to manage manually segmented masks sharing identical IDs, maintaining only the largest connected components to ensure each bundle has a distinct ID. This methodical approach is vital for preparing precise ground truth data necessary for effective training processes. Finally, all the masks are saved in a folder pre-defined in the configuration.

Display Stored Ground Masks: allows users to review the stored masks following the automated refinement. This step ensures that all bundles are correctly identified with IDs

Move Ground Truth to Training Folder: automates the transfer of all samples into a pre-configured folder designated for storing training data. This feature is designed to eliminate the need for manual copy-paste operations.

Check Training Data: performs a comprehensive verification of the training data. It ensures that each raw image is paired with a corresponding ground truth and confirms the existence of 'Train' and 'Val' folders within the configured directories, each containing distinct files. Additionally, this function uploads all masks to verify their uniqueness by checking for unique IDs. Should any issues be detected, the plugin will alert the user with a notification of the problem. Conversely, if all checks are passed successfully, the function will display a congratulatory prompt, asking the user to proceed with the training process.

Train New Model for 3DBundle Seg: initiates the training process for the segmentation algorithm. Users have the flexibility to train various models and select the most effective one by simply modifying a path in the configuration file. This plugin is open-source, enabling any user with Python expertise to extend its functionality to suit more specific research needs.

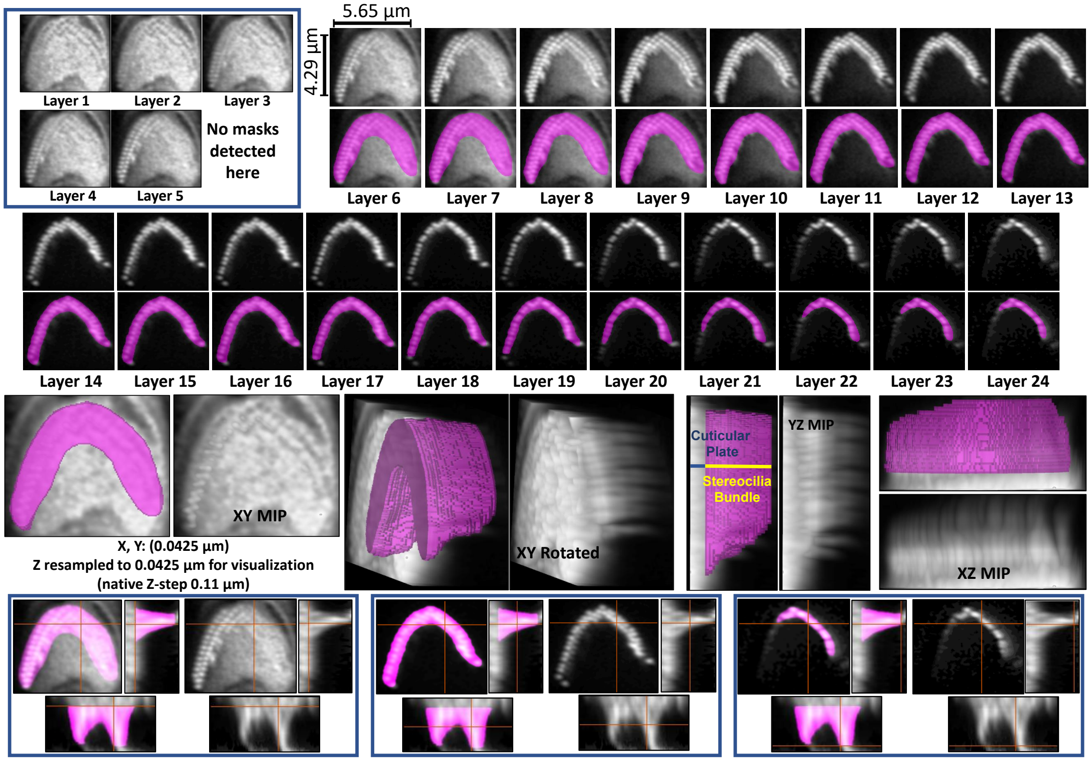

(a) Upright bundle rotated into the *z* plane with strong cuticular-plate signal. Masks follow stereocilia through *z* and stop where the signal disappears; depth is evident in the 3D and YZ views.

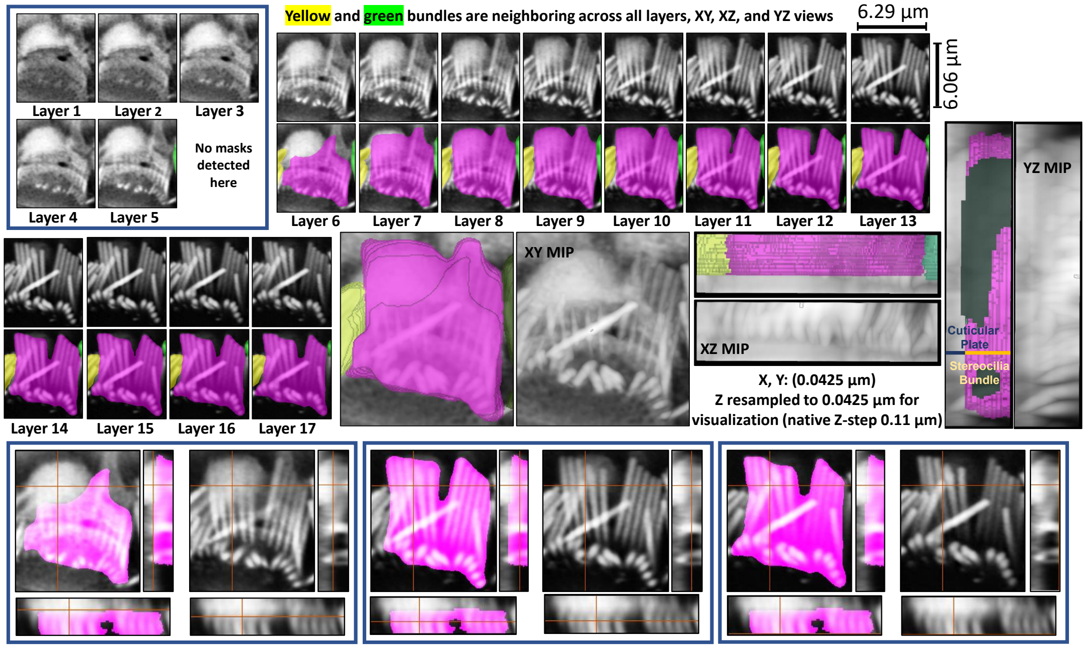

(b) Flattened, overlapping bundle. Masks appear where stereocilia are clearly visible (e.g., layer 6) and are absent in previous layers with only background signal, illustrating selective detection.

Figure 20. Representative P21 Apex stereocilia segmentations for an upright bundle and a flattened bundle, shown across all *z* planes with overlays and 3D views. Segmentation depth follows the stereocilia signal and excludes the cuticular plate unless stereocilia signal is clearly present, showing how the method responds to different depths and orientations.

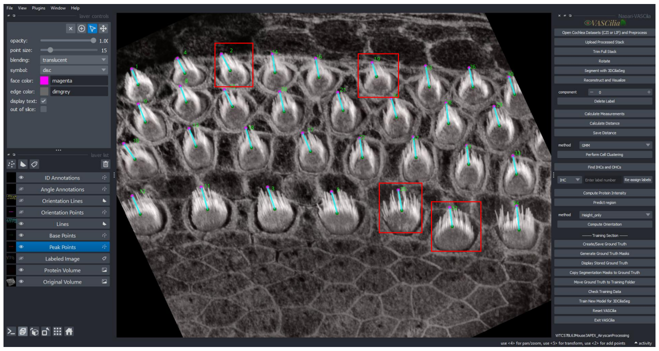

(a) Two IHCs and two OHCs from the apical cochlear turn of a P5 WT mouse

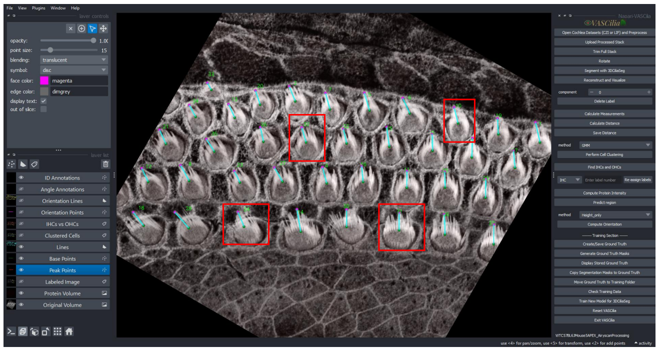

(b) Two IHCs and two OHCs from the apical cochlear turn of a P5 WT mouse

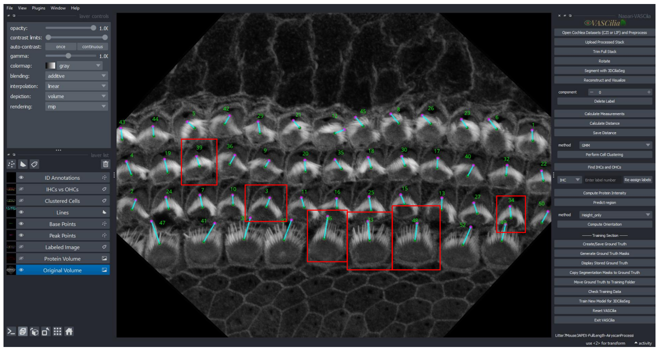

(c) Three IHCs and three OHCs were selected from the apical cochlear turn of P5 *Eps8 KO* mouse injected with an AAV–Anc80L65 vector expressing GFP–*Eps8*.

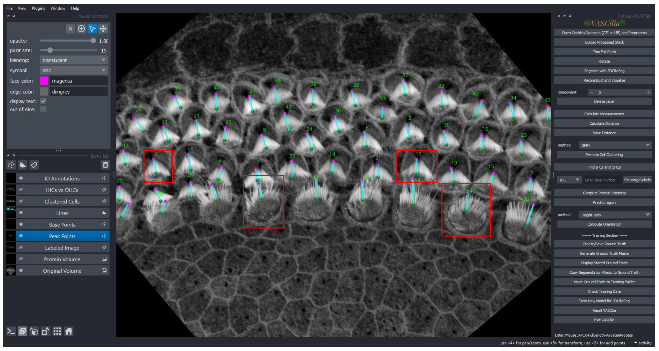

(d) Two IHCs and two OHCs were selected from the apical cochlear turn of P5 *Eps8 KO* mouse injected with an AAV–Anc80L65 vector expressing GFP–*Eps8*

Figure 21. Full *z*-stacks used to derive the measurement crops summarized in Table 4. Each panel shows the apical cochlear turn; red boxes mark the inner hair cells (IHCs) and outer hair cells (OHCs) from which the crops were taken.

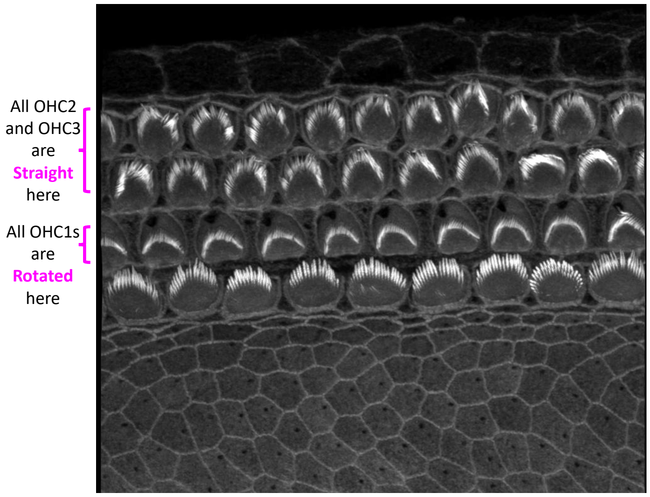

(a) Stack from P5 mouse: OHC2 and OHC3 appear straight; OHC1 is rotated.

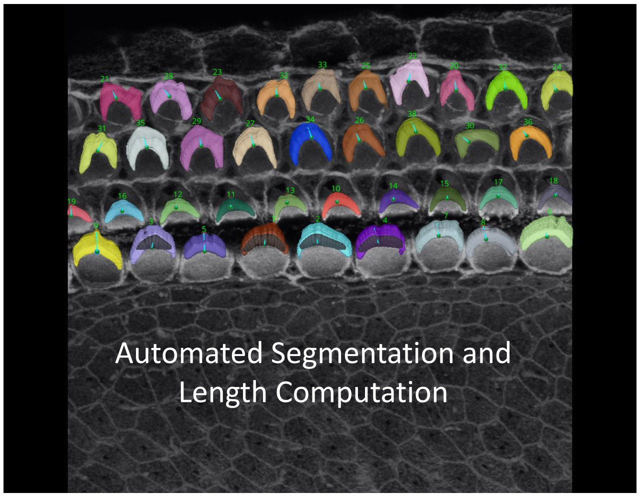

(b) Automatic segmentation for the stack.

ID	Distance	CLass	Depth
1	2.79315	IHC	8
2	2.660582	IHC	9
3	2.955217	IHC	8
4	2.540946	IHC	9
5	2.912053	IHC	8
6	2.619024	IHC	10
7	2.000707	IHC	8
8	2.116468	IHC	6
9	3.076384	IHC	8
10	1.486676	OHC1	10
11	1.586737	OHC1	10
12	1.563191	OHC1	10
13	1.452829	OHC1	10
14	1.550992	OHC1	10
15	1.812385	OHC1	10
16	1.75457	OHC1	10
17	1.65755	OHC1	10
18	1.693337	OHC1	9
19	1.15153	OHC1	5
20	1.939306	OHC3	5
21	1.69437	OHC3	5
22	1.87094	OHC3	5
23	2.089284	OHC3	5
24	1.458561	OHC3	5
25	1.570248	OHC3	5
26	1.678279	OHC2	5
27	1.739153	OHC2	5
28	1.666923	OHC3	5
29	1.653849	OHC2	5
30	1.666923	OHC2	5
31	2.082346	OHC2	5
32	1.554618	OHC3	5
33	1.480717	OHC3	5
34	1.725054	OHC2	5
35	1.881545	OHC2	5
36	1.488029	OHC2	5
37	1.69437	OHC3	5
38	1.716117	OHC2	5

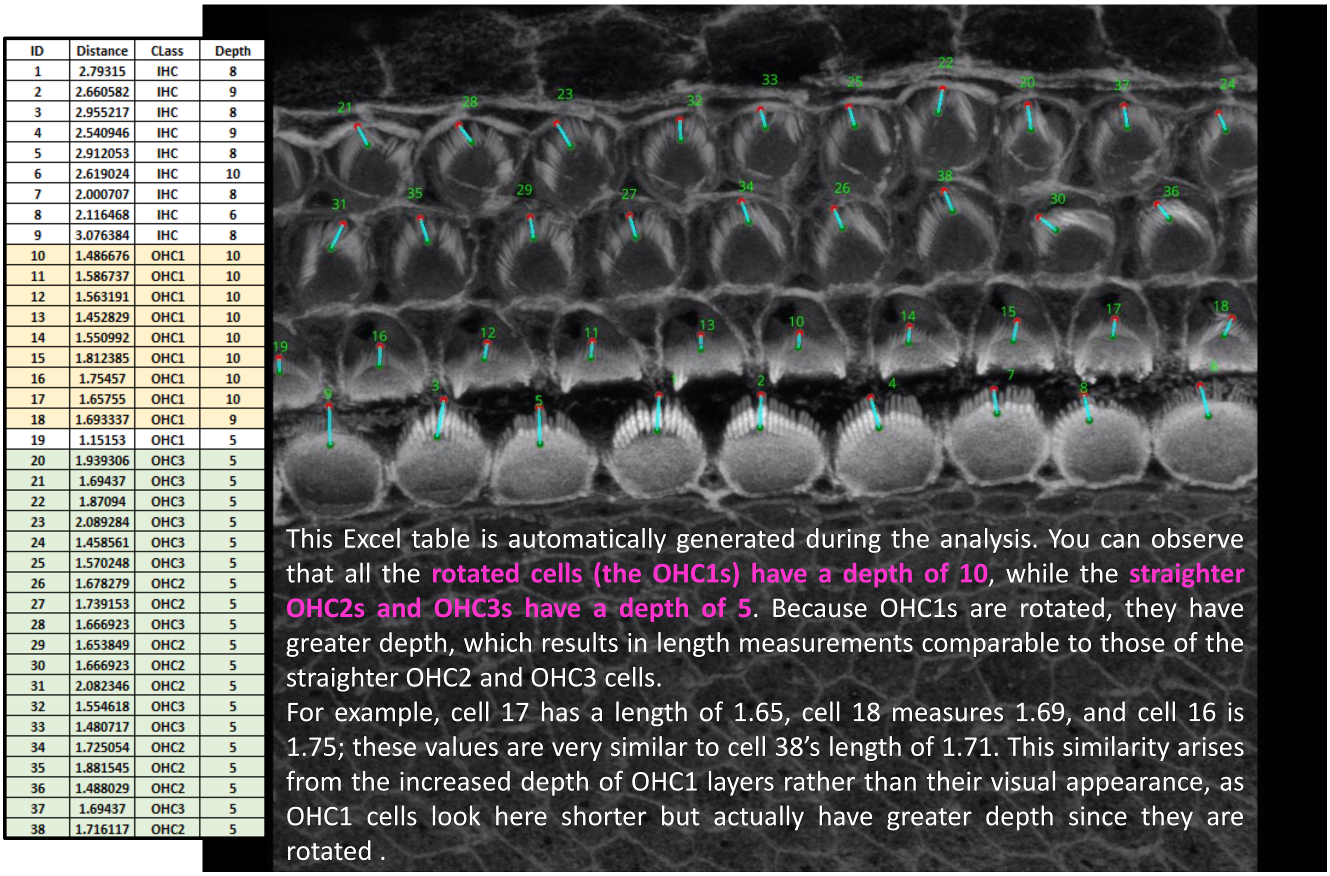

(c) Length computation and measurements.

Figure 22. Straight vs. rotated (P5): raw stack, automatic segmentation, and length readouts. Because measurements are computed in 3D, the plugin captures bundle depth and robustly handles both straight and rotated bundles—capabilities not achievable with 2D measurements.

Tonotopic_KO_WT_Class	mean_height	std_height	median_height	count
WT_Base_IHC	3.03	0.41	2.91	26
WT_Middle_IHC	3.27	0.46	3.24	28
WT_Apex_IHC	3.90	0.55	4.01	27
KO_Base_IHC	1.97	0.33	1.91	26
KO_Middle_IHC	2.07	0.24	2.05	26
KO_Apex_IHC	2.50	0.58	2.37	26
WT_Base_OHC	2.29	0.27	2.32	81
WT_Middle_OHC	2.52	0.40	2.47	85
WT_Apex_OHC	2.94	0.53	2.94	100
KO_Base_OHC	1.46	0.20	1.44	77
KO_Middle_OHC	1.54	0.23	1.51	88
KO_Apex_OHC	1.91	0.38	1.90	98

Table 7. Bundle height summary by genotype (WT/KO), tonotopic region (Base, Middle, Apex), and cell type (IHC/OHC), related to Fig. 9a. Values are in μm; each row reports mean, SD, median, and sample size (N). Row order matches the violin plot. Totals across all groups: IHC $N = 159$, OHC $N = 529$, overall $N = 688$.

KO_WT	Cell	Pair (level1–level2)	n1/n2	mean1	mean2	Δ (95% CI)	g	p_{adj} (Holm)	Status
WT	IHC	Base–Middle	26/28	3.03	3.27	−0.24 (−0.48, −0.01)	−0.56	0.0425	Significant
WT	IHC	Middle–Apex	28/27	3.27	3.90	−0.63 (−0.90, −0.36)	−1.23	5.35×10^{-5}	Significant
WT	IHC	Base–Apex	26/27	3.03	3.90	−0.87 (−1.14, −0.61)	−1.78	8.31×10^{-8}	Significant
WT	OHC	Base–Middle	81/85	2.29	2.52	−0.23 (−0.34, −0.13)	−0.68	1.86×10^{-5}	Significant
WT	OHC	Middle–Apex	85/100	2.52	2.94	−0.42 (−0.55, −0.28)	−0.87	1.42×10^{-8}	Significant
WT	OHC	Base–Apex	81/100	2.29	2.94	−0.65 (−0.77, −0.53)	−1.50	8.70×10^{-20}	Significant
KO	IHC	Base–Middle	26/26	1.97	2.07	−0.10 (−0.26, 0.06)	−0.35	0.209	Not significant
KO	IHC	Middle–Apex	26/26	2.07	2.50	−0.43 (−0.68, −0.18)	−0.95	0.00271	Significant
KO	IHC	Base–Apex	26/26	1.97	2.50	−0.54 (−0.80, −0.27)	−1.11	6.33×10^{-4}	Significant
KO	OHC	Base–Middle	77/88	1.46	1.54	−0.08 (−0.15, −0.01)	−0.37	0.0164	Significant
KO	OHC	Middle–Apex	88/98	1.54	1.91	−0.36 (−0.45, −0.28)	−1.15	3.69×10^{-13}	Significant
KO	OHC	Base–Apex	77/98	1.46	1.91	−0.45 (−0.53, −0.36)	−1.41	8.55×10^{-18}	Significant

Table 8. Pairwise tonotopic contrasts in bundle height by genotype (WT/KO) and cell type (IHC/OHC), related to Fig. 9a. Within each stratum we compare Base–Middle, Middle–Apex, and Base–Apex using Welch two-sided t-tests. Reported are sample sizes (n_1/n_2), group means (mean1/mean2, in μm), the difference $\Delta = \text{mean}_1 - \text{mean}_2$ with 95% CI, Hedges' g, and Holm-adjusted p-values. Negative Δ and g indicate level2 > level1. All contrasts are significant at $p_{\text{adj}} < 0.05$ except KO IHC Base–Middle.

Tonotopic_KO_WT_Class	mean_value	std_value	median_value	count
WT_Base_IHC	0.70	0.27	0.84	26
WT_Middle_IHC	0.24	0.13	0.18	28
WT_Apex_IHC	0.17	0.11	0.15	27
KO_Base_IHC	0.51	0.14	0.55	26
KO_Middle_IHC	0.33	0.09	0.32	26
KO_Apex_IHC	0.29	0.24	0.25	26
WT_Base_OHC	0.42	0.12	0.44	81
WT_Middle_OHC	0.16	0.09	0.13	85
WT_Apex_OHC	0.12	0.08	0.09	100
KO_Base_OHC	0.28	0.11	0.24	77
KO_Middle_OHC	0.26	0.09	0.27	88
KO_Apex_OHC	0.14	0.11	0.11	98

Table 9. Normalized fluorescence intensity (unitless) by genotype (WT/KO), tonotopic region (Base, Middle, Apex), and cell type (IHC/OHC), ordered to match the violin plot (Fig. 9b). Each row reports the mean, SD, median, and sample size (N). Totals: IHC $N = 159$, OHC $N = 529$, overall $N = 688$.

KO_WT	Cell	Pair (level1–level2)	n1/n2	mean1	mean2	Δ (95% CI)	g	p_{adj} (Holm)	Status
WT	IHC	Base–Middle	26/28	0.702	0.241	$+0.461\,(0.342, 0.580)$	2.16	6.27×10^{-9}	Significant
WT	IHC	Middle–Apex	28/27	0.241	0.173	$+0.068\,(0.002, 0.133)$	0.55	0.0429	Significant
WT	IHC	Base–Apex	26/27	0.702	0.173	$+0.528\,(0.411, 0.646)$	2.51	3.98×10^{-10}	Significant
WT	OHC	Base–Middle	81/85	0.419	0.161	$+0.259\,(0.227, 0.290)$	2.53	2.57×10^{-34}	Significant
WT	OHC	Middle–Apex	85/100	0.161	0.123	$+0.038\,(0.013, 0.063)$	0.45	0.00258	Significant
WT	OHC	Base–Apex	81/100	0.419	0.123	$+0.297\,(0.266, 0.327)$	2.99	6.39×10^{-41}	Significant
KO	IHC	Base–Middle	26/26	0.512	0.329	$+0.183\,(0.116, 0.250)$	1.51	6.00×10^{-6}	Significant
KO	IHC	Middle–Apex	26/26	0.329	0.287	$+0.042\,(-0.062, 0.146)$	0.23	0.414	Not significant
KO	IHC	Base–Apex	26/26	0.512	0.287	$+0.225\,(0.113, 0.337)$	1.11	4.34×10^{-4}	Significant
KO	OHC	Base–Middle	77/88	0.277	0.258	$+0.0186\,(-0.0138, 0.0511)$	0.18	0.258	Not significant
KO	OHC	Middle–Apex	88/98	0.258	0.140	$+0.118\,(0.088, 0.148)$	1.12	1.32×10^{-12}	Significant
KO	OHC	Base–Apex	77/98	0.277	0.140	$+0.137\,(0.103, 0.171)$	1.20	1.07×10^{-12}	Significant

Table 10. Pairwise tonotopic contrasts for *normalized intensity* by genotype (WT/KO) and cell type (IHC/OHC). We report sample sizes (n_1/n_2), group means (mean1/mean2), difference $\Delta = \text{mean}_1 - \text{mean}_2$ with 95% CI, Hedges' g, and Holm-adjusted p-values. Positive Δ indicates level1 $>$ level2.

Block 1			Block 2			Block 3		
#	Manual	VASCilia	#	Manual	VASCilia	#	Manual	VASCilia
1	112.88°	111.10°	12	99.06°	95.34°	23	93.13°	91.16°
2	109.81°	110.56°	13	92.32°	95.23°	24	94.97°	94.14°
3	78.61°	76.35°	14	104.65°	102.91°	25	81.81°	81.49°
4	101.31°	95.34°	15	92.07°	92.69°	26	83.60°	87.44°
5	94.44°	97.28°	16	91.53°	87.61°	27	91.46°	90.36°
6	93.99°	93.89°	17	99.88°	99.33°	28	91.01°	90.81°
7	98.62°	94.76°	18	77.42°	75.48°	29	89.46°	89.60°
8	87.31°	88.01°	19	100.13°	97.38°	30	92.29°	92.47°
9	88.23°	90.00°	20	91.15°	89.55°	31	104.42°	102.40°
10	99.05°	98.39°	21	92.70°	91.60°	32	90.53°	88.45°
11	113.96°	113.20°	22	83.06°	86.16°	33	88.57°	90.35°
**Summary —** Mean (Manual/VASCilia): 94.04 / 93.35; Median: 92.31 / 92.47; SD: 8.83 / 8.44								

Table 11. Per-cell orientation angles measured manually in Fiji and compared with VASCilia (related to Fig. 13).

Block1			Block2		
#	Manual	VASCilia	#	Manual	VASCilia
1	103.75°	99.68°	10	91.18°	90.53°
2	87.73°	89.43°	11	85.14°	84.44°
3	88.17°	87.95°	12	89.42°	86.52°
4	72.48°	80.68°	13	80.01°	85.75°
5	103.04°	98.58°	14	94.86°	89.51°
6	79.96°	76.09°	15	89.39°	88.89°
7	84.80°	85.24°	16	82.46°	84.13°
8	85.56°	80.89°	17	91.14°	85.75°
9	85.87°	82.95°			
**Summary —** Mean (Manual/VASCilia): 87.94 / 86.88; Median: 87.73 / 85.75; SD: 7.80 / 5.90					

Table 12. Comparison of orientation angles measured manually in Fiji and by VASCilia, displayed in two horizontal panels (related to Fig. 14).

Block A			Block B			Block C		
#	Manual	VASCilia	#	Manual	VASCilia	#	Manual	VASCilia
1	1.603	1.696	6	2.252	2.179	11	1.989	1.986
2	1.629	1.715	7	2.086	2.009	12	1.703	1.741
3	1.855	1.858	8	2.832	2.816	13	1.313	1.277
4	1.711	1.634	9	2.035	1.965	14	1.635	1.787
5	1.193	1.218	10	2.216	2.249	15	1.333	1.400
					**Mean / SD**	**Mean**	**1.826**	**1.835**
						**Std Dev**	**0.426**	**0.405**
					**Paired t-test p-value**		**0.609**	
					**Wilcoxon signed-rank p-value**		**0.720**	

Table 13. Per-cell length values from human-annotated ground truth for *Eps8* KO mice using Fiji and VASCilia for 15 cells. The comparison showed no statistically significant difference between the two methods. Specifically, the mean stereocilia length was 1.826 µm for Fiji and 1.835 µm for VASCilia, with standard deviations of 0.426 µm and 0.405 µm, respectively. A Wilcoxon signed-rank test and a paired t-test both confirmed the absence of significant differences.

Block A			Block B			Block C		
#	Manual	VASCilia	#	Manual	VASCilia	#	Manual	VASCilia
1	1.964	1.948	6	1.777	1.803	11	1.658	1.684
2	1.873	1.879	7	1.756	1.790	12	1.430	1.248
3	2.139	2.105	8	2.754	2.739	13	1.186	1.082
4	2.260	2.356	9	1.475	1.592	14	0.866	0.886
5	1.735	1.955	10	2.117	1.948	15	1.697	1.751
					**Mean / SD**	**Mean**	**1.779**	**1.784**
						**Std Dev**	**0.455**	**0.468**
					**Paired t-test p-value**		**0.851**	
					**Wilcoxon signed-rank p-value**		**0.639**	

Table 14. Per-cell length values from human-annotated ground truth for $Cdh23^{-/-}$ mice using Fiji and VASCilia for 15 cells. The comparison showed no statistically significant difference between the two methods. Specifically, the mean stereocilia length was 1.779 µm for Fiji and 1.784 µm for VASCilia, with standard deviations of 0.455 µm and 0.468 µm, respectively. A Wilcoxon signed-rank test and a paired t-test both confirmed the absence of significant differences.

Block A			Block B			Block C		
**ID**	**Manual**	**VASCilia**	**ID**	**Manual**	**VASCilia**	**ID**	**Manual**	**VASCilia**
cell8	86.32°	88.17°	cell13	88.32°	90.00°	cell18	101.30°	101.31°
cell7	83.26°	86.68°	cell14	102.46°	96.71°	cell20	104.85°	109.03°
cell24	95.98°	92.49°	cell15	60.09°	68.20°	cell48	140.98°	139.09°
cell6	90.68°	90.00°	cell9	109.07°	103.39°	cell33	140.20°	138.99°
cell5	91.56°	91.61°	cell11	119.81°	117.98°	cell26	107.27°	104.04°
cell4	87.22°	89.26°	cell44	113.11°	112.99°	cell29	122.89°	123.69°
cell1	71.19°	75.23°	cell45	135.39°	131.28°	cell32	77.60°	77.91°
cell3	85.28°	84.35°	cell42	92.95°	92.39°	cell38	29.47°	31.18°
cell2	74.90°	79.00°	cell34	98.97°	96.77°	cell36	80.41°	82.03°
cell39	129.93°	125.54°	cell35	107.80°	103.24°	cell40	126.73°	128.88°
cell27	146.07°	151.19°	cell30	121.94°	121.61°	cell37	117.13°	115.46°
cell31	123.36°	128.99°	cell19	119.58°	124.22°	cell47	64.80°	66.80°
cell28	90.53°	90.00°	cell16	90.43°	88.98°	cell41	116.55°	114.90°
cell25	110.79°	109.44°	cell21	92.18°	92.39°	cell43	122.59°	122.35°
cell22	151.15°	149.66°	cell12	75.11°	78.69°	cell46	107.57°	106.86°
cell17	136.45°	135.00°						
						**Mean**	**103.090°**	**103.222°**
						**Std Dev**	**24.940**	**24.061**
						**Paired t-test p-value**	**0.783**	
						**Wilcoxon signed-rank p-value**	**0.965**	

Table 15. Per-bundle orientation values (degrees) for a PCP-deficit mouse cochlear dataset[41], see Fig. 23, measured in Fiji (manually) and with VASCilia (automated). A paired t-test and a Wilcoxon signed-rank test indicate no significant difference.

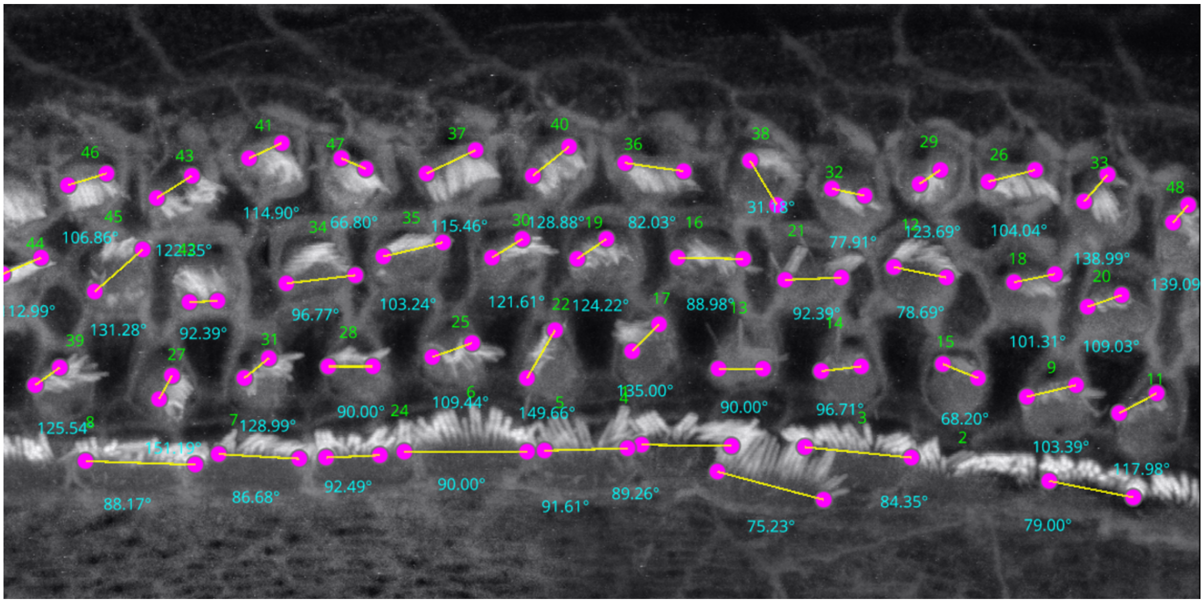

Figure 23. Angle computation on a PCP-deficit cochlear dataset[41]. VASCilia recovers bundle orientation and agrees closely with the manual measurements using Fiji (see Table 15; paired t-test $p = 0.783$, Wilcoxon $p = 0.965$).

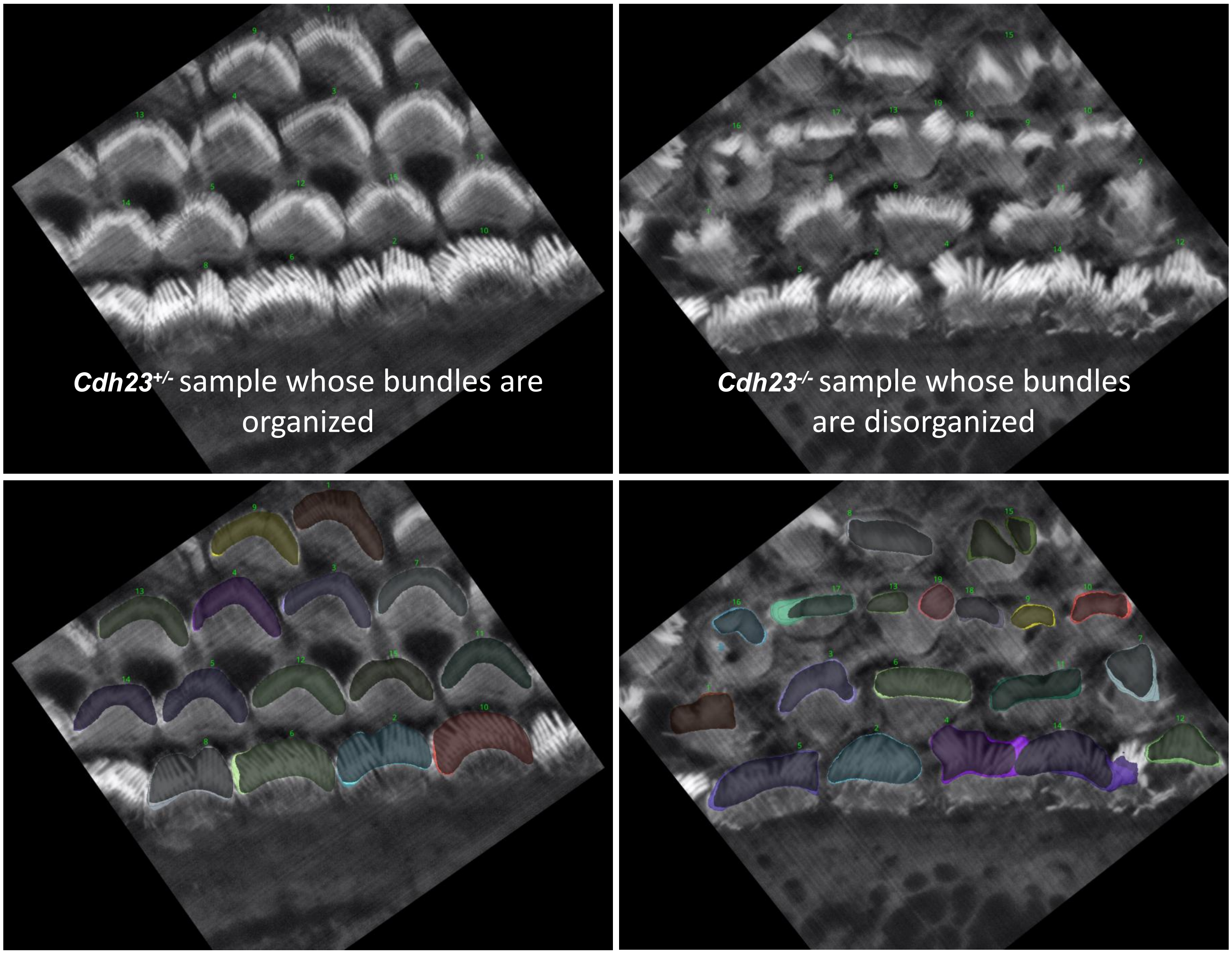

Figure 24. Representative images for $Cdh23^{+/-}$ and $Cdh23^{-/-}$ mouse

VASCilia analysis for Lab A's datasets: screenshots for various samples.

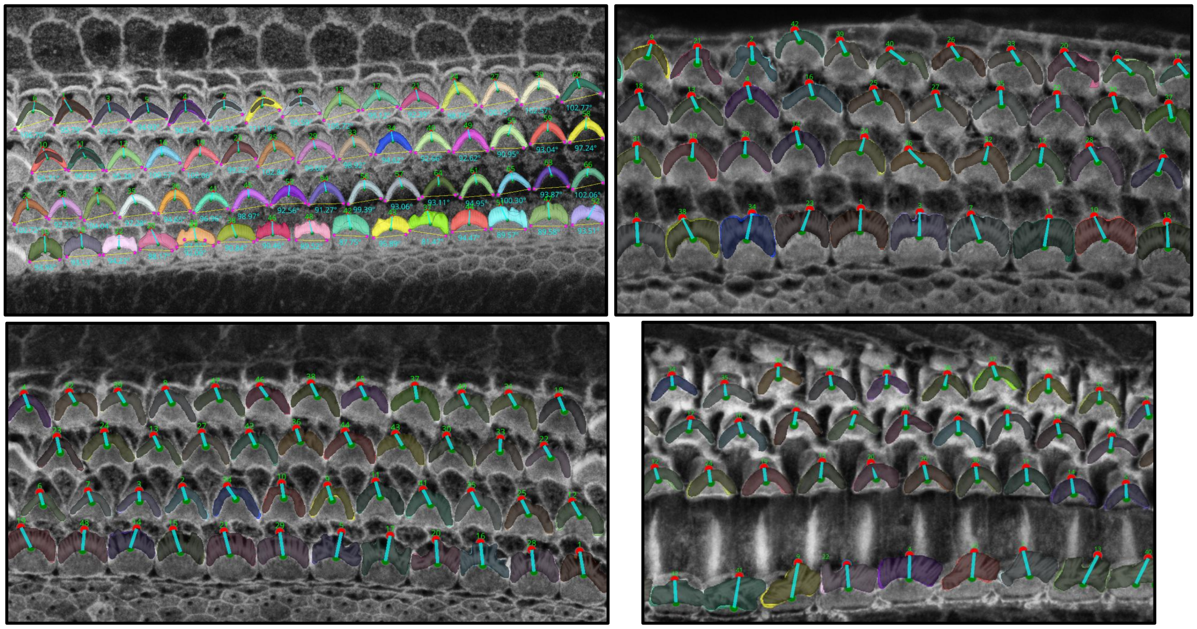

Figure 25. Screenshot of the software with different samples.

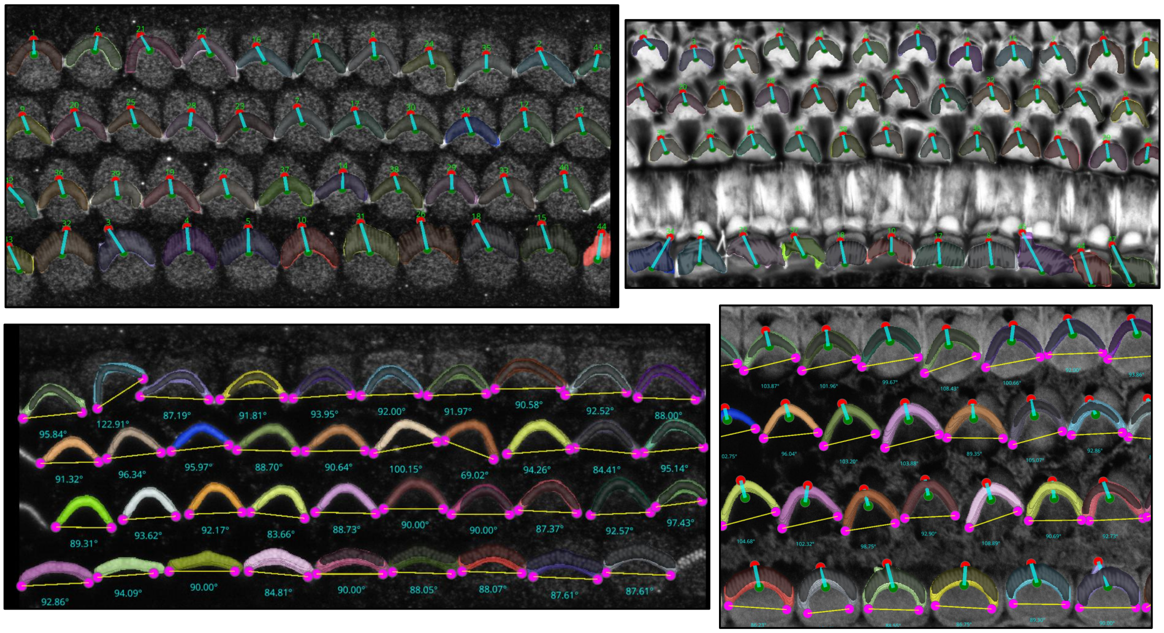

Figure 26. Screenshot of the software with different samples.

VASCilia analysis for Lab B's datasets: screenshots for three samples.

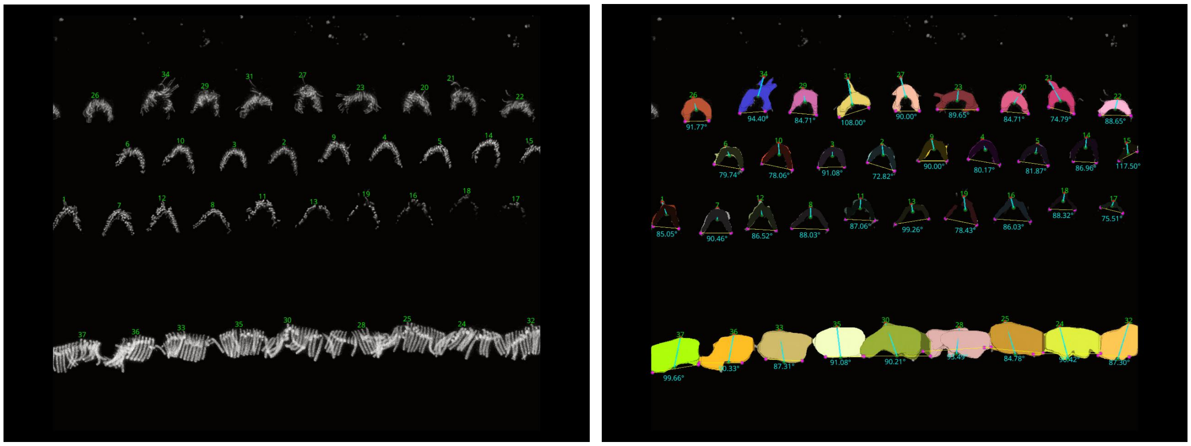

Figure 27. Screenshot of the software with Lab B's mouse cochlea data.

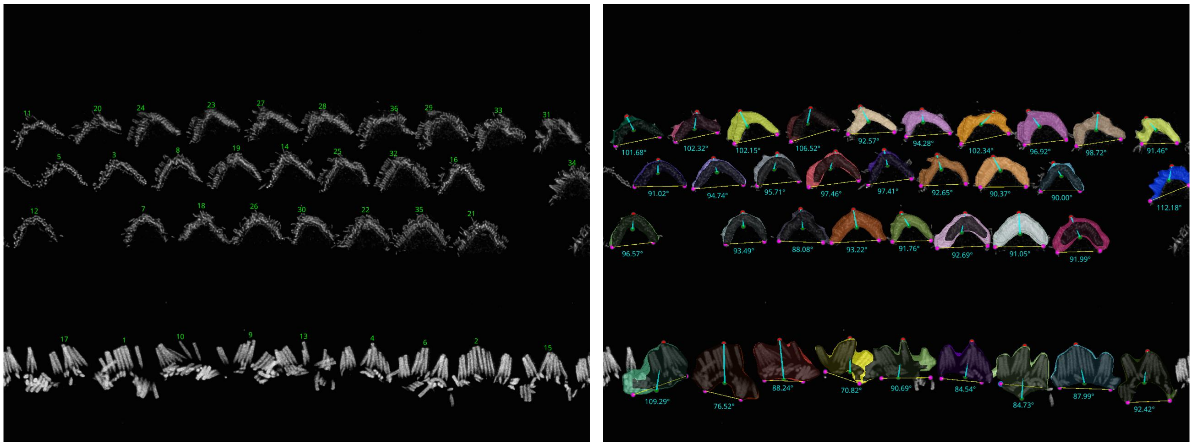

Figure 28. Screenshot of the software with Lab B's mouse cochlea data.

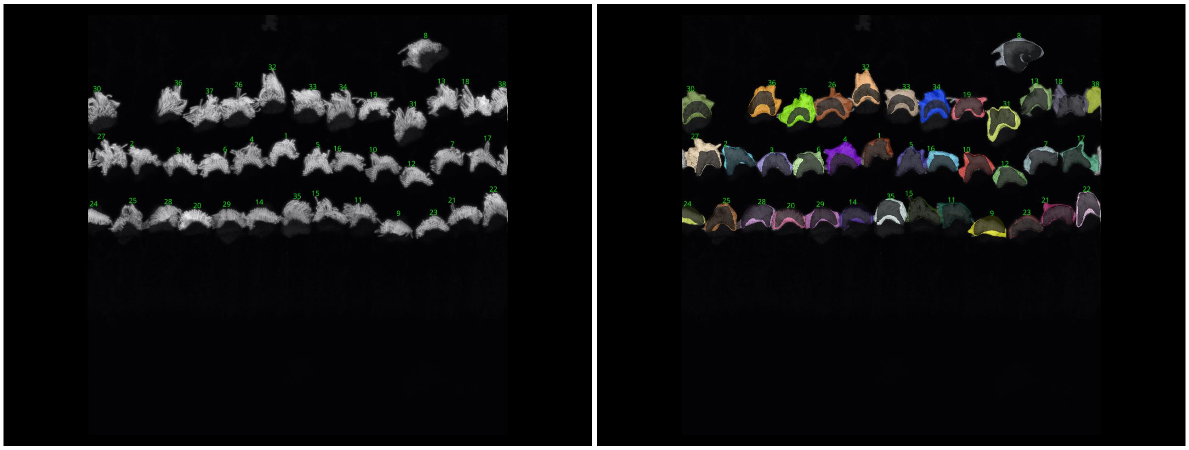

Figure 29. Screenshot of the software with Lab B's human cochlea data.

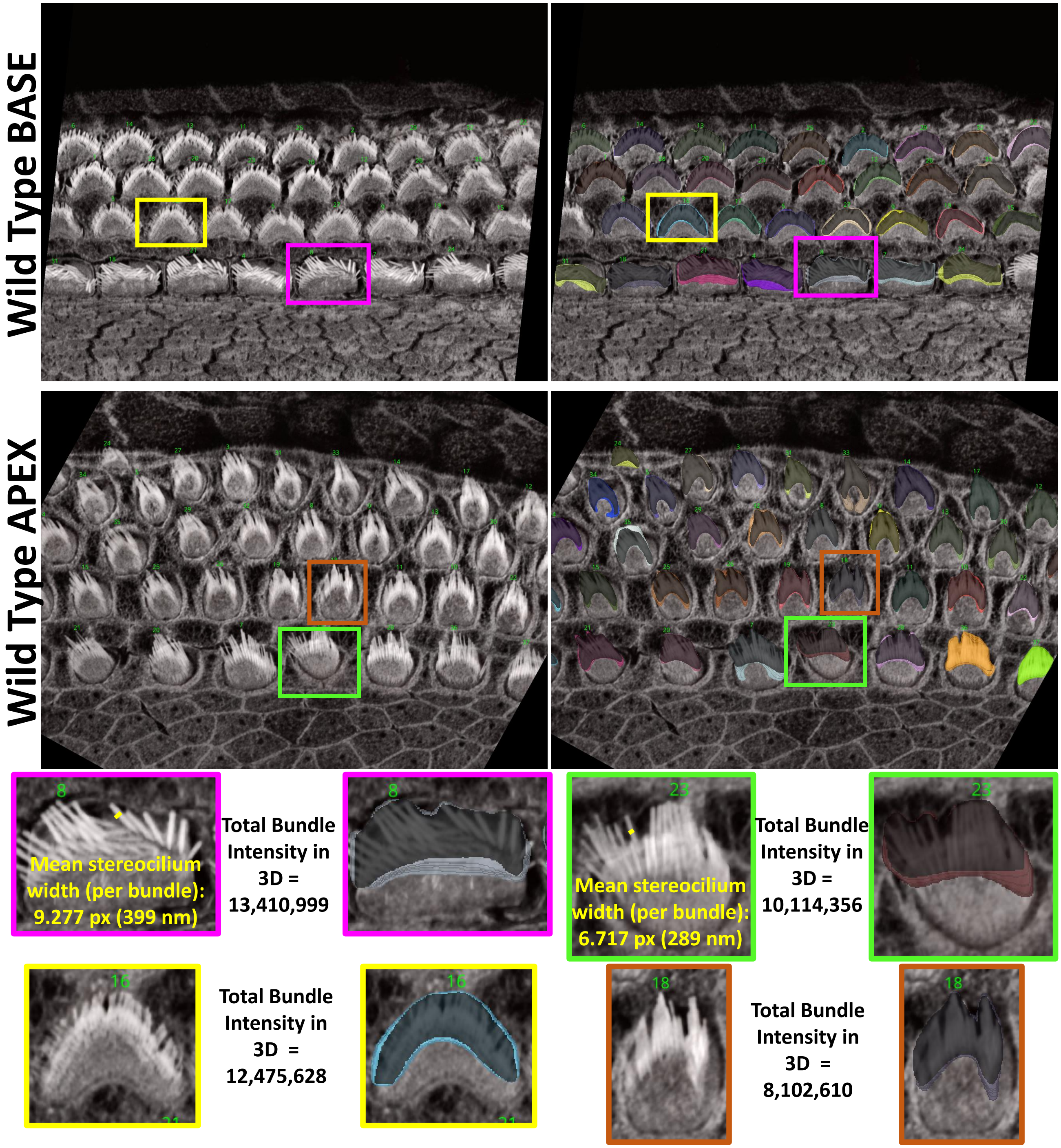

Figure 30. Visual comparison of base and apex bundles in WT animals reveals that the base appears stiffer, with wider stereocilia and bundles, while the apex displays finer stereocilia and tighter bundles, leading to a reduced phalloidin intensity signal.
