# Supplementary material for: VASCilia (Vision Analysis StereoCilia): A Napari Plugin for Deep Learning-Based 3D Analysis of Cochlear Hair Cell Stereocilia Bundles
Source: bioRxiv. 2025 Feb 15:2024.06.17.599381. Originally published 2024 Jun 17. Preprint. [Version 2] doi: 10.1101/2024.06.17.599381 (PMC11212889; doi:10.1101/2024.06.17.599381)
Supplement: Supplementary file 1 [file NIHPP2024.06.17.599381v2-supplement-1.pdf]

bioRxiv preprint doi: https://doi.org/10.1101/2024.06.17.599381; this version posted February 15, 2025. The copyright holder for this preprint (which was not certified by peer review) is the author/funder, who has granted bioRxiv a license to display the preprint in perpetuity. It is made available under aCC-BY 4.0 International license.

Supplementary Materials

VASCilia workflow software plugin screenshots, illustrating all steps and buttons

1. Open Cochlea Datasets (CZI, LIF, TIF) and Preprocess button:

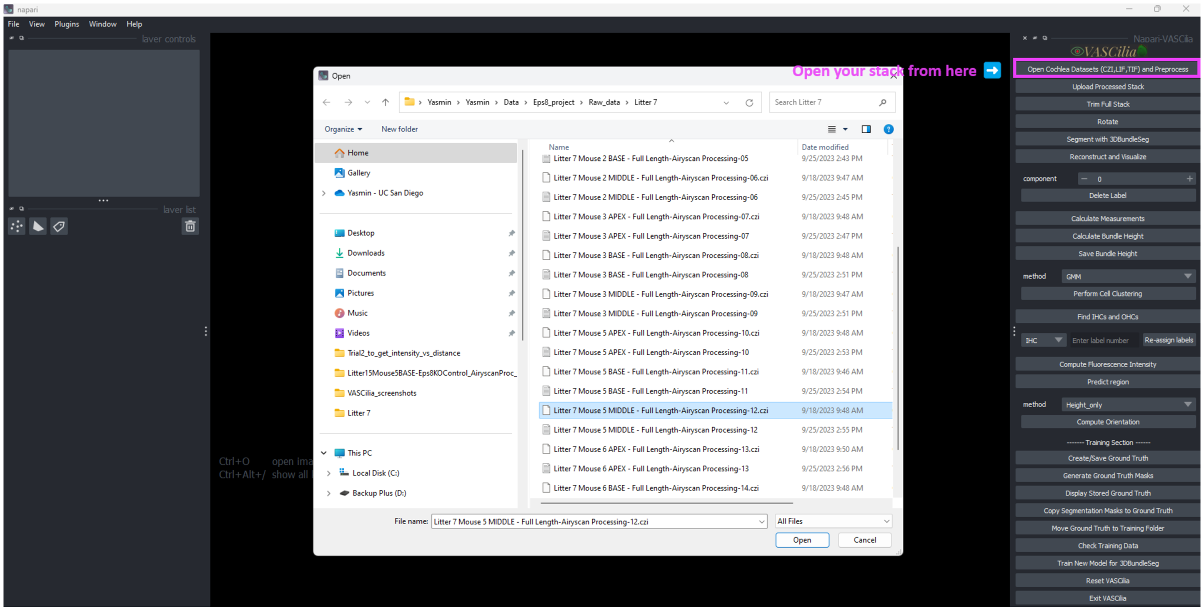

Figure 19. Screenshot of the software's opening screen, showcasing the initial interface and available options. The software supports three formats: CZI, LIF, and TIF.

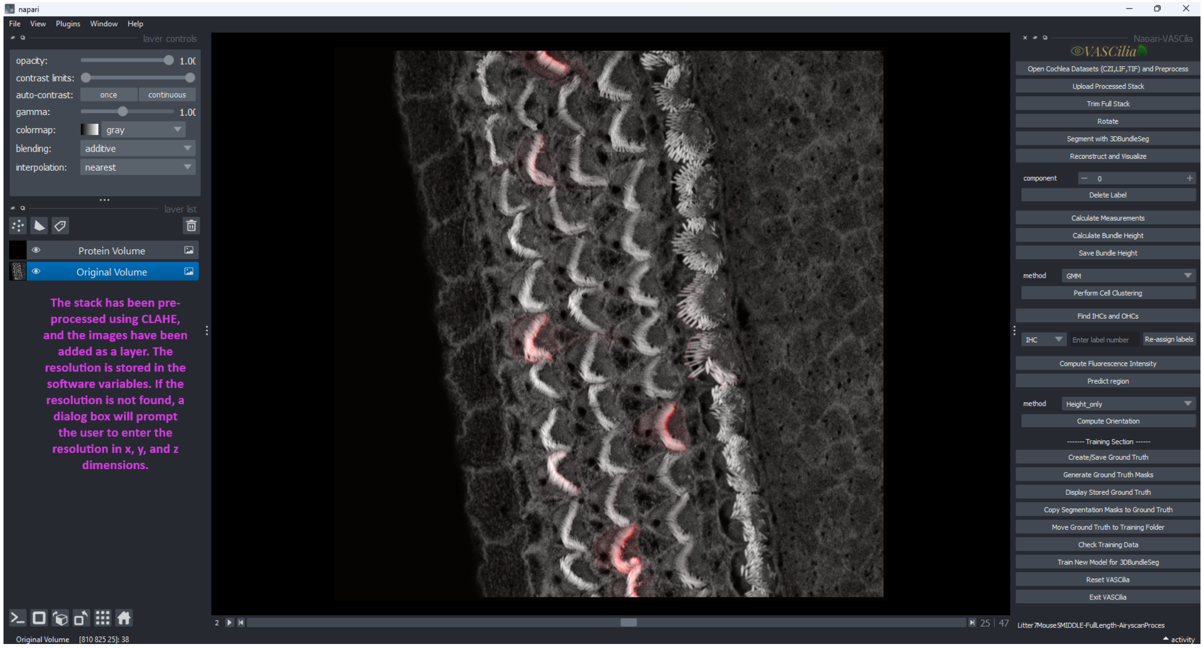

Figure 20. Screenshot of the software's opening screen, displaying the stack as a layer.

bioRxiv preprint doi: https://doi.org/10.1101/2024.06.17.599381; this version posted February 15, 2025. The copyright holder for this preprint (which was not certified by peer review) is the author/funder, who has granted bioRxiv a license to display the preprint in perpetuity. It is made available under aCC-BY 4.0 International license.

2. Upload Processessed stack button:

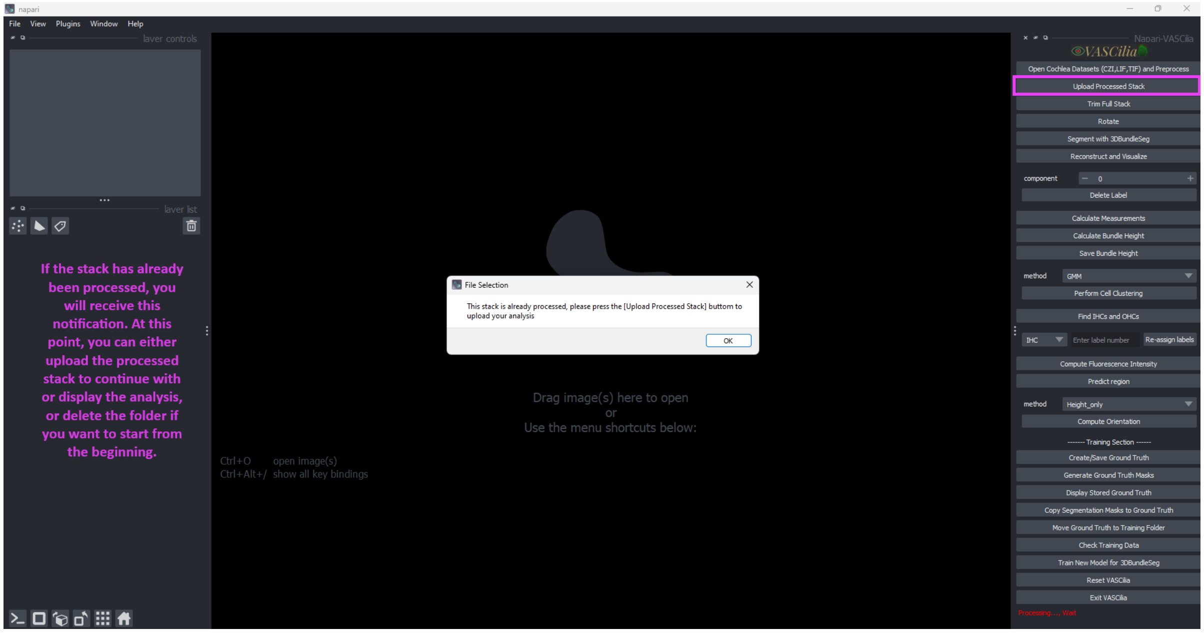

Figure 21. Screenshot of the software's opening screen showing that the stack is already opened and processed.

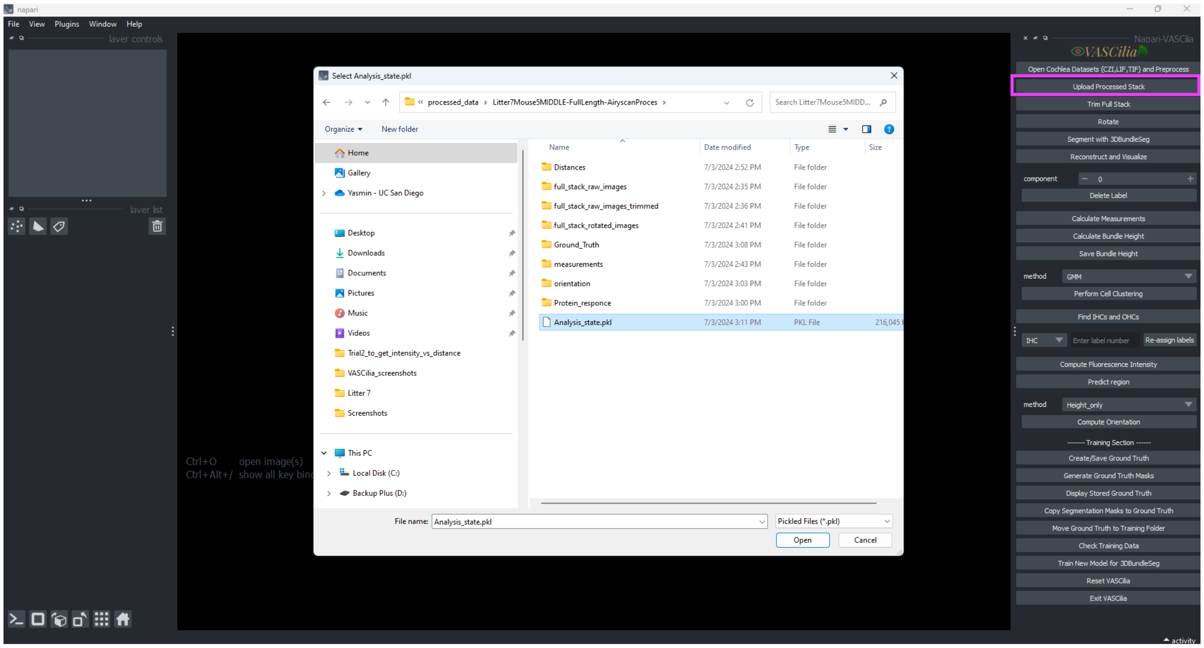

Figure 22. Screenshot of the software's screen showing that the processed stack can be uploaded from the pickle file.

bioRxiv preprint doi: https://doi.org/10.1101/2024.06.17.599381; this version posted February 15, 2025. The copyright holder for this preprint (which was not certified by peer review) is the author/funder, who has granted bioRxiv a license to display the preprint in perpetuity. It is made available under aCC-BY 4.0 International license.

3. Trim Full Stack button:

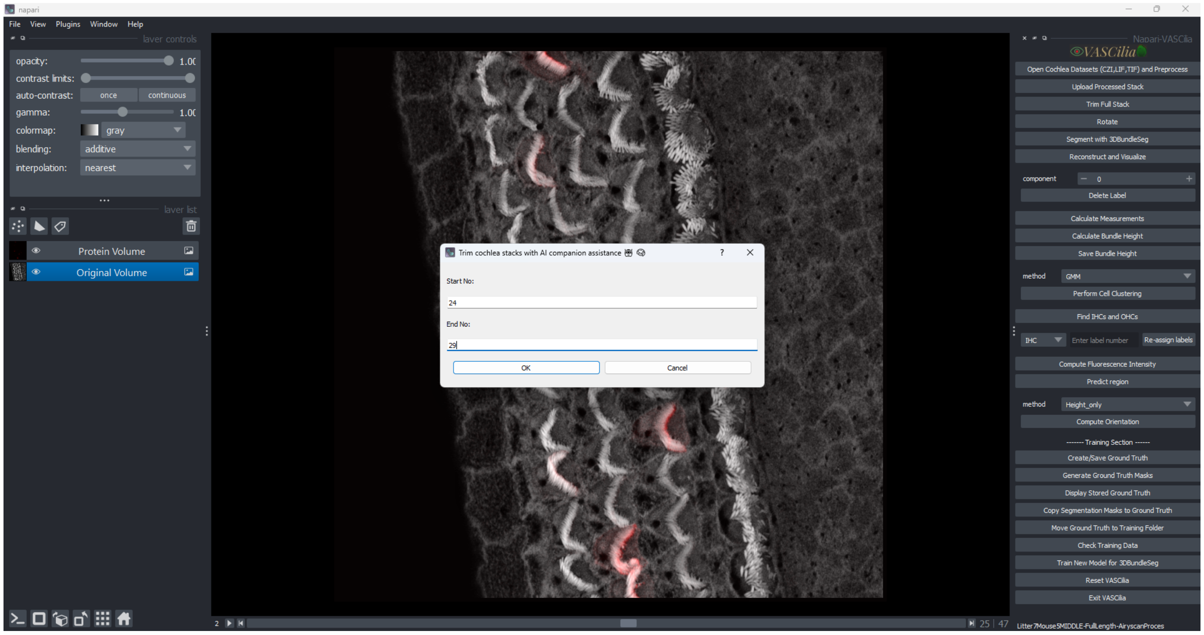

Figure 23. A screenshot of the software's screen demonstrating how the automated model ZFT-Net provides preliminary suggestions for trimming, while still allowing the user to manually select the slices of interest.

4. Rotate button:

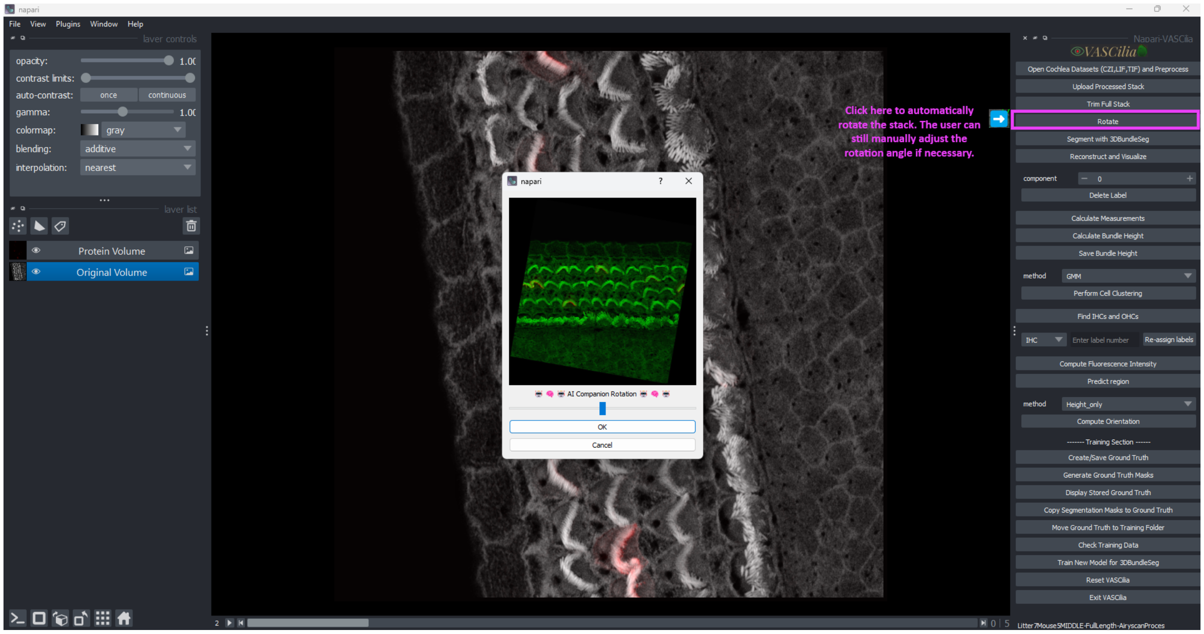

Figure 24. Screenshot of the software's screen for rotating the stack using the trained model, while still allowing the user to manually correct the orientation angle if necessary

bioRxiv preprint doi: https://doi.org/10.1101/2024.06.17.599381; this version posted February 15, 2025. The copyright holder for this preprint (which was not certified by peer review) is the author/funder, who has granted bioRxiv a license to display the preprint in perpetuity. It is made available under aCC-BY 4.0 International license.

5. Segment with 3DBundleSeg button:

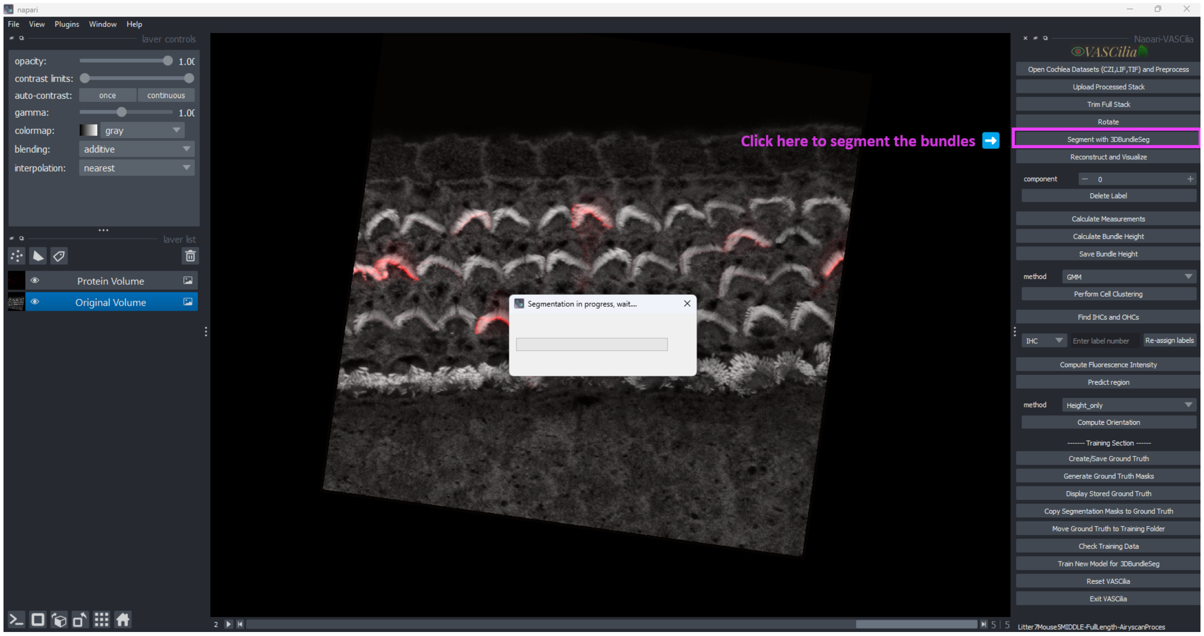

Figure 25. Screenshot of the software's screen for segmenting the stack.

6. Reconstruct and Visualize button:

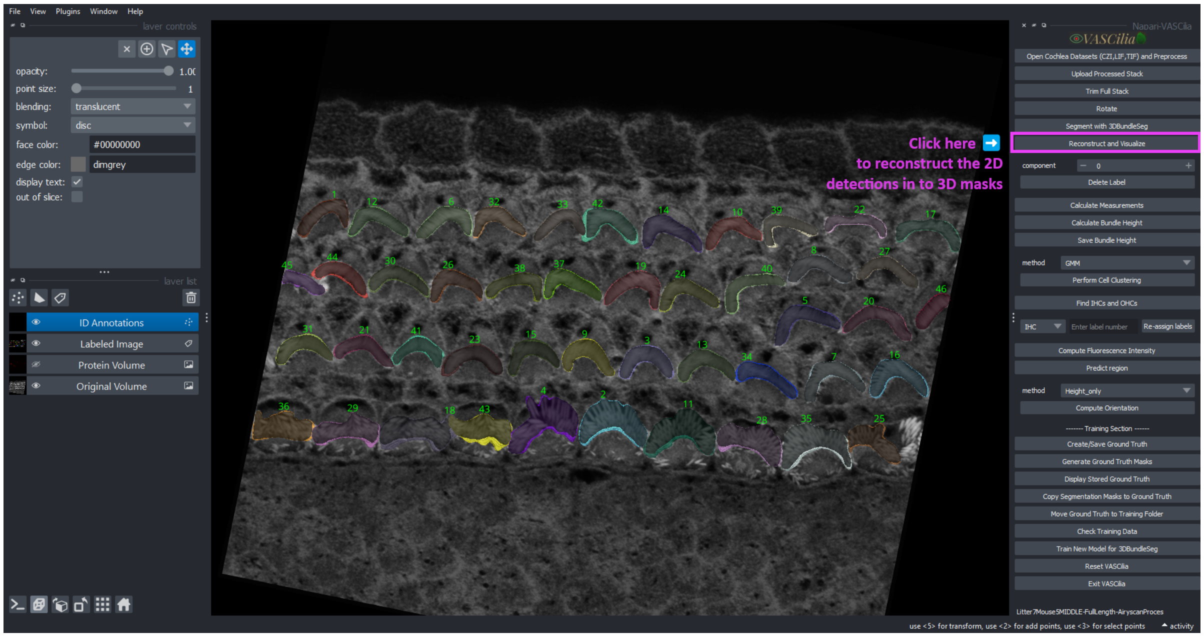

Figure 26. Screenshot of the software showing the bundle segmentation results.

bioRxiv preprint doi: https://doi.org/10.1101/2024.06.17.599381; this version posted February 15, 2025. The copyright holder for this preprint (which was not certified by peer review) is the author/funder, who has granted bioRxiv a license to display the preprint in perpetuity. It is made available under aCC-BY 4.0 International license.

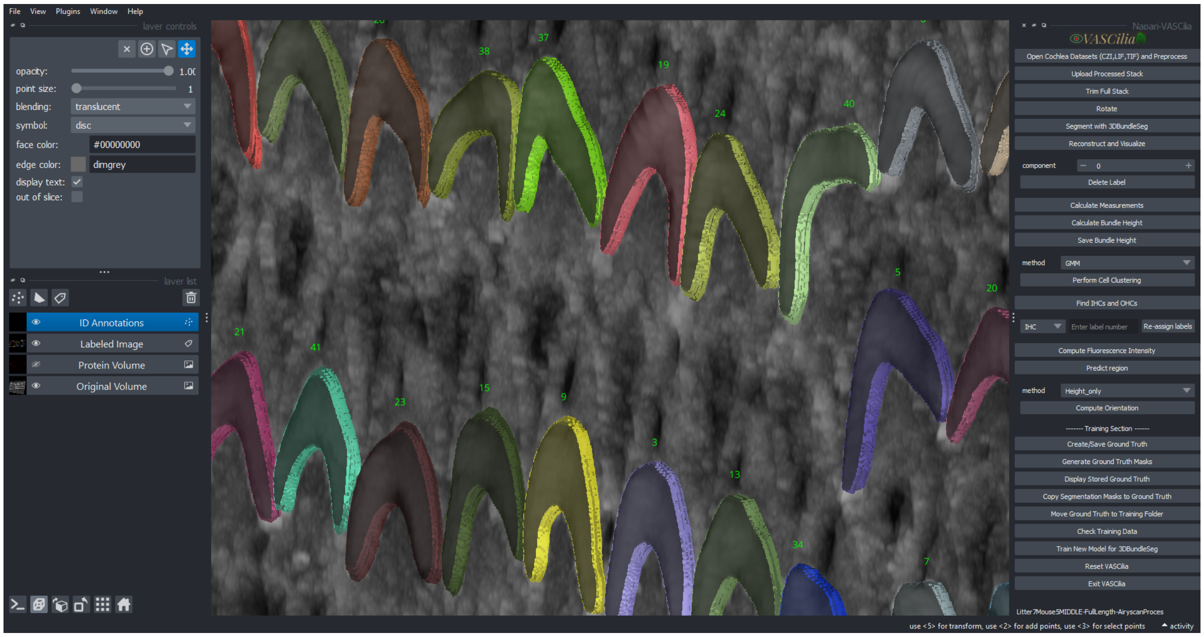

Figure 27. Screenshot of the software with a zoomed-in view of bundle segmentation, showing each segmented bundle labeled with an ID.

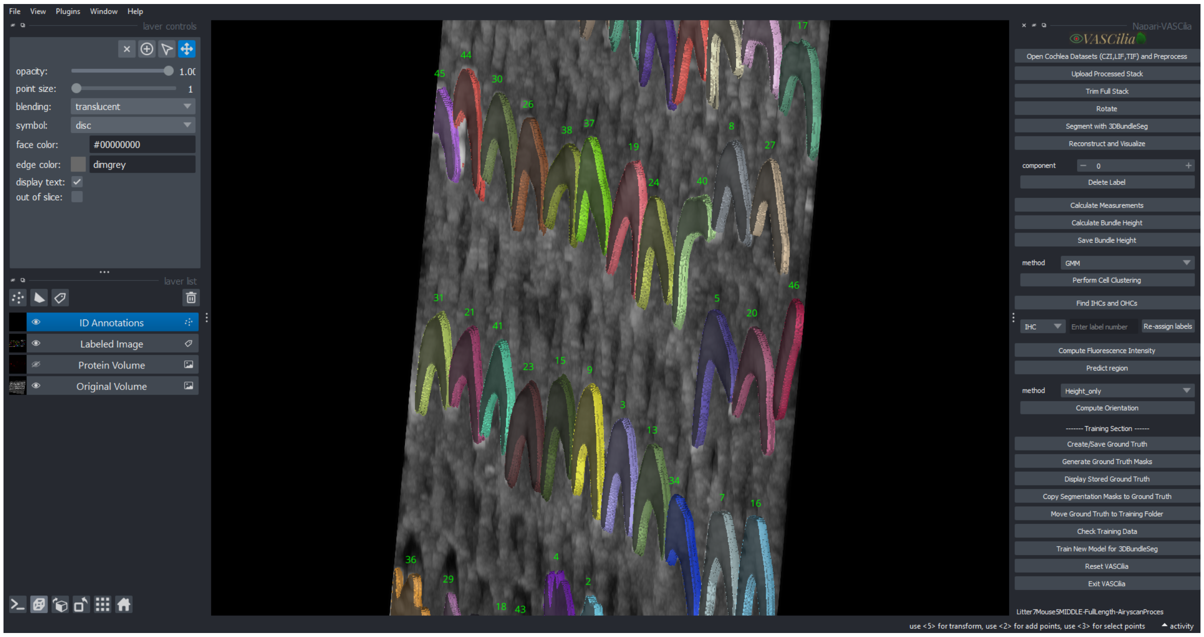

Figure 28. Screenshot of the software with bundle segmentation while the user is rotating the layer.

bioRxiv preprint doi: https://doi.org/10.1101/2024.06.17.599381; this version posted February 15, 2025. The copyright holder for this preprint (which was not certified by peer review) is the author/funder, who has granted bioRxiv a license to display the preprint in perpetuity. It is made available under aCC-BY 4.0 International license.

7. Delete button:

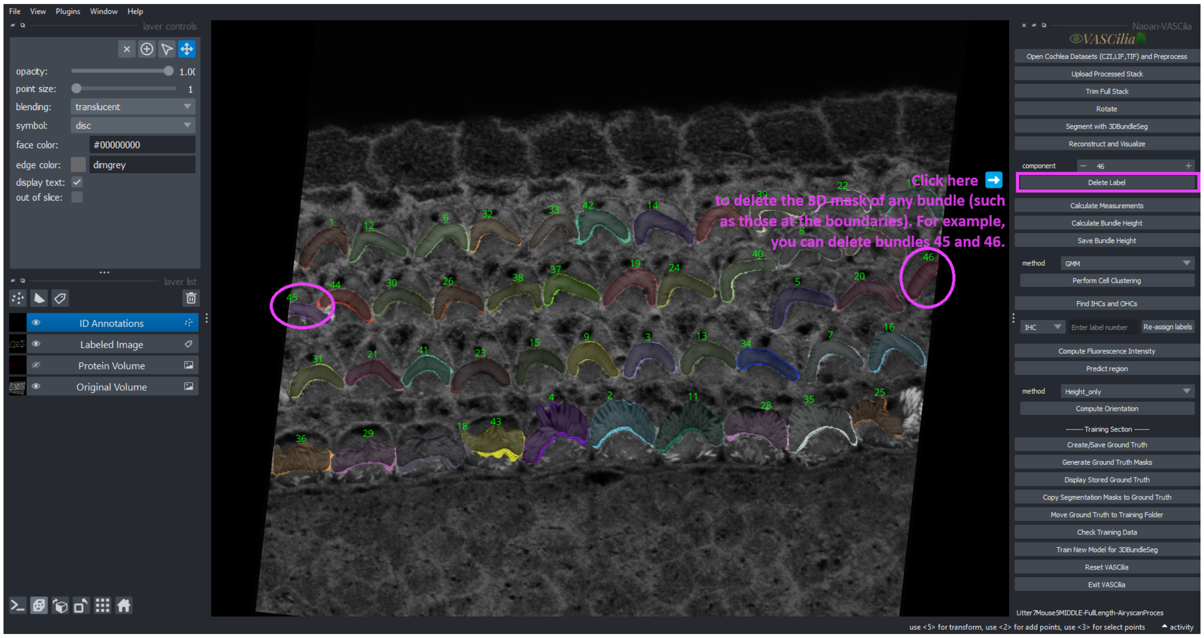

Figure 29. Screenshot of the software with the delete action.

8. Calculate measurements:

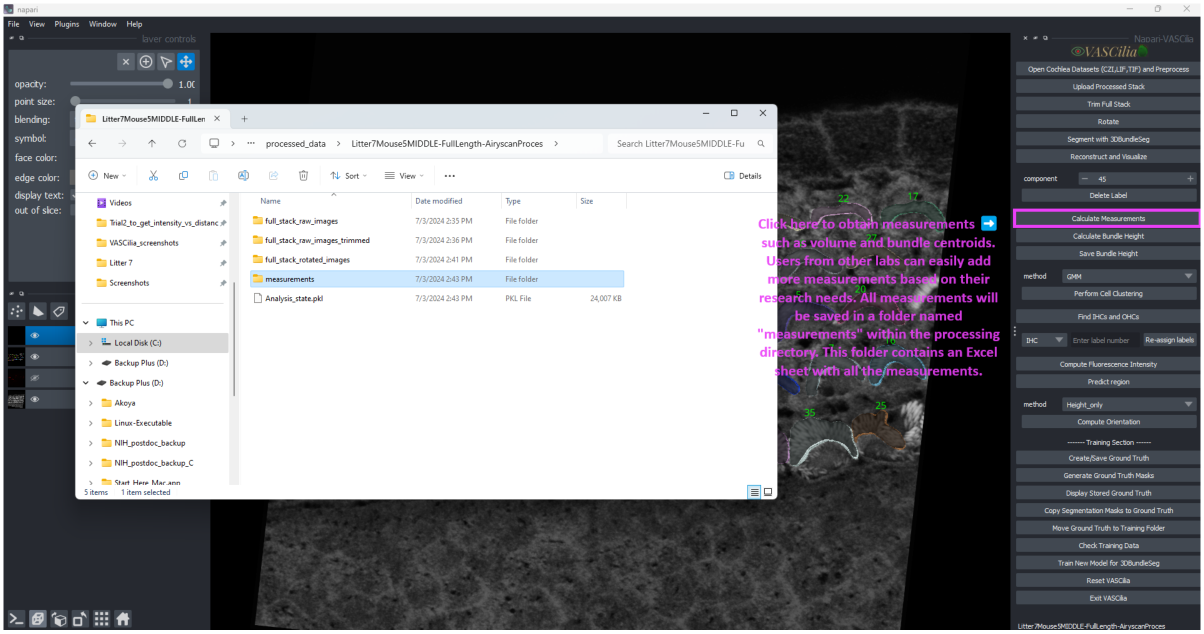

Figure 30. Screenshot of the software with measurements calculation action.

bioRxiv preprint doi: https://doi.org/10.1101/2024.06.17.599381; this version posted February 15, 2025. The copyright holder for this preprint (which was not certified by peer review) is the author/funder, who has granted bioRxiv a license to display the preprint in perpetuity. It is made available under aCC-BY 4.0 International license.

9. *Calculate Bundle Height and Save Bundle Height buttons:*

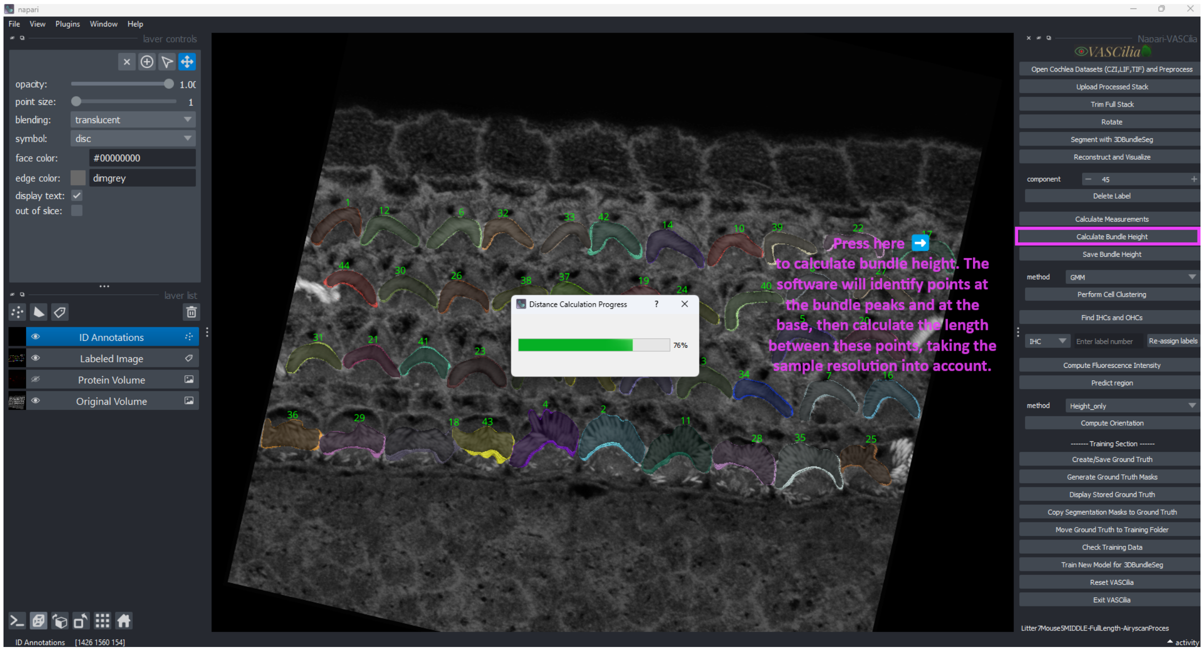

Figure 31. Screenshot of the software with the bundle height calculation action.

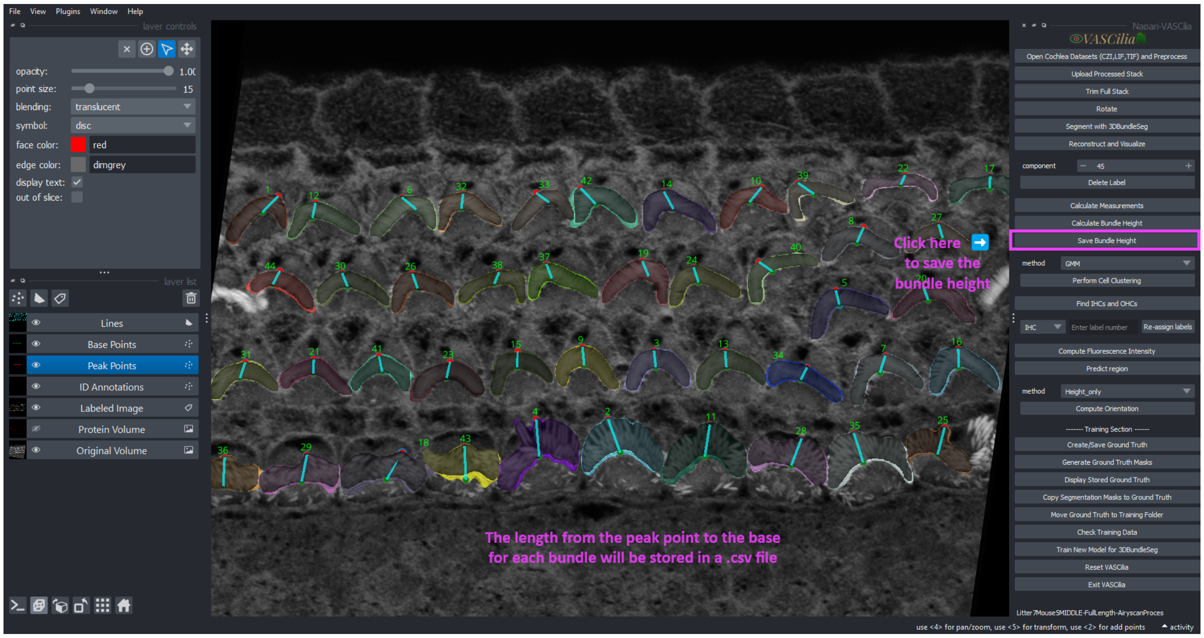

Figure 32. Screenshot of the software with the bundle height action, showing the peak points in red, base points in green, and the line connecting them in cyan.

bioRxiv preprint doi: https://doi.org/10.1101/2024.06.17.599381; this version posted February 15, 2025. The copyright holder for this preprint (which was not certified by peer review) is the author/funder, who has granted bioRxiv a license to display the preprint in perpetuity. It is made available under aCC-BY 4.0 International license.

10. Perform Cell Clustering button:

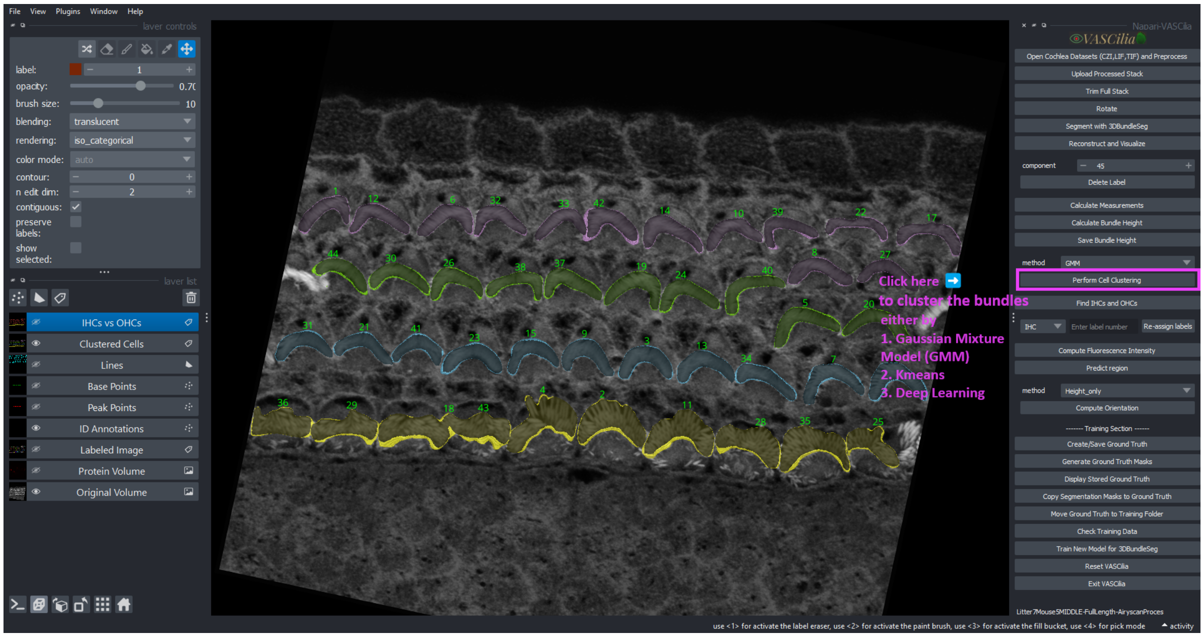

Figure 33. Screenshot of the software with the cell type identification button and Gaussian Mixture Model (GMM) results.

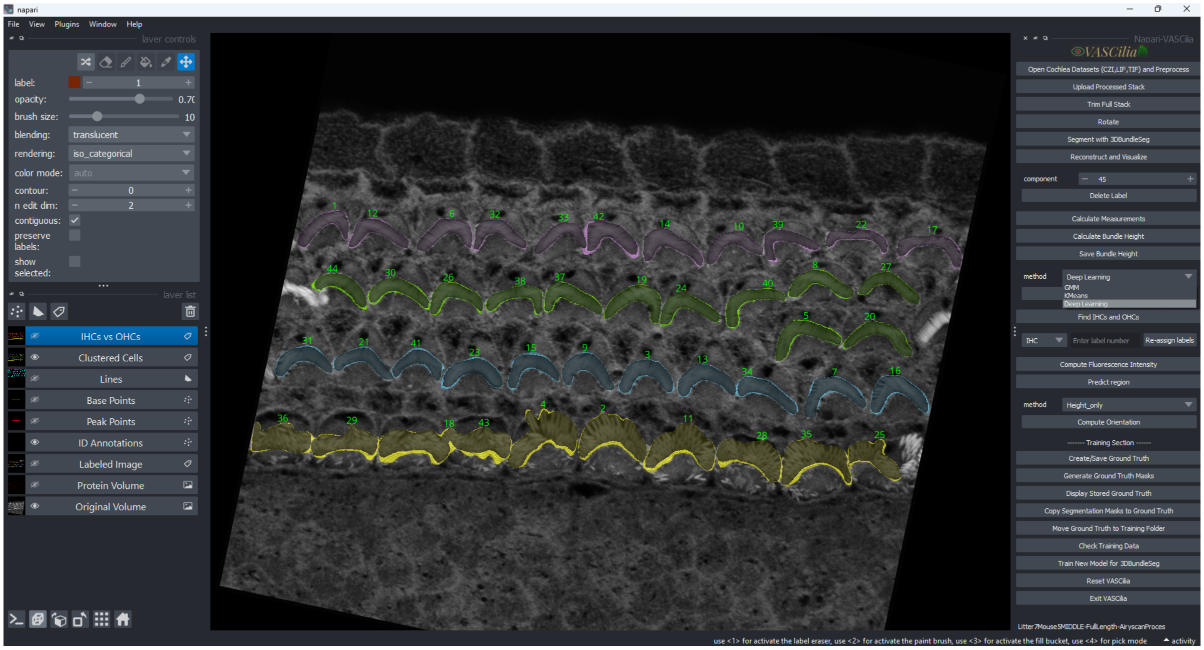

Figure 34. Screenshot of the software with the cell type identification button and Deep Learning results.

bioRxiv preprint doi: https://doi.org/10.1101/2024.06.17.599381; this version posted February 15, 2025. The copyright holder for this preprint (which was not certified by peer review) is the author/funder, who has granted bioRxiv a license to display the preprint in perpetuity. It is made available under aCC-BY 4.0 International license.

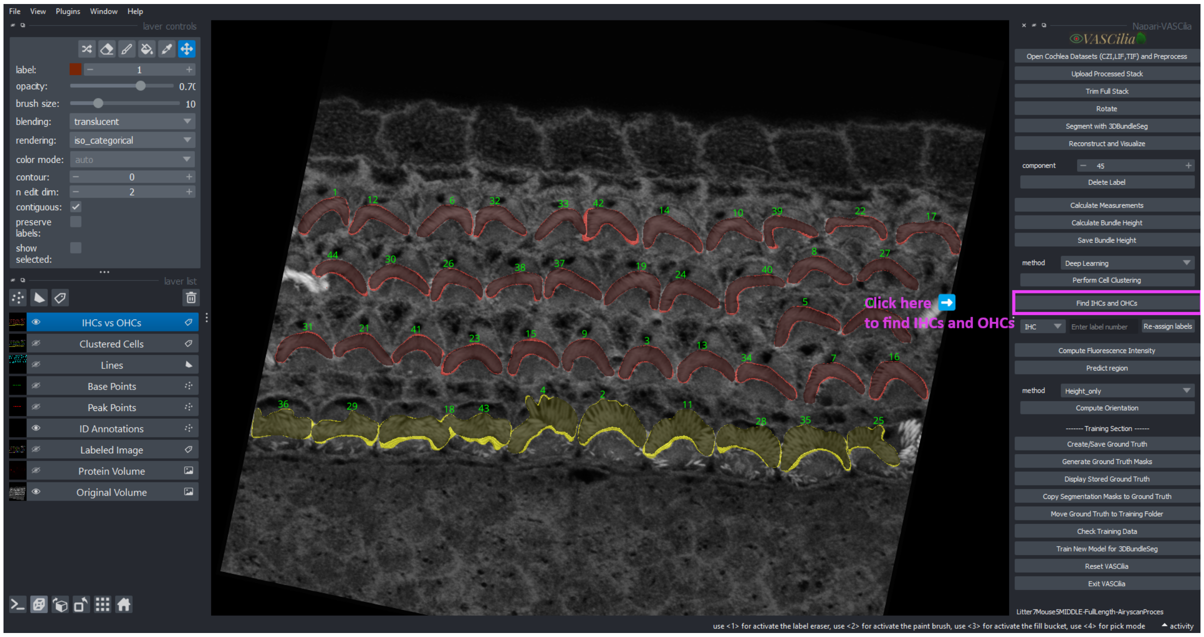

Figure 35. Screenshot of the software with the cell type identification process to distinguish between IHCs and OHCs.

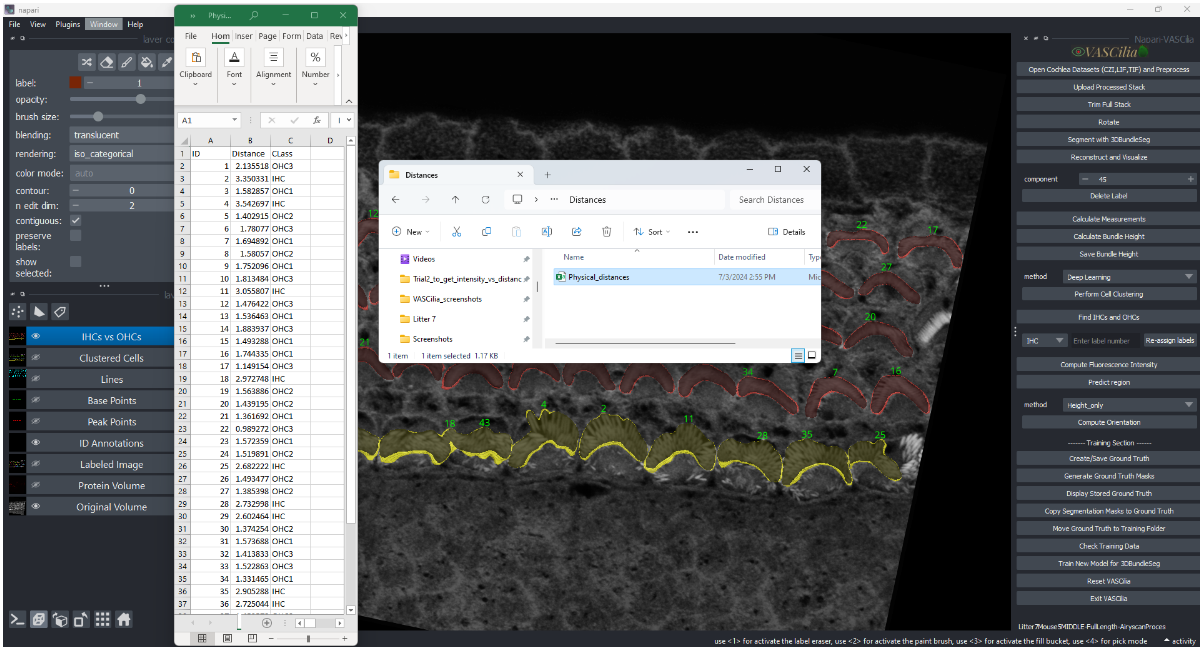

Figure 36. Screenshot of the software showing the distance.csv file updated with a new column called Class based on the cell type identification results.

bioRxiv preprint doi: https://doi.org/10.1101/2024.06.17.599381; this version posted February 15, 2025. The copyright holder for this preprint (which was not certified by peer review) is the author/funder, who has granted bioRxiv a license to display the preprint in perpetuity. It is made available under aCC-BY 4.0 International license.

11. Compute Fluorescence Intensity button:

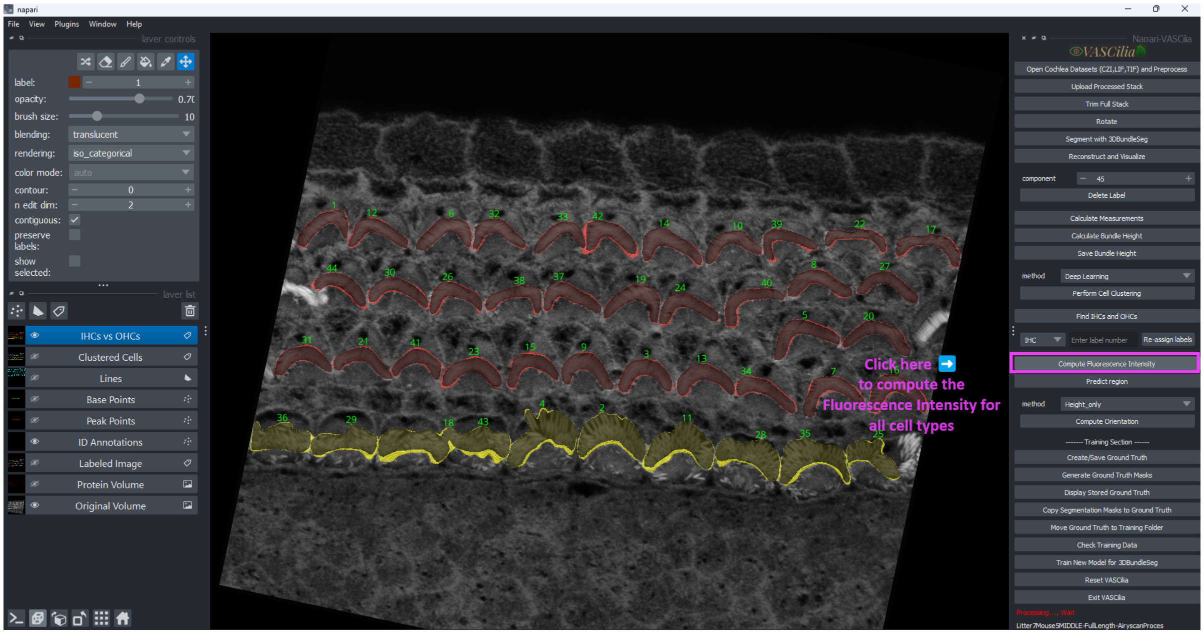

Figure 37. Screenshot of the software with the Compute Fluorescence Intensity action.

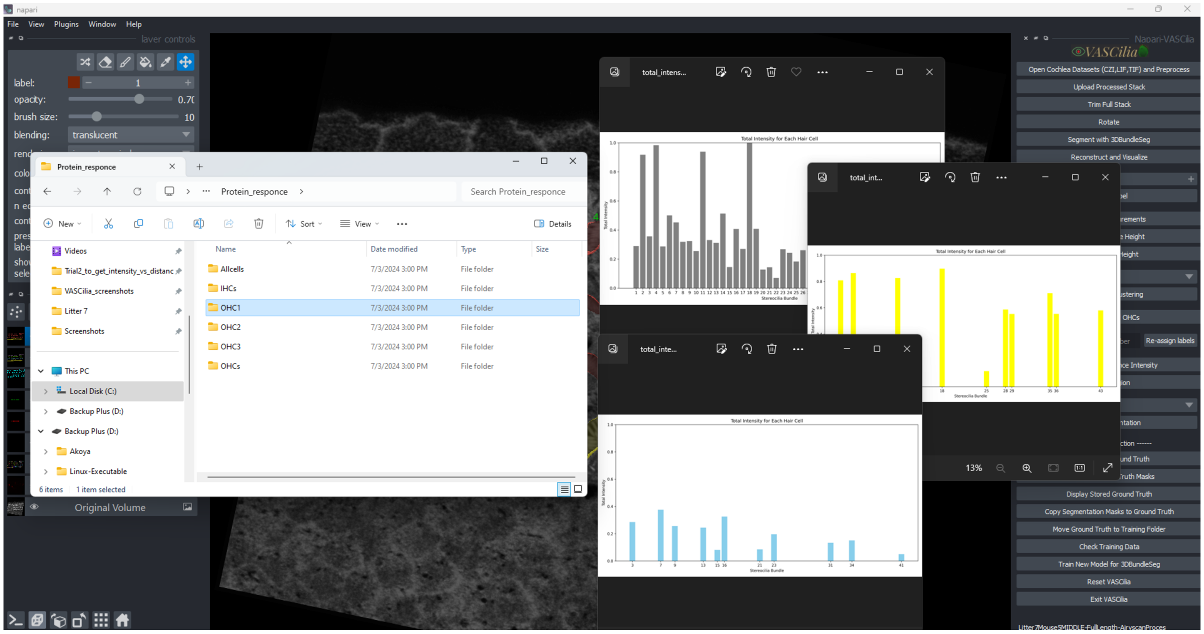

Figure 38. Screenshot of the software with the Compute Fluorescence Intensity action showing the bar plots for each cell type and the .csv file.

bioRxiv preprint doi: https://doi.org/10.1101/2024.06.17.599381; this version posted February 15, 2025. The copyright holder for this preprint (which was not certified by peer review) is the author/funder, who has granted bioRxiv a license to display the preprint in perpetuity. It is made available under aCC-BY 4.0 International license.

12. Predict Region button:

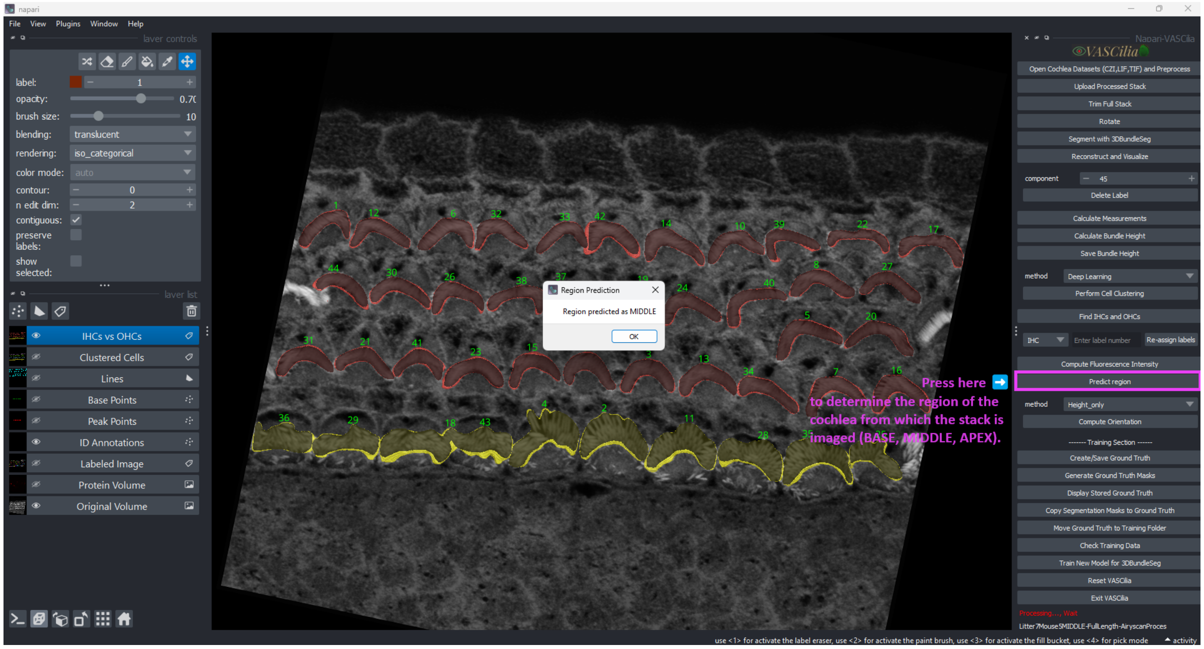

Figure 39. Screenshot of the software with the predict region action.

13. Compute Orientation button:

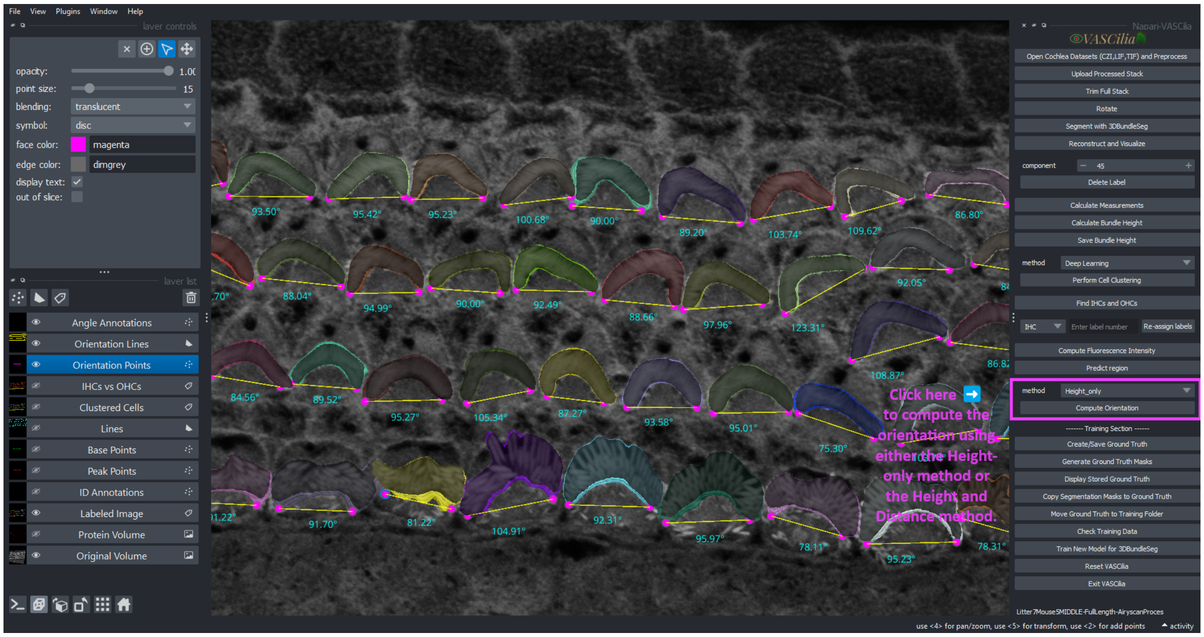

Figure 40. Screenshot of the software with the orientation measurement action.

bioRxiv preprint doi: https://doi.org/10.1101/2024.06.17.599381; this version posted February 15, 2025. The copyright holder for this preprint (which was not certified by peer review) is the author/funder, who has granted bioRxiv a license to display the preprint in perpetuity. It is made available under aCC-BY 4.0 International license.

14. Create/Save Ground Truth button:

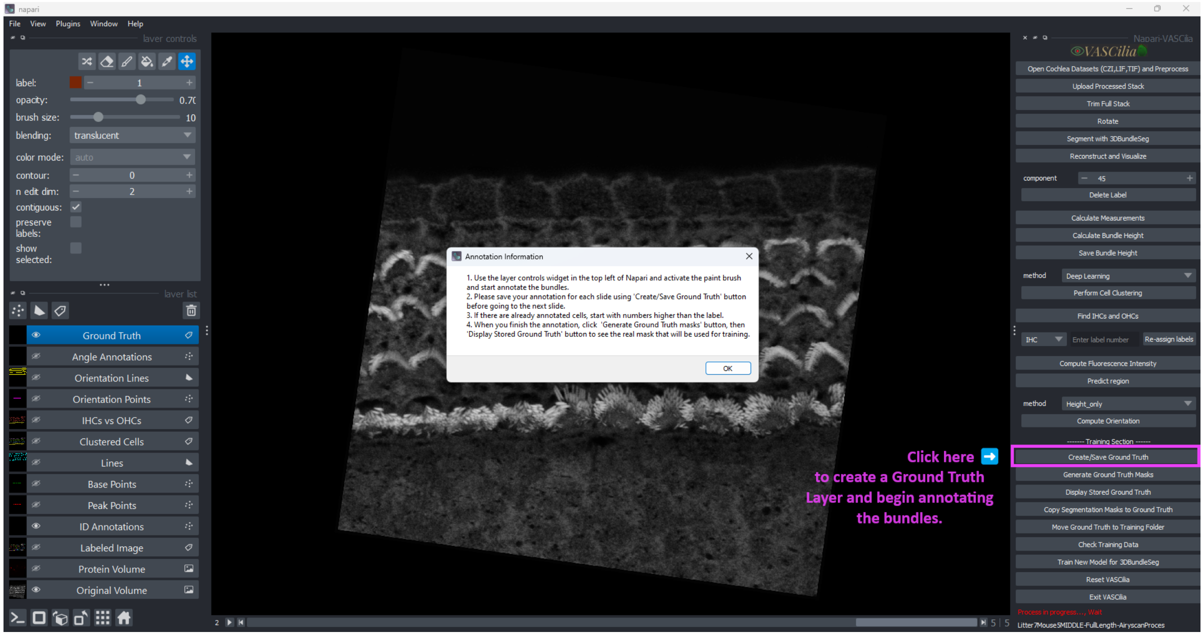

Figure 41. Screenshot of the software with the "Create GT" action

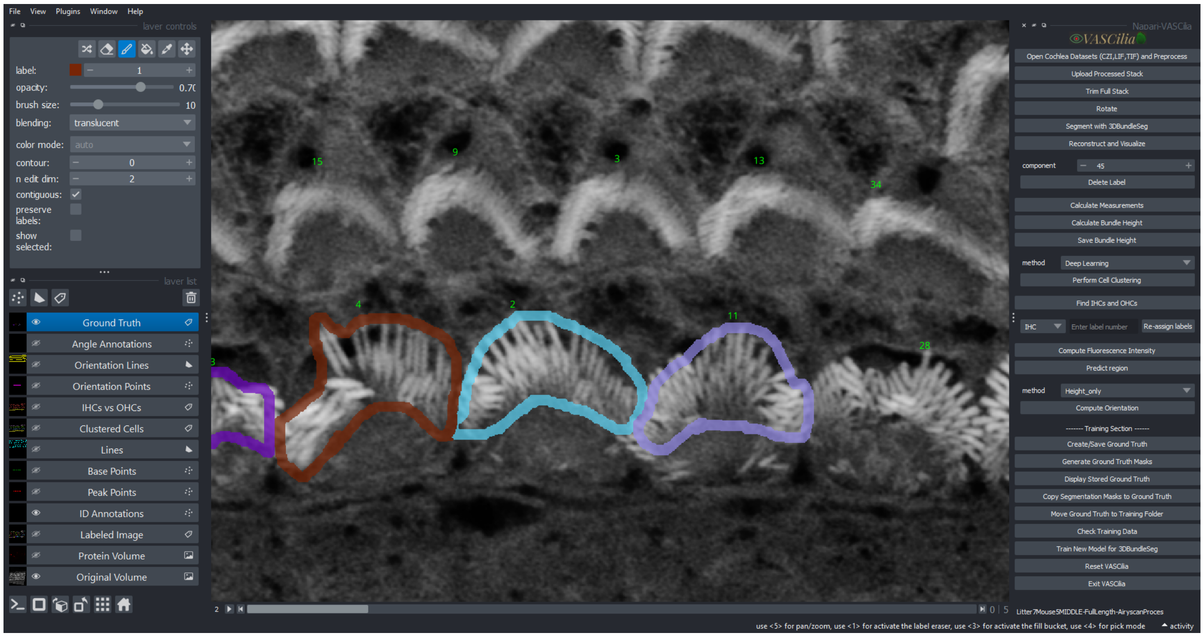

Figure 42. Screenshot of the software showing cells being annotated.

bioRxiv preprint doi: https://doi.org/10.1101/2024.06.17.599381; this version posted February 15, 2025. The copyright holder for this preprint (which was not certified by peer review) is the author/funder, who has granted bioRxiv a license to display the preprint in perpetuity. It is made available under aCC-BY 4.0 International license.

15. Display Stored Ground Truth button:

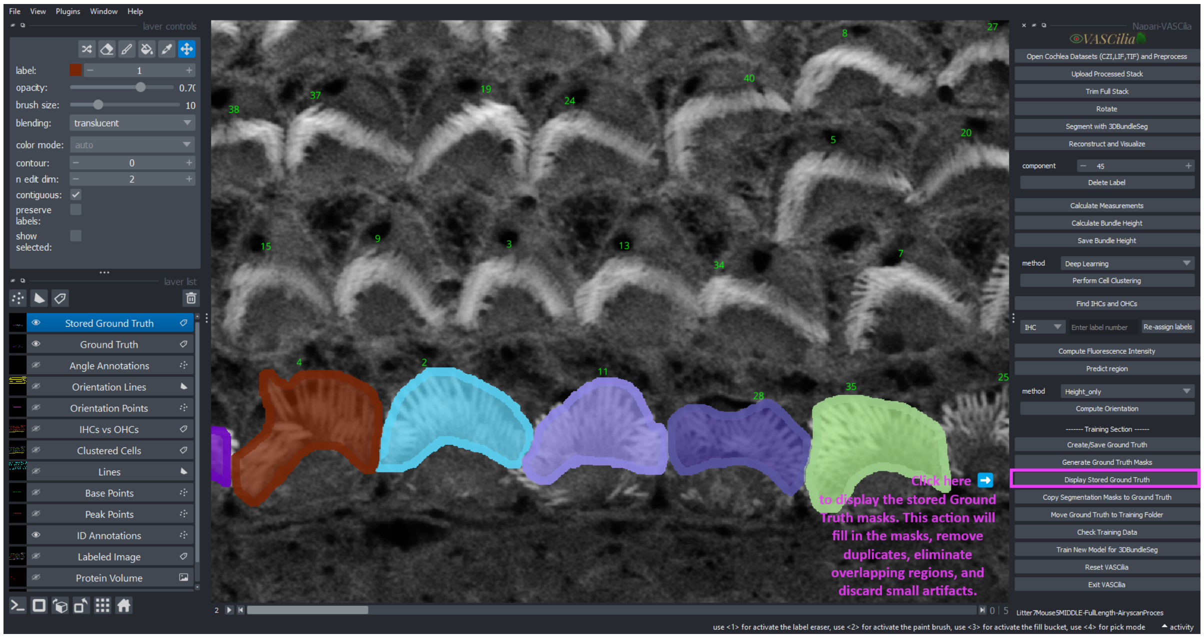

Figure 43. Screenshot of the software with the Display Stored Ground Truth action.

16. Copy Segmentation masks to Ground Truth button:

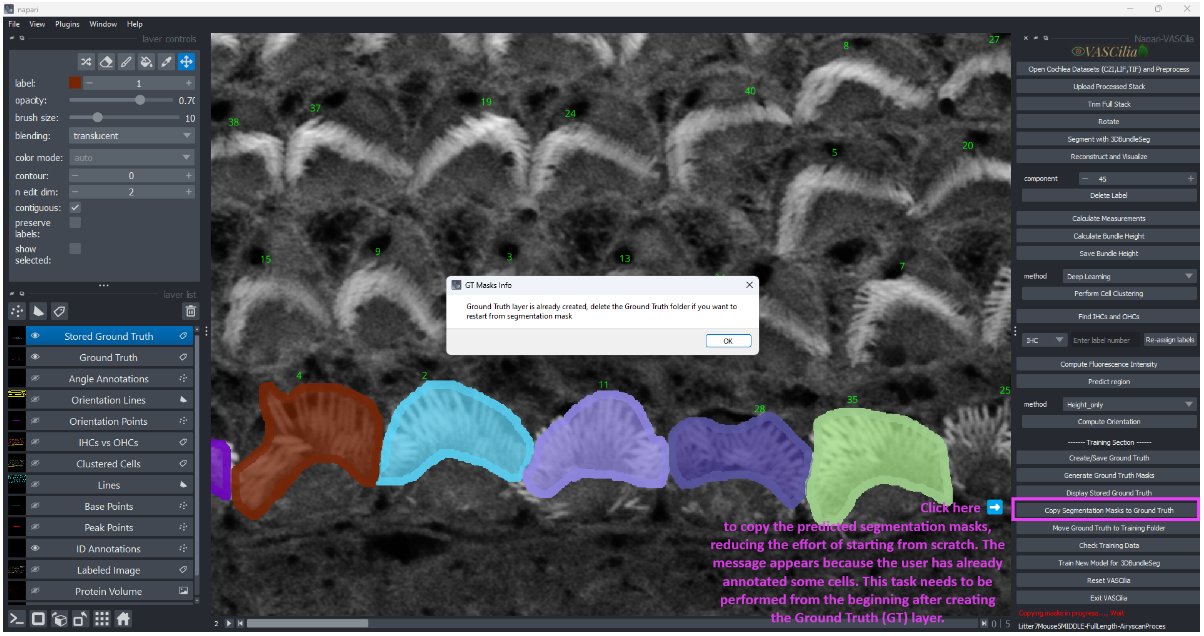

Figure 44. Screenshot of the software with the Copy Masks action, which copies the predicted masks to the GT layer.

bioRxiv preprint doi: https://doi.org/10.1101/2024.06.17.599381; this version posted February 15, 2025. The copyright holder for this preprint (which was not certified by peer review) is the author/funder, who has granted bioRxiv a license to display the preprint in perpetuity. It is made available under aCC-BY 4.0 International license.

17. Check Training Data and Train a new model for 3DBundleSeg:

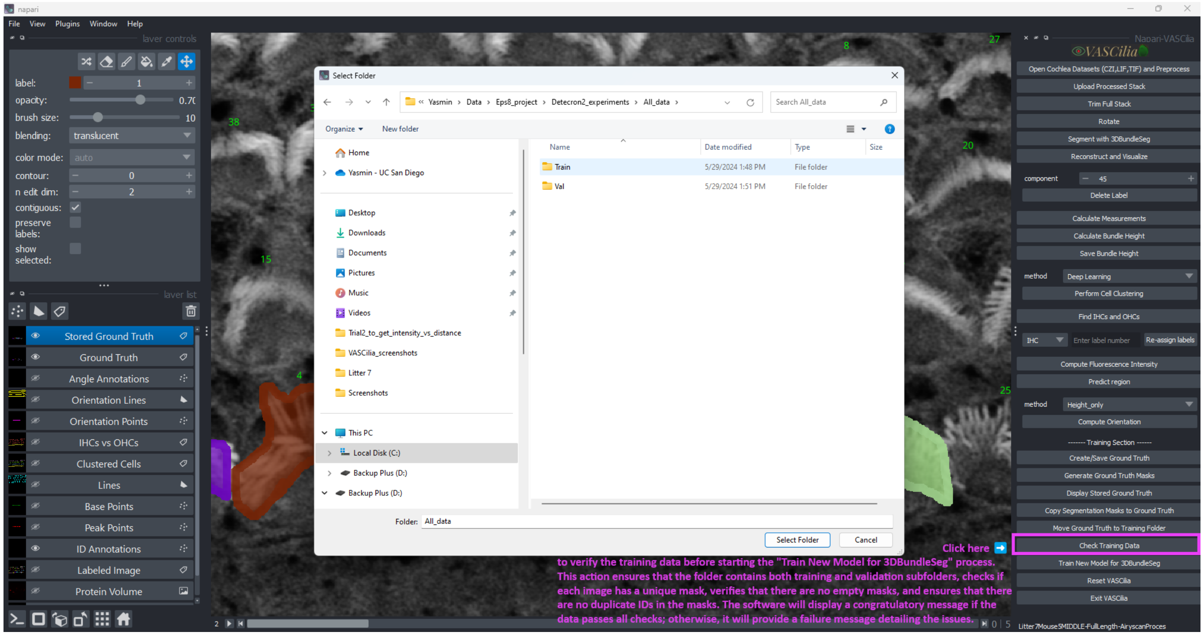

Figure 45. Screenshot of the software with the Check Training Data action.

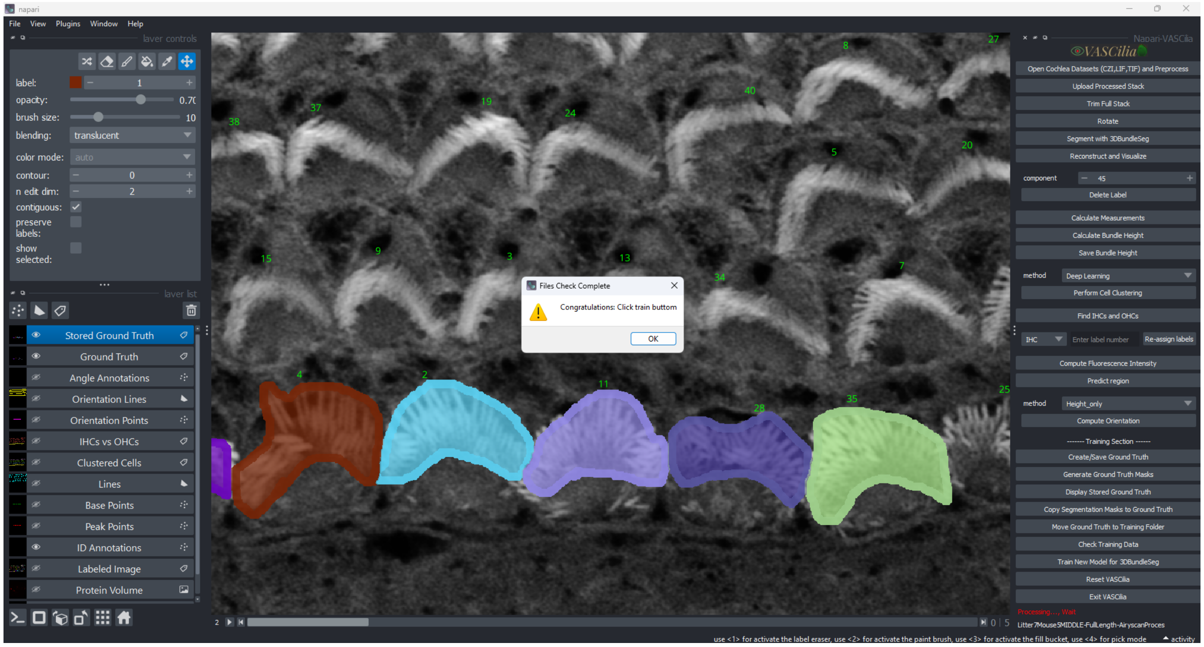

Figure 46. Screenshot of the software with the Check Training Data action button showing a Congratulation message.

bioRxiv preprint doi: https://doi.org/10.1101/2024.06.17.599381; this version posted February 15, 2025. The copyright holder for this preprint (which was not certified by peer review) is the author/funder, who has granted bioRxiv a license to display the preprint in perpetuity. It is made available under aCC-BY 4.0 International license.

18. Reset and Exit VASCilia:

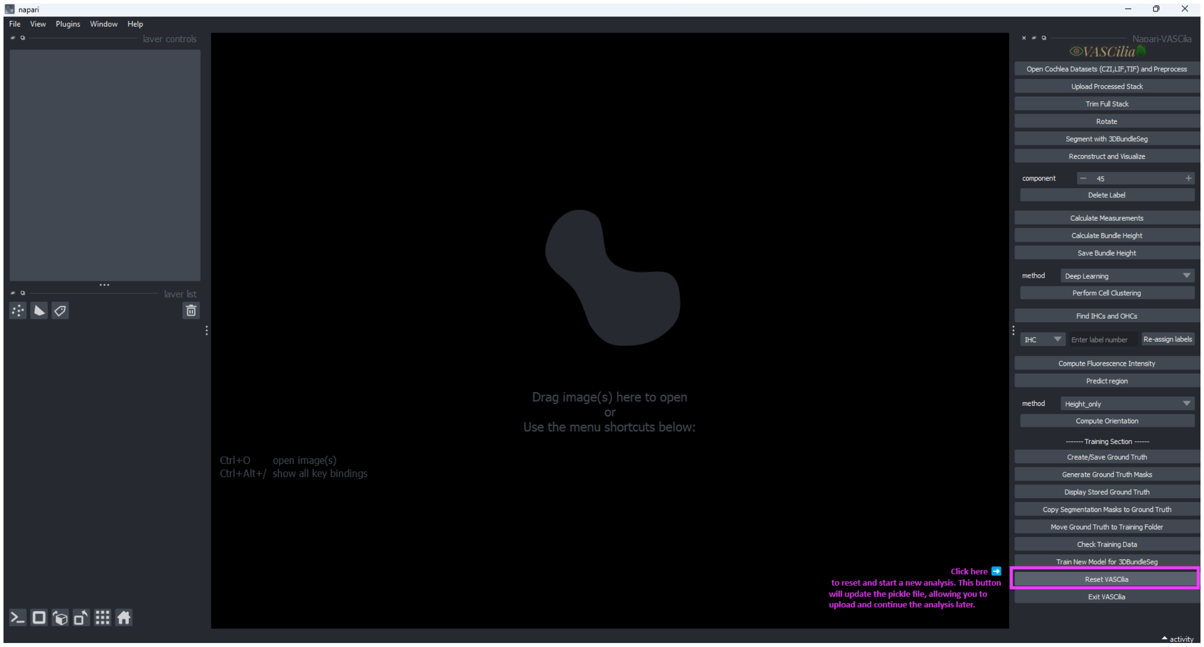

Figure 47. Screenshot of the software with the Reset button.

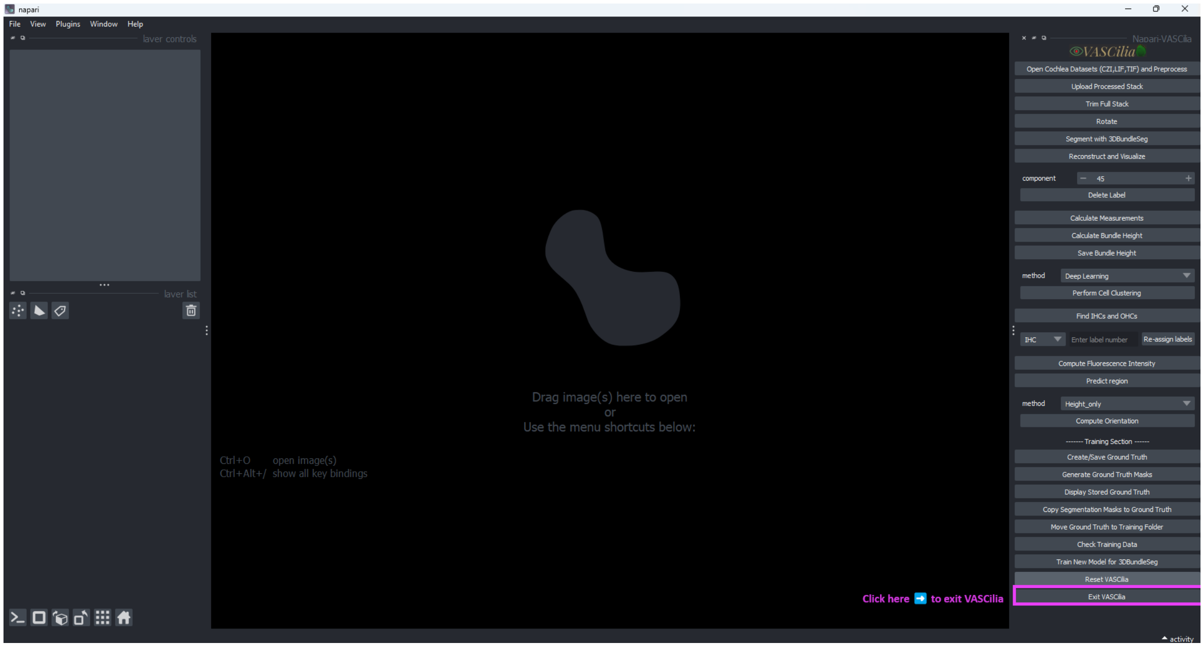

Figure 48. Screenshot of the software with the exit button.

bioRxiv preprint doi: https://doi.org/10.1101/2024.06.17.599381; this version posted February 15, 2025. The copyright holder for this preprint (which was not certified by peer review) is the author/funder, who has granted bioRxiv a license to display the preprint in perpetuity. It is made available under aCC-BY 4.0 International license.

VASCilia analysis for Artur Indzhykulian lab datasets: screenshots for various samples.

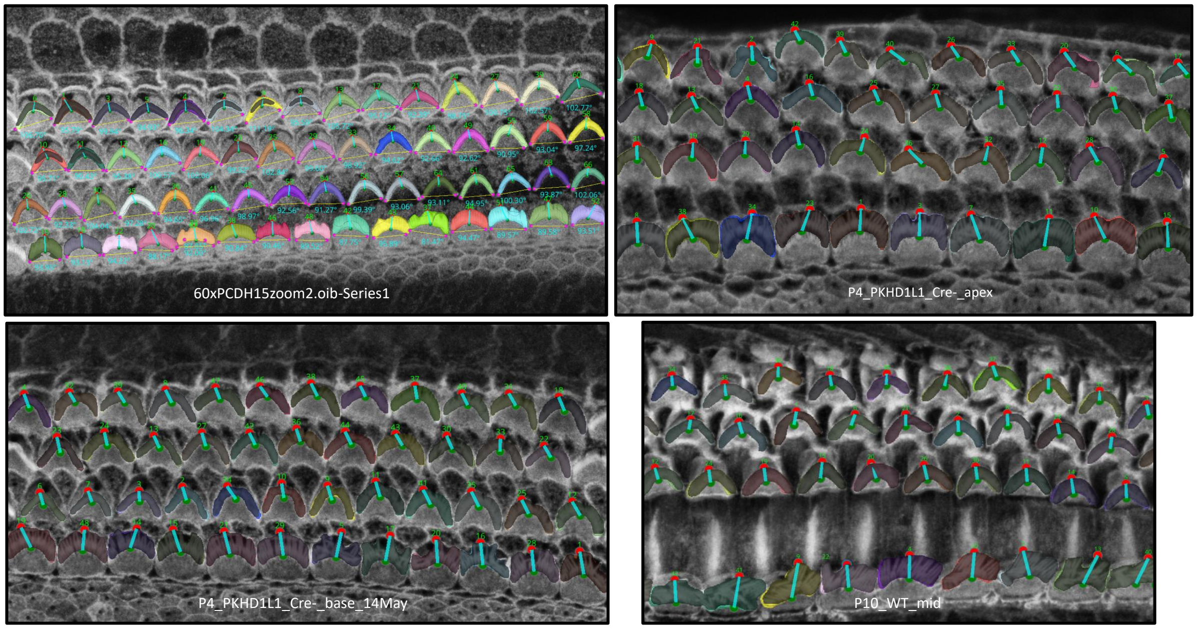

Figure 49. Screenshot of the software with different samples.

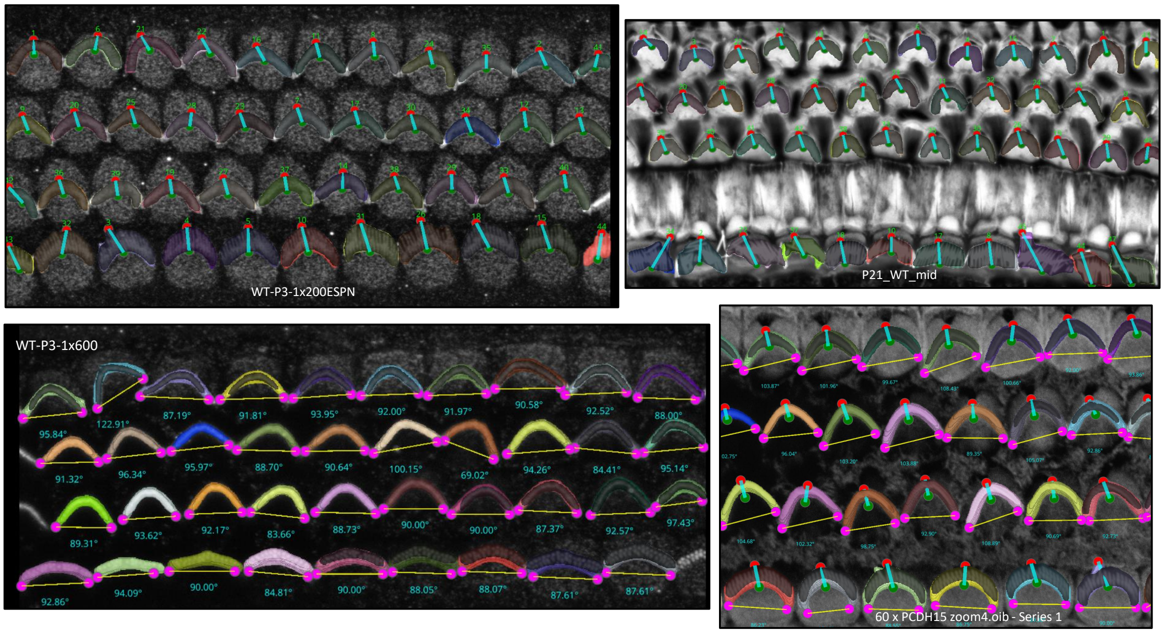

Figure 50. Screenshot of the software with different samples.

bioRxiv preprint doi: https://doi.org/10.1101/2024.06.17.599381; this version posted February 15, 2025. The copyright holder for this preprint (which was not certified by peer review) is the author/funder, who has granted bioRxiv a license to display the preprint in perpetuity. It is made available under aCC-BY 4.0 International license.

VASCilia analysis for Liberman lab datasets: screenshots for three samples.

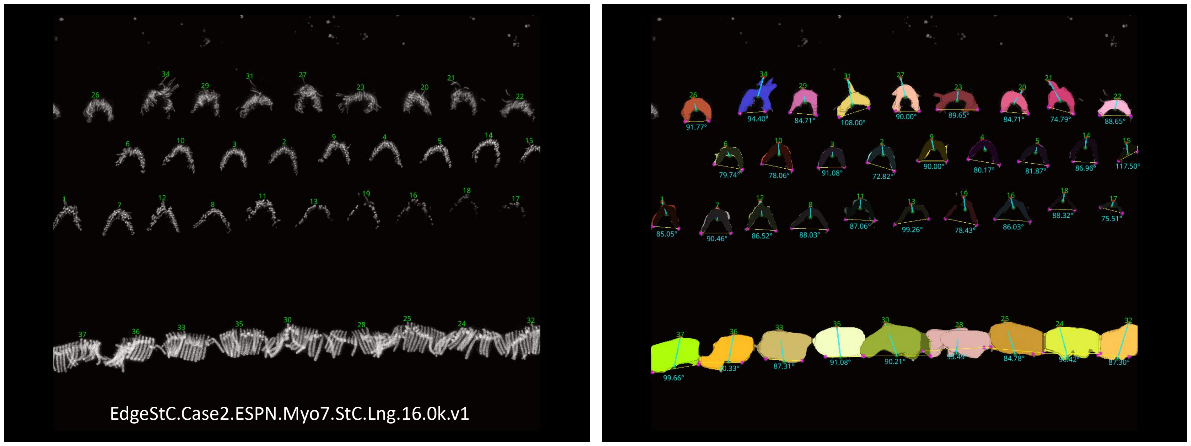

Figure 51. Screenshot of the software with Liberman lab mouse cochlea data.

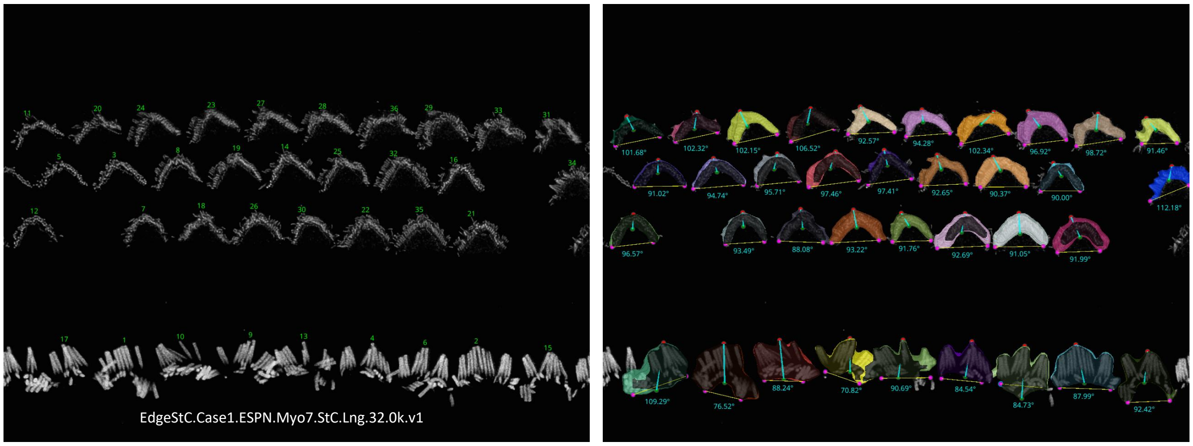

Figure 52. Screenshot of the software with Liberman lab mouse cochlea data.

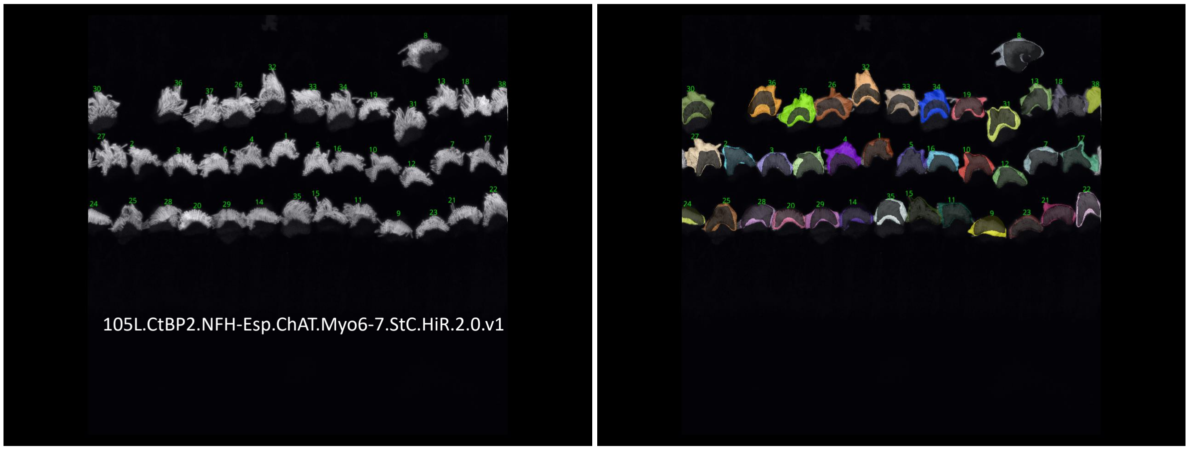

Figure 53. Screenshot of the software with Liberman lab human cochlea data.

bioRxiv preprint doi: https://doi.org/10.1101/2024.06.17.599381; this version posted February 15, 2025. The copyright holder for this preprint (which was not certified by peer review) is the author/funder, who has granted bioRxiv a license to display the preprint in perpetuity. It is made available under aCC-BY 4.0 International license.

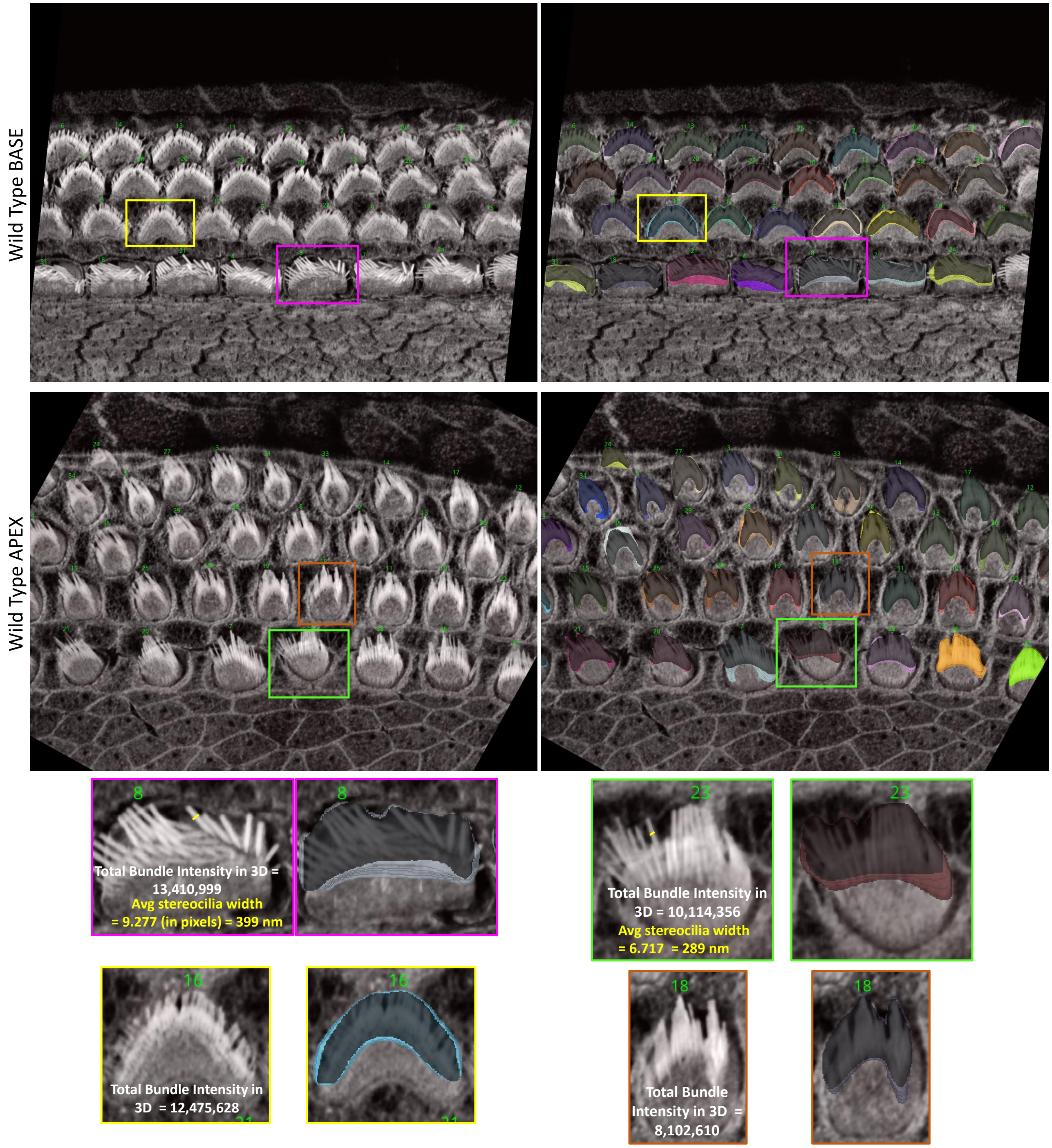

Figure 54. Visual comparison of base and apex bundles in WT animals reveals that the base appears stiffer, with wider stereocilia and bundles, while the apex displays finer stereocilia and tighter bundles, leading to a reduced phalloidin intensity signal.
